# Targeted Deletion in the Basal Body Protein Talpid3 Leads to Loss of Primary Cilia in Embryonic Stem Cells and Defective Lineage-Specific Differentiation

**DOI:** 10.3390/cells13231957

**Published:** 2024-11-25

**Authors:** Ross Ferguson, Vasanta Subramanian

**Affiliations:** Department of Life Sciences, University of Bath, Building 4 South, Bath BA2 7AY, UK; ross.ferguson@ucl.ac.uk

**Keywords:** mouse ES cells, primary cilia, talpid 3, differentiation, extraembryonic membranes

## Abstract

Talpid3 is a basal body protein required for the formation of primary cilia, an organelle involved in signal transduction. Here, we asked if Talpid3 has a role in the regulation of differentiation and/or self-renewal of ES cells and whether cells lacking cilia due to a deletion in Talpid3 can be reprogrammed to induced pluripotent stem (iPS) cells. We show that mouse embryonic limb fibroblasts which lack primary cilia with a targeted deletion in the *Talpid3* (*Ta3*) gene can be efficiently reprogrammed to iPS cells. Furthermore, vector-free *Ta3^−/−^* iPS cells retain ES cell features and are able to self-renew. However, both *Ta3^−/−^* iPS and ES cells are unable to form visceral endoderm and differentiate poorly into neurons. The observed defects are not a consequence of reprogramming since *Ta3^−/−^* ES cells also exhibit this phenotype. Thus, Talpid3 and primary cilia are required for some differentiation events but appear to be dispensable for stem cell self-renewal and reprogramming.

## 1. Introduction

Primary cilia are non-motile cell structures that protrude from the apical cell surface and consist of a basal body and an axoneme which has a 9 + 0 microtubule arrangement [1,2]. The receptor-rich ciliary membrane makes them key sensors and transducers of signalling pathways such as hedgehog (Hh), Notch and PDGF, which play important roles in developmental processes [2,3,4]. These signals are transduced through factors which either become activated in the cilia or in the basal body scaffold proteins [5,6,7,8,9] leading to downstream effects.

Cilia-dependent signalling plays a major role in early cell fate determination [10,11] as well as in later developmental processes. For example, Hh signalling plays a role in neurogenesis, patterning of the spinal cord and heart development [12,13,14]. Thus, any defects in or loss of primary cilia can affect multiple developmental processes [15,16,17,18].

Mutations in the components of the cilium or its basal body manifest as clinical conditions collectively referred to as ciliopathies [19,20]. Patients with ciliopathies exhibit defects such as polycystic kidneys, loss of hearing, abnormal heart looping and polydactyly [19,20,21,22].

*Talpid3* (*Ta3*) was originally identified in the chick *talpid3* mutant in which a frameshift in the *talpid3* gene leads to a truncated protein [23]. In the *talpid3* chick and the *Ta3* mouse mutant, the centrosome, which will form the basal body, fails to dock with the apical cell membrane, leading to failure of primary cilia formation [24,25]. The defective docking seen in *Ta3* mutant cells is also seen in cells lacking other basal body proteins such as *OFD1* (oral-facial-digital syndrome 1 [26]) and *MKS1* (Meckel syndrome 1 [27]).

*Ta3* is located at the distal tip of centrioles [28] and is a component of the CP110-containing protein complex. Its recruitment to this complex is dependent on its interaction with CEP120 [29]. *Ta3* has also been shown to interact with the basal body components C2CD3 and CEP290, which have been suggested to have overlapping but distinct functions in the formation of the ciliary vesicle, a critical step in ciliogenesis [28,29,30].

The loss or disruption of *Ta3* has severe developmental consequences. The *talpid3* chick cells lack primary cilia and have defective hedgehog signalling, resulting in polydactyly. They are also embryonic lethal [24]. *Ta3* has a coiled coil domain, which is highly conserved [24]. Constitutive deletion of exons 11 and 12, which encode this domain in mice, results in early embryonic lethality. Cells in these mice lack primary cilia, and the conditional deletion of exons 11 and 12 of *Ta3* in the limb leads to polydactyly and abnormal Shh signalling [25] while deletion in the central nervous system causes cerebellar hypoplasia and ataxia (key features of Joubert syndrome- a ciliopathy) with abnormal Shh signalling [31]. Targeted mutation of the *talpid3* gene in zebrafish also leads to loss of cilia and defective Shh signalling [32]. Mutations in the human *TALPID3* gene (*KIAA0586*) have been found in Joubert syndrome and Jeune asphyxiating thoracic dystrophy [33,34,35,36].

Primary cilia are present in mouse and human embryonic stem cells (mESCs and hESCs), both in actively growing cells and under growth arrest conditions [37,38]. This, in conjunction with reports showing that components of the Hh pathway (Ptch, Smo, and Gli2) are present in both hES [37] and mES cells [10,11], suggest that primary cilia may be involved in the maintenance of pluripotency/self-renewal and/or the regulation and coordination of the first steps of ESC differentiation. Since ES cells and induced pluripotent stem (iPS) cells are invaluable models for studying mechanisms involved in differentiation with implications for applications in regenerative medicine [39], it is important to determine if the reprogramming of somatic cells, maintenance of pluripotency/self-renewal or differentiation to specific early/late lineages are controlled by the machinery of the primary cilia.

We investigated if embryonic limb fibroblasts from mice with a targeted deletion of the conserved exons 11 and 12 of *Talpid3* (*Ta3^−/−^*), which lack primary cilia, could be reprogrammed to induced pluripotent stem cells (iPSCs) and if they respond to early and late differentiation cues. In order to rule out the possibility that defects seen in *Ta3^−/−^* iPS cells could be due to incomplete reprogramming, we also established and characterised *Ta3^−/−^* and *Ta3^fl/fl^* ES cell lines compared the stemness, proliferation and differentiation potential of *Ta3^−/−^* ES/iPS cells with the *Ta3^fl/fl^* ES/iPS cells.

## 2. Results

### 2.1. Reprogramming of Embryonic and Limb Fibroblasts to iPS Cells and Their Characterisation

**Figure 1:**
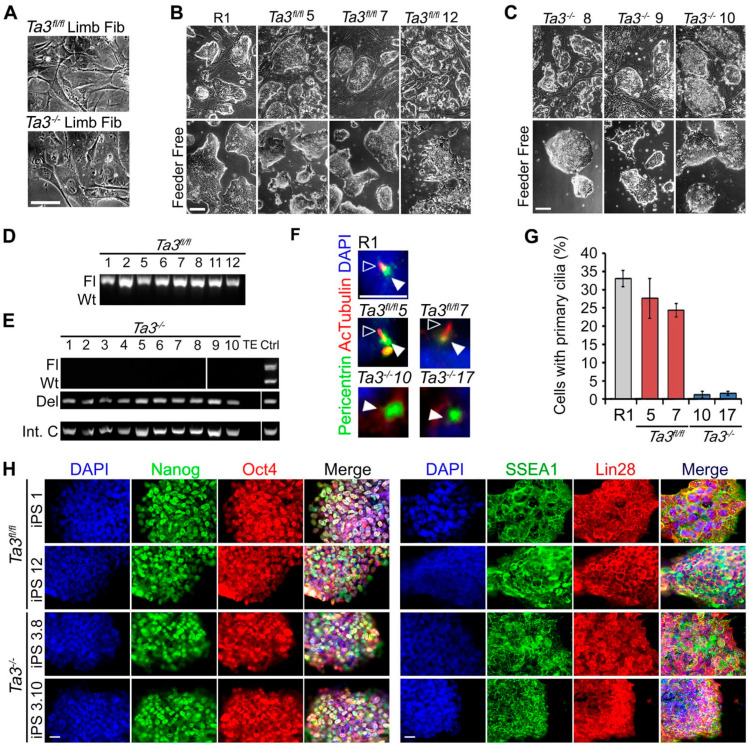
*Ta3^fl/fl^* and *Ta3^−/−^* mouse embryo fibroblasts and pre-excision iPS cells. (**A**) Limb fibroblasts from E12.5 *Ta3^−/−^* and *Ta3^fl/fl^* mouse embryos. Scale bar: 50 µm. (**B**) Upper Panel: feeder-dependent *Ta3^fl/fl^* iPS cell lines; Lower Panel: feeder-independent *Ta3^fl/fl^* iPS cells. Scale bar: 100 µm. (**C**) Upper panel: feeder dependent *Ta3^−/−^* iPS cell lines; Lower Panel: feeder independent *Ta3^−/−^* iPS cells Scale bar: 100 µm. (**D**) Genotype of *Ta3^fl/fl^* iPS cells (Fl, floxed allele; Wt, wildtype allele). positive control ‘Ctrl’ shared with panel E. (**E**) Genotype of *Ta3^−/−^* iPS cells; (Del, deleted allele; Int.C, internal control PCR for an unrelated region; Ctrl, positive PCR control using mouse gDNA—*Ta3^+/fl^* for Fl/WT PCR and *Ta3^+/−^* mouse for Del PCR). (**F**) Primary cilia in R1 ES and *Ta3* iPS immunostained with antibody to pericentrin and acetylated tubulin to identify the centrosome (black arrows) and axoneme of primary cilia (white arrows). Scale bar: 10 µm. (**G**) Quantification of primary cilia. *Ta3^fl/fl^* cells (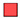), R1 (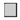), *Ta3^−/−^* cells (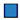). Error bars ± SEM, *n* ≥ 350 cells from three independent experiments. (**H**) Expression of pluripotency markers Nanog, Oct4, SSEA1 and Lin28 in *Ta3^fl/fl^* and *Ta3^−/−^* iPS cells. Scale bar: 25 µm.

The constitutive deletion of exons11–12 in *Ta3* results in embryonic lethality around E9.5. Therefore, in order to obtain fibroblasts for reprogramming, we crossed *Ta3^fl/fl^* mice with mice carrying a Cre recombinase transgene driven by the limb-specific *Prrx1* promoter. Limb fibroblasts were isolated from both *Ta3^fl/fl^ Prrx1-Cre* and *Ta3^fl/fl^* (control) embryos at E12.5 (Figure 1A) and reprogrammed to induced pluripotent stem (iPS) cells [40] using the *piggyBac* transposon-based reprogramming vector *OSKML* [41]. Reprogrammed colonies appeared by 4–5 days and were well established by 8–10 days. The efficiency of reprogramming for both *Ta3^fl/fl^* and *Ta3^−/−^* embryonic limb fibroblasts was comparable to that reported previously [41], with no obvious difference in the reprogramming efficiency between them.

The *piggyBac* system allows us to excise the reprogramming cassette through retransfection with the transposase in order to create footprint-free iPSC lines. Before proceeding with excision, the established iPSC lines were characterised in terms of morphology, marker expression and transgene copy number to identify the clones most suitable for further study.

Both *Ta3^fl/fl^* and *Ta3^−/−^* iPS cell lines exhibited characteristic ES cell morphology—highly refractile cells with a high nucleus-to-cytoplasm ratio that grow as tightly packed colonies and reform upon passaging (Figure 1B,C). The iPS cell lines were genotyped to confirm the presence or absence of the *Ta3* exons 11 and 12 by PCR (Figure 1D,E). The *Ta3^−/−^* iPS lines lacked primary cilia (Figure 1F,G), which were present in the *Ta3^fl/fl^* cell lines. The *Ta3^fl/fl^*, *Ta3^−/−^* iPS cell lines and the reference mES cell line R1 all expressed mESC-associated pluripotency markers as seen by both RT-PCR (*Oct4*, *Sox2*, *c-Myc*, *Dppa3*, *Dnmtl3*, *Nanog*, *Rex1* and *Zfp296*; Figure S1A) and immunostaining (Figure 1H—Oct4, Nanog, Lin28 and SSEA1).

### 2.2. Establishment of Transgene-Free IPS Cell Lines by Excision of the PiggyBac Reprogramming Vector

Excision of the reprogramming vector is easier in cell lines which have fewer integrated copies. The copy numbers of the integrated *piggyBac* reprogramming vector were first determined by qPCR and were found to range from 1 to 15 copies in the clones tested (Figure S1B). The integration sites were further characterised by splinkerette PCR and sequencing [41] in the clones carrying the lowest copy number. iPS cell lines in which the integration sites were not in any key functional loci were chosen for vector excision (Table S1).

Two independent iPS cell lines from each of the *Ta3^−/−^* and *Ta3^fl/fl^* genotypes carrying an estimated 1–2 copies of the reprogramming vector (Figure S1B) were electroporated with the transposase *mPB* [41] to induce excision. Excision of the reprogramming *piggyBac* vector proved difficult, and excised clones from the *Ta3^−/−^* and the *Ta3^fl/fl^* iPS lines were obtained at a low frequency (Figure S1C).

**Figure 2:**
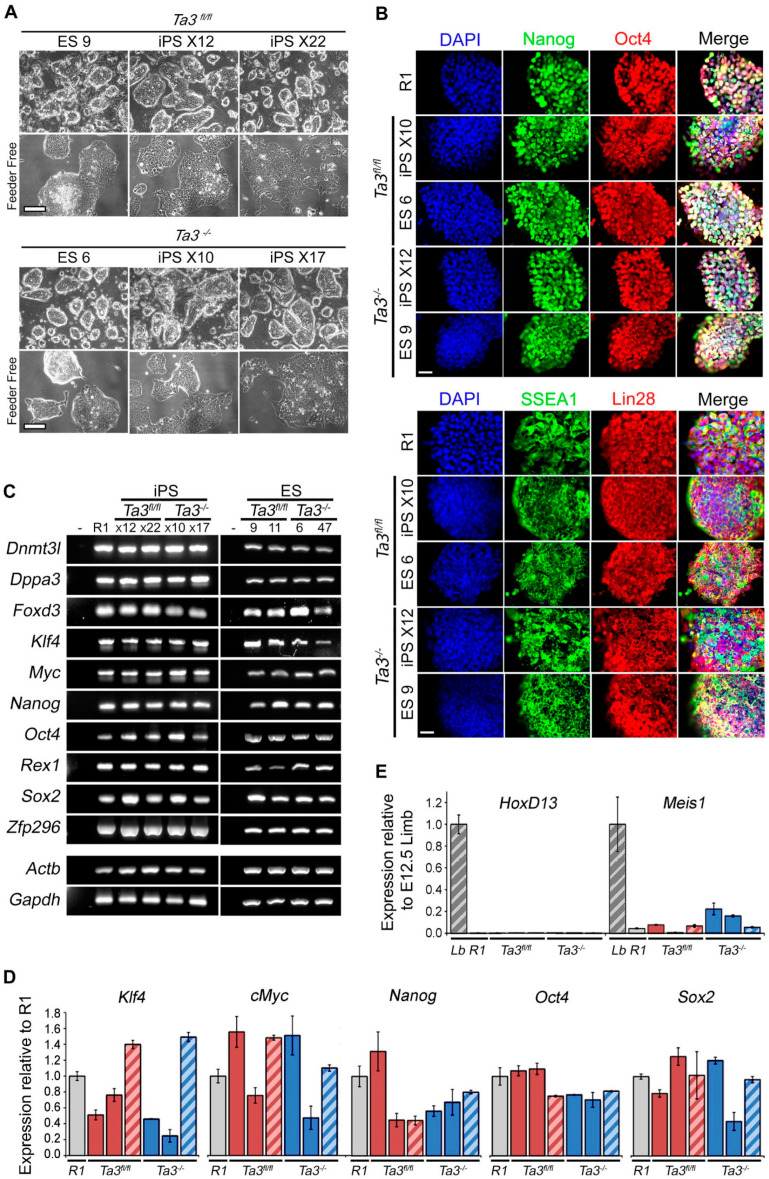
Expression of pluripotency markers in *Ta3* ES and transgene-free *Ta3* iPS cells. (**A**) Upper panel: *Ta3* ES cells and transgene-free *Ta3* iPS cell lines maintained on MITC MEFs; Lower Panel: feeder-independent *Ta3* ES cells and transgene-free *Ta3* iPS cell lines cultured in 2i medium. Scale bar: 200 µm. (**B**) Immunostaining for the pluripotency markers Nanog, Oct4, SSEA1 and Lin28 in the *Ta3^−/−^* or *Ta3^fl/fl^* ES and iPS cell lines. Scale bar: 25 µm. (**C**) RT-PCR for a panel of pluripotency markers by *Ta3^−/−^* and *Ta3^fl/fl^* ES and transgene-free iPS cells. (**D**) qRT-PCR for selected pluripotency associated transcripts in R1 (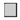), *Ta3^fl/fl^* iPS X12 and X22 (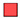), *Ta3^fl/fl^* ES 9 (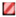), *Ta3^−/−^* iPS X10 and X17 (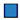), and *Ta3^−/−^* ES 6 (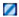). (**E**) Expression of the limb markers HoxD13 and Meis1 in ES and iPS cell lines and in E12.5 mouse limb (Lb, 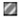).

The *Ta3^−/−^* and *Ta3^fl/fl^* transgene-free miPS cell lines appeared grossly similar in morphology to the parental lines and R1 mESCs (Figure 2A). Two clones of each genotype were taken forward for further characterisation alongside the ES cell lines established and described in the next section. These clones were designated *Ta3^fl/fl^ X12*, *Ta3^fl/fl^ X22*, *Ta3^−/−^X10* and *Ta3^−/−^ X17*, where X indicates these are transgene-free miPS lines.

### 2.3. Establishment of ES Cell Lines from Ta3^−/−^ and Ta3^fl/fl^ Blastocysts

We also established ES lines from *Ta3^−/−^* and *Ta3^fl/fl^* blastocysts in order to compare their stem cell characteristics and differentiation potential with that of the *Ta3^−/−^* and *Ta3^fl/fl^* iPS cells. ES cell lines were generated from *Ta3^−/−^* and *Ta3^fl/fl^* blastocysts using the 2i protocol as described in the Methods section. A total of 96 *Ta3^−/−^* and 36 *Ta3^fl/fl^* stable ES cell lines were established (Figure 2A), and the genotypes of the ES cell lines were confirmed by PCR. XY cell ES lines were identified by PCR for the *Sry* sequence (Figure S1D).

### 2.4. Expression of Pluripotency and Limb-Specific Markers in Ta3^−/−^ and Ta3^fl/fl^ ESCs and Transgene-Free iPSCs

The *Ta3^−/−^* and *Ta3^fl/fl^* transgene-free iPS and ES cell lines were analysed for expression of pluripotency-associated markers by immunofluorescence, RT-PCR and qPCR (Figure 2B–D) as well as by Western blot (Figure S1E,F) and compared with the reference R1 mES cell line.

Immunofluorescence and RT-PCR expression data confirmed that the integration-free iPS cell lines retained their ES cell characteristics (Figure 2B,C). Quantification of the expression of a subset of markers by qPCR also showed no significant differences in levels (Figure 2B–D). Similarly, no differences were found by Western blot (Figure S1E,F). Additionally, no higher molecular weight vector-derived T2A/F2A tagged Oct4 or Lin28 species, confirming excision in the iPSC lines (Figure S1E).

While some variations in expression are present, no genotype or clone-specific variation could be seen using any of these methods, and all cell lines appear comparable to the reference R1 mESCs. To further ensure reprogramming was complete, we also checked for embryonic limb markers, as the iPSCs were derived from mouse limb fibroblasts. *Prx1a*, *Prx1b*, *Meis1* and *Hoxd13* are expressed robustly in limb fibroblasts (Figure 2E and Figure S1G). In contrast, both R1 mESCs and *Ta3* iPSCs show low to undetectable expression. We also sought to characterise the consequences of deleting exons 11 and 12 on *Ta3* expression and found that a truncated protein product continued to be produced at low levels in both the original mouse model and the derived ES lines (Figure S1H,I). We do not expect this truncated product to be functional due to the lack of the essential coiled-coil domain, as this deletion has previously been well-characterised in the mouse model [24,25,31] and phenocopies, the original chick model [23], but we cannot definitively exclude non-primary cilia functions.

From this point forward, all iPSC lines used were transgene-free subclones and are identified by the inclusion of X for ‘excised’ in their identifier. These cell lines are *Ta3^−/−^* iPS X10 and X17 and *Ta3^fl/fl^* iPS X12 and X22.

### 2.5. Effect of Deletion in Talpid3 on Colony Morphology and Cell Cycle of Ta3^−/−^ ES and iPS Cells

*Ta3^−/−^* ES and iPS cell lines grew at a similar rate to the R1 ES and *Ta3^fl/fl^* ES and iPS cells. On closer examination of the colony morphology, we found that the *Ta3^−/−^* ES and iPS colonies had very smooth edges and grew as much tighter colonies as compared to R1 ES, *Ta3^fl/fl^* ES and iPS cells. We measured the smoothness of the colony perimeter and found there was a significant difference (Figure S2A,B). Cytoskeletal defects have been previously associated with loss of *Ta3* [24,25]. Staining for F-actin and acetylated tubulin showed disorganised staining in *Ta3^−/−^* ES and iPS cells, where they appear qualitatively less densely bundled and shorter (Figure S2C); however, we do not quantify or characterise this aspect of the *Ta3* deletion phenotype here. This staining also showed that there were no morphological defects in the mitotic phases (Figure S2D). There were also no significant differences in the cell cycle between R1 ES and *Ta3^fl/fl^* or *Ta3^−/−^* ES and iPS cells as determined by BrdU incorporation and FACS analysis (Figure S3). No differences in time to confluence or growth were noted during routine culture either, and this is consistent with the lack of gross very early developmental abnormalities observed in the homozygous constitutively deleted exon 11–12 mouse embryos prior to lethality around E9.5 [25],

### 2.6. Primary Cilia Are Absent and Basal Structures Enlarged in Transgene-Free Ta3 Exon11–12 DeletedmiPS ES Cell Lines

**Figure 3:**
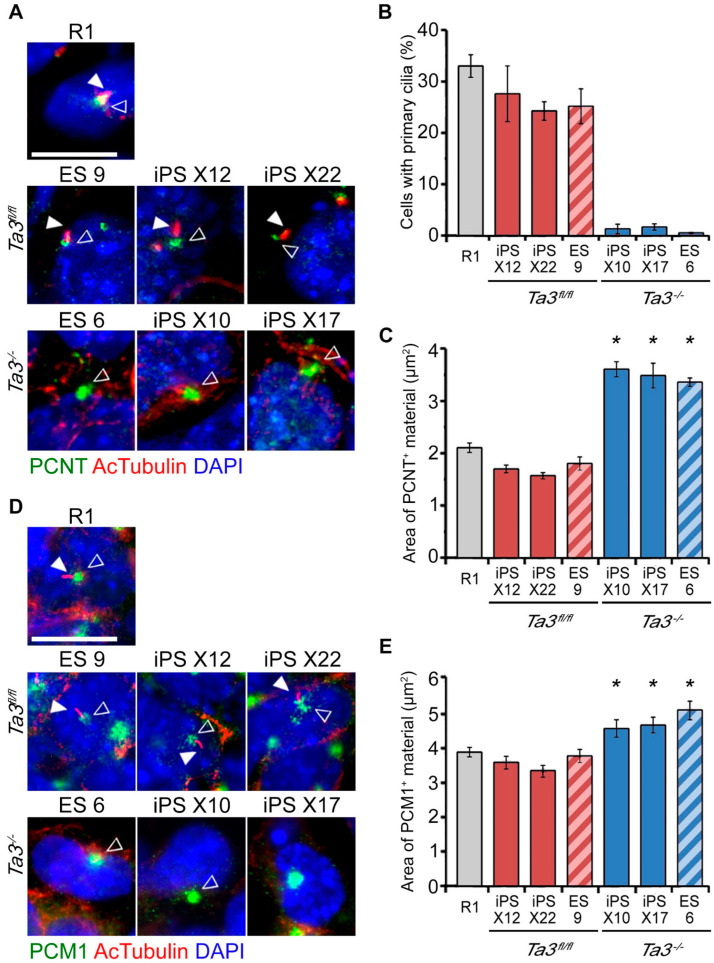
Characterisation of primary cilia in *Ta3* ES and transgene-free *Ta3* iPS cells. (**A**) Primary cilia in R1, transgene-free *Ta3^fl/fl^* X12 or X22 and *Ta3^−/−^* X10 and X17. Cells immunostained for pericentrin (PCNT, hollow arrows) to identify the centrosome) and acetylated tubulin to identify the axoneme of primary cilia (AcTubulin, white arrows). Scale bar: 10 µm. (**B**) Quantification of primary cilia. Presented as the proportion of cells determined by DAPI-positive nuclei possessing pericentrin and acetylated tubulin-positive primary cilia. (Error bars ±SEM, three independent experiments, ≥350 cells per experiment, *p* < 0.05). (**C**) The mean area of pericentrin-positive pericentriolar material in immunostained images. Area in *Ta3^−/−^* iPS (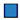), *Ta3^−/−^* ES cells (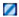), R1 (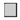), *Ta3^fl/fl^* iPS (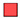), *Ta3^fl/fl^* ES cells (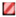). (Error bars ±SEM, three independent experiments, ≥350 cells per experiment, * *p* < 0.05, one-way ANOVA with Tukey’s post-hoc). (**D**) Primary cilia in R1, transgene-free *Ta3^fl/fl^* X12 or X22 and *Ta3^−/−^* X10 and X17. ES cells immunostained for pericentriolar material 1 (Pcm1, hollow arrows) and acetylated tubulin (white arrows). Scale bar: 10 µm. (**E**) The mean area of pericentriolar material protein Pcm1 in immunostained images. Pcm1 areas in *Ta3^−/−^* iPS (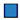), *Ta3^−/−^* ES cells (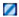), R1 (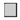), *Ta3^fl/fl^* iPS (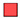), *Ta3^fl/fl^* ES cells (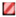). (Error bars ±SEM, three independent experiments, ≥350 cells per experiment, * *p* < 0.05, data were compared by one-way ANOVA with Tukey’s post-hoc). Scale bar: 10 µm.

The *Ta3* ES and integration-free *Ta3* iPS cell lines were stained for acetylated tubulin and pericentrin to identify the axoneme and pericentriolar material at the base of primary cilia, respectively (Figure 3A) and cell nuclei identified by staining with DAPI. The percentage of cells with primary cilia was quantified per DAPI-stained nuclei. Primary cilia were present in serum-starved R1 mESC, transgene-free *Ta3^fl/fl^* X12, X22 and *Ta3^fl/fl^* ES9 cells with no significant differences between these cell lines but were absent in the *Ta3^−/−^* ES6, *Ta3^−/−^X10* and *X17* iPS cell lines (*p* < 0.05; Figure 3B). Consistently, the area occupied by the centrosome and pericentriolar material component pericentrin in the *Ta3^−/−^* iPS and ES cells was at least a third larger (*p* < 0.05; Figure 3C) as compared to those in R1 and *Ta3^fl/fl^* ES and iPS cells and appeared less discrete (Figure 3A). Increased filamentous acetylated tubulin staining was also seen surrounding the pericentrin-stained base of the primary cilia (Figure 3A,D).

We analysed the distribution of the centriolar satellite protein pericentriolar material 1 (PCM1) and found that its domain was also expanded in the *Ta3^−/−^* ES and iPS cell lines (Figure 3D). The mean area of PCM1 positive material in *Ta3^−/−^* ES 6, iPS X10 and X17 cell lines was significantly increased when compared to *Ta3 ^fl/fl^* ES 9, *Ta3 ^fl/fl^* iPS X12 and X22 and R1 ES cells (*p* < 0.05, Figure 3E). Increased PCM1 density was also reported by Kobayashi et al. (2014) [28], upon siRNA knockdown of the human Talpid3 ortholog in the RPE1 cell line.

### 2.7. Embryoid Bodies Lacking Ta3 Are Still Competent to Form All Primary Embryonic Germ Layer Derivatives

As we observed an effect of deletion in *Ta3* on primary cilia formation in the *Ta3*^−/−^ ES and iPS cell lines, we investigated if these cells retained their ability to form derivatives of the three primary embryonic germ layers. We found that *Ta3^fl/fl^* and *Ta3^−/−^* ES, as well as iPS cells, gave rise to cell types derived from the three primary embryonic germ layers as seen by the expression of *Otx1*, *Sax1* and *Sox1* (ectoderm); Vimentin (endo/mesoderm), Cytokeratin 18 and 19 (ecto/endoderm); *Msx1/2*, Myosin Heavy Chain and Myogenin, *Bmp2/4*, *Flk1*, *Flt1* and *VEGF* (mesodermal lineages) (Figure S4A–C).

### 2.8. Deletion in Ta3 Disrupts Extra-Embryonic Endoderm Differentiation and Polarisation in Embryoid Bodies

ES and iPS cells can be induced to form embryoid bodies (EBs) when cultured in suspension and provide a model to study early differentiation cues [39,42]. Initially, EBs have an outer primitive endoderm (PrE) layer, which further differentiates into the parietal (PaE) and the visceral endoderm (VE). This recapitulates the extra-embryonic endoderm formation of preimplantation mouse embryos [43,44,45].

**Figure 4:**
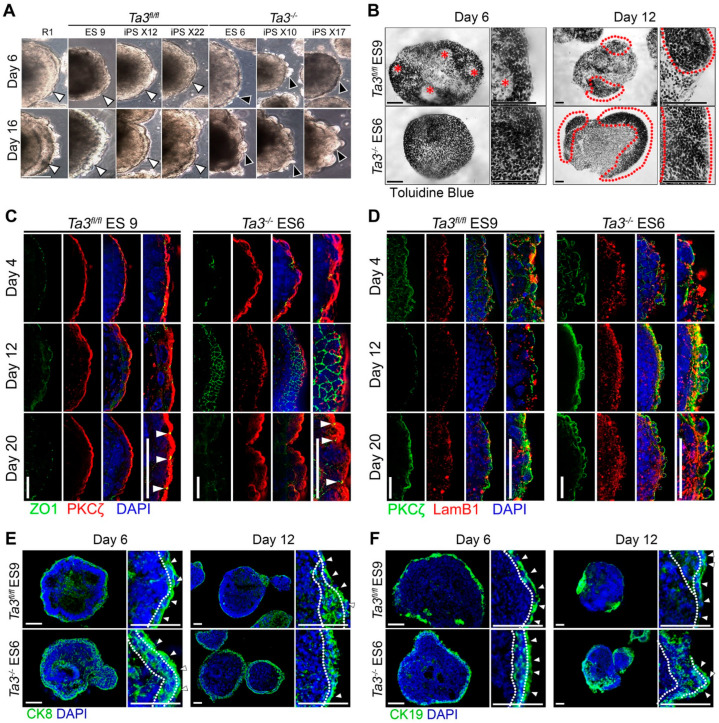
Differentiation of *Ta3* ES and transgene-free iPS cells to EBs and distribution of cell polarity, VE and PaE endodermal markers. Representative immuno- and histochemically stained cryosections of *Ta3^fl/fl^* and *Ta3^−/−^* ES-derived embryoid bodies at the indicated time points of three independent experiments. (**A**) Phase-contrast images of R1, *Ta3^fl/fl^* 9, *Ta3^−/−^* 6 ESC and transgene-free *Ta3^fl/fl^* (X12 and X22) or *Ta3^−/−^* (X10 and X17) iPS cell derived EBs. Scale bars: 200 µm. (**B**) Toluidine blue, dotted lines demarcate densely stained PaE-like cells. Scale bars: 50 µm. (**C**) Immunostaining for apical membrane marker PKCζ and tight junction marker ZO-1. Scale bars: 25 µM. (**D**) Immunostaining for apical membrane marker PKCζ and LamB1. Scale bars: 25 µM. (**E**) Immunostaining for CK8 counterstained with DAPI. Cropped insets and asterisks indicate PrE/VE outer single cell squamous epithelia and a dotted line demarks PaE from PrE/VE and EB interior. Scale bar: 50 µm. (**F**) Immunostaining for CK19 counterstained with DAPI. Cropped insets and asterisks indicate PrE/VE outer single cell squamous epithelia and a dotted line demarks PaE from PrE/VE and EB interior. Scale bar: 50 µm.

We generated EBs from both *Ta3^fl/fl^* and *Ta3^−/−^* transgene-free iPS and ES cells. Both *Ta3^fl/fl^* and *Ta3^−/−^* ES and iPS cell lines formed small loosely packed aggregates by day two. Differences between *Ta3^fl/fl^* and *Ta3^−/−^* EBs began to be apparent by day six, where *Ta3^fl/fl^* EBs have compacted centres and smooth outer layers (Figure 4A, white arrows) while the outer layer of *Ta3^−/−^* EBs remain rough and thickens in comparison (Figure 4A, black arrows). Toluidine blue stained sections at the same time points showed a distinct homogeneity of densely packed cells in *Ta3^−/−^* ES6 EBs while *Ta3^fl/fl^* ES9 EBs contained multiple rosette-like structures with varying densities of cells (Figure 4B, asterisks). A single layer of flattened epithelial cells comprising the primitive endoderm (PrE) was seen lining the outer surface of the EBs in both cases.

Between days ten and sixteen, *Ta3^−/−^* ES6 EBs became considerably more disorganised (Figure 4A, black arrows). By day twelve, the areas of dense patches of toluidine blue stained PaE-like tissue in *Ta3^fl/fl^* EBs were reduced, and the defined outer epithelial layer was no longer detectable (Figure 4B). In contrast, on day twelve, *Ta3^−/−^* EBs retained extensive stretches of PaE-like cells forming a thick multicellular layer around the outside of the EB, and these persisted until day eighteen. We did not observe any difference in the overall size between *Ta3^fl/fl^* and *Ta3^−/−^* EBs.

These changes in extra-embryonic structures are also reflected in the polarity of the outer layer of the EBs, here shown by the distribution of polarity markers, such as zona occludens-1 (ZO-1), protein kinase C type zeta (PKCζ), and Laminin B1 (LAMB1). In both *Ta3^fl/fl^* and the *Ta3^−/−^* EBs, ZO-1 is present by day four, but tight junctions have not formed, while PKCζ and LamB1 are present but discontinuously in the outer cell layer (Figure 4C,D). ZO-1 is organised into tight junctions by day twelve in the outer layer of only the *Ta3^fl/fl^* EBs and is not discernable in *Ta3^−/−^* EBs. Instead, in *Ta3^−/−^* EBs, a uniform distribution of ZO-1 was observed extending 3–4 cells deep from the surface (Figure 4C). PKCζ and LamB1 expression became strongly restricted to a one-cell thick outer epithelium by day twelve in both *Ta3^fl/fl^* and *Ta3^−/−^* EBs. This persisted until day twenty in *Ta3^fl/fl^* EBs while in *Ta3^−/−^* EBs, PKCζ and LamB1 expression was expanded to an outer 3–4 cell thick layer (Figure 4D).

Together, these morphological differences suggest that in the *Ta3^−/−^* ES and iPS EBs, the PaE layer becomes multilayered at the expense of the VE, which either does not arise or degenerates after formation.

### 2.9. Deletion in Ta3 Alters Extra-Embryonic Endoderm Specification and Organisation

To confirm these morphological observations are a result of defects in extra-embryonic endoderm differentiation, we investigated the basis for these differences by staining for Cytokeratin-8 (CK8), a commonly used marker for PrE; Cytokeratin 19 (CK19) and Laminin B1, markers for PaE; AFP, a marker for the VE layer and some other types of fetal endoderm.

CK8 immunostaining confirmed the identity of the outer epithelial layer as PrE, which on day six appears as a distinct single-cell squamous epithelium in *Ta3^fl/fl^ EBs* (Figure 4E, asterisks), unlike in the *Ta3^−/−^* EBs where an additional layer of CK8 positive columnar epithelium was present (Figure 4E, dotted line). CK19 immunostaining was initially observed in the PrE in EBs of both genotypes. However, its expression by day twelve was greatly reduced in areas with PrE/VE morphology but was high in cells with PaE morphology (Figure 4F).

In day twelve *Ta3^−/−^* EBs the CK8 positive layer was disorganised and greatly expanded forming an extensive multicellular layer across the surface of the EB, unlike that in *Ta3^fl/fl^* EBs where only small pockets of comparable organisation were observed (Figure 4E). CK19 was greatly reduced in areas with PrE/VE morphology by day twelve but was high in cells with PaE morphology (Figure 4F). As with CK8, both CK19 positive PaE and adjacent PrE/VE cells were identifiable in *Ta3^fl/fl^* EBs (Figure 4F, dotted lines and asterisks, respectively). However, in the *Ta3^−/−^* EBs, fewer cells were positive for CK19; staining was less contiguous in PrE/VE, while an expanded domain of CK19 staining was seen in areas with a PaE morphology.

In *Ta3^−/−^* EBs, CK8 was expression localised in large groups of cells which ballooned out of the EB and a few single cells in the interior, while CK19 extended into the interior overlapping with the extensive densely packed regions of nuclei which correspond to the dense toluidine blue staining in Figure 4B.

### 2.10. Primitive Endoderm Cells in Ta3^fl/fl^ ES Cell-Derived EBs Possess Primary Cilia Which Are Absent in Ta3^−/−^ EBs

**Figure 5:**
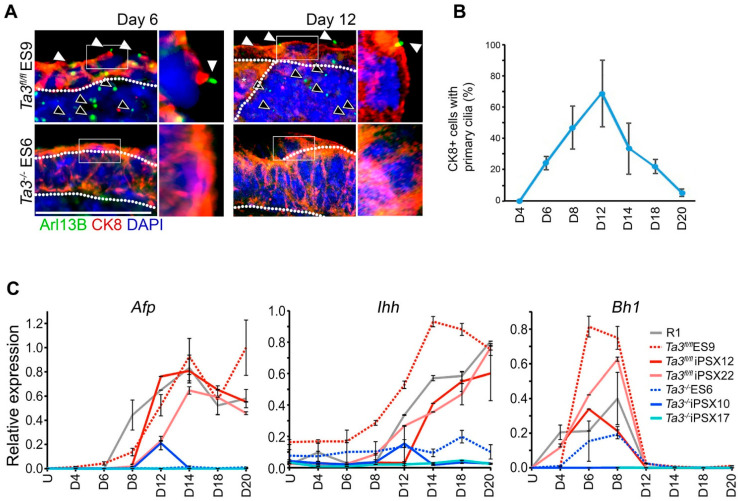
Expression of differentiation markers in *Ta3* ES and transgene-free iPS cells EBs. (**A**) Immunostaining for CK8 and Arl13b was counterstained with DAPI. Dotted line demarks PaE from PrE/VE and EB interior. Single cell highlighted by white boxed area and shown enlarged on right of each panel. White arrows indicate cilia present in the CK8 positive epithelia, and black arrows indicate cilia in the EB interior. Scale bar: 50 µm. (**B**) Quantification of primary cilia frequency on CK8+ cells in *Ta3^fl/fl^* EBs across the differentiation time course. Data from five EBs, each from three independent experiments. Error bars St. Dev. No cilia were observed in *Ta3^−/−^* EBs at any stage. (**C**) qRT-PCR for expression of visceral endoderm marker AFP, primitive endoderm marker Ihh and primitive hematopoiesis marker Bh1. R1 (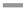), *Ta3^fl/fl^* ES 9(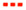), *Ta3^fl/fl^* iPS X12 (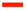), *Ta3^fl/fl^* iPS X22 (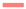), *Ta3^−/−^* ES 6 (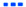), *Ta3^−/−^* iPS X10 (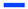) and *Ta3^−/−^* iPS 17 (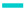). n = 3. Error bars ± SEM. qPCRs were performed in triplicate for each time point. Data from 3 independent experiments.

In order to see which cells within the embryoid bodies should be responsive to primary cilia-mediated signalling, we stained the EBs of both genotypes with antibodies to Arl13b and co-stained with antibodies to CK8 to identify the extra-embryonic endoderm cells. (Figure 5A). The frequency of CK8-positive cells with primary cilia was quantified across the time course (Figure 5B). In *Ta3^fl/fl^* EBs, primary cilia were seen on day six in 24.1% of CK8+ cells. Their frequency on CK8+ cells continued to increase to days eight and twelve, up to a maximum of 68.2%, coinciding with the most extensive CK8 distribution around the outer edge of the EB (Figure 5B). It is possible that our quantification of primary cilia in CK8+ cells may miscount cells or cilia at the boundaries of sections; however, the change in frequency of primary cilia in WT EBs during the time course is robust while, in contrast, *Ta3^−/−^* EBs were devoid of primary cilia at all time points (Figure 5A).

### 2.11. Deletion in Ta3 Favours Differentiation to a PaE over VE Lineage

Ihh signalling is implicated in lineage allocation during the formation of the extraembryonic endoderm [10]. As this is likely one of the developmentally earliest signalling pathways dependent on primary cilia for transduction, we sought to characterise the effects of the deletion in *Ta3* and the absence of primary cilia on the spatiotemporal expression of Ihh and the specification of PaE and VE lineages.

Expression of the VE marker Afp was first detected in *Ta3^fl/fl^* EBs on day six in the outer layer of the EB (Figure S5A). A gradual increase in the numbers of Afp-positive cells and level of Afp expression was seen from day eight to day twenty. In contrast, the onset of Afp expression was delayed in the *Ta3^−/−^* EBs. Weak expression of Afp was observed on day twelve with no further increase (Figure 5C and Figure S4A). qRT-PCR confirmed the temporal expression pattern of Afp immunostaining. In R1, *Ta3^fl/fl^* iPS and ES EBs (Figure 5C), *Afp* transcripts were first detected on day eight, peaked between days twelve and fourteen, and expression declined by day twenty. No sustained Afp expression was seen in the *Ta3^−/−^* EBs (Figure 5C and Figure S4A).

### 2.12. Deletion in Ta3 Disrupts Expression and Processing of Hh Pathway Components During EB Differentiation

Hh expression was analysed by immunohistochemistry using the 5E1 antibody (T. Jessel, DSHB), which recognises both Shh and Ihh and by qRT-PCR for both *Shh* and *Ihh* transcripts separately. Hh protein expression was detected in the *Ta3^fl/fl^* EBs first on day eight (Figure S5B), increased between days twelve and fourteen, and subsequently decreased in the outer layers except in a small number of cells in the interior of the EBs. In contrast, Hh expression was undetectable in the outer layer of the *Ta3^−/−^* EBs, and this possibly underlies the lack of VE indicated by the absence of Afp in *Ta3^−/−^* EBs (Figure S5A).

Low-level expression of Hh can be seen between days four and six in EBs of both genotypes; however, the onset of robust *Ihh* expression in *Ta3^fl/fl^* EBs occurred subsequent to the onset of Afp expression on day 8 in line with the specification of VE, peaking on day fourteen (Figure 5C and Figure S5B). In *Ta3^−/−^* EBs, low-level expression of *Ihh* is seen transiently only at earlier time points by qRT-PCR. Expression of *Ihh* steadily increased in R1 ES, *Ta3^fl/fl^* ES and *Ta3^fl/fl^* iPS cell-derived EBs (Figure 5C), but the levels of *Ihh* in the *Ta3^−/−^* iPS and ES cell-derived EBs remained very low (Figure 5C). Ihh, secreted by the VE, plays a role in primitive erythropoiesis [10,46] and embryonic haemoglobin Z (Bh1) is produced by primitive haematopoietic cells [47,48]. In R1, *Ta3^fl/fl^* ES, and iPS EBs, *Bh1* mRNA expression increased between days four and nine. Expression levels of *Bh1* were dramatically reduced in *Ta3^−/−^* ES EBs and undetectable in the *Ta3^−/−^* iPS EBs (Figure 5C). The reduced expression of *Bh1* suggests an indirect effect on fetal haematopoiesis, possibly due to abnormal yolk sac differentiation in the absence of VE in *Ta3^−/−^* iPS EBs.

**Figure 6:**
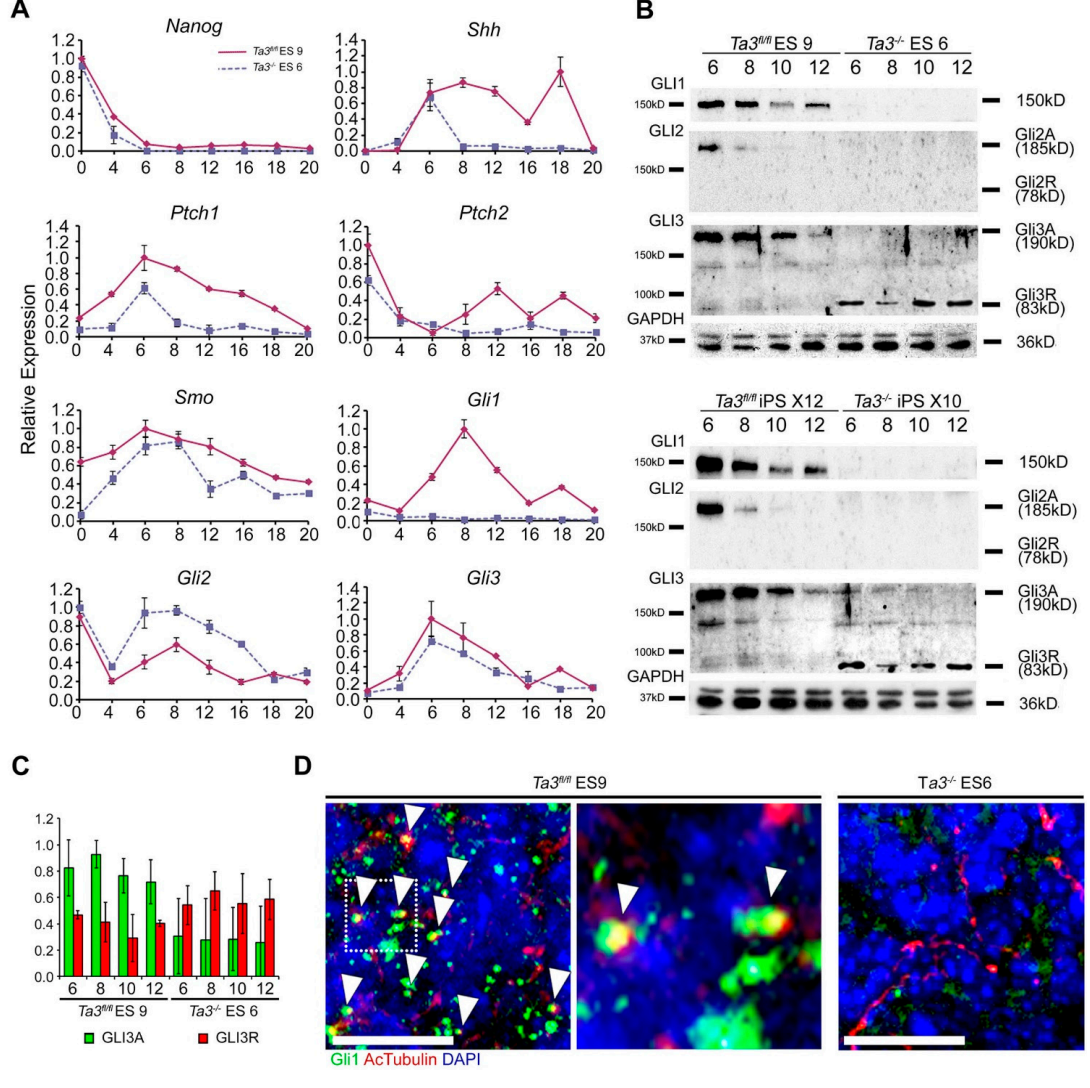
Expression of Shh pathway components and processing of Gli transcription factors. (**A**). qRT-PCR for pluripotency marker Nanog and Hedgehog pathway components Shh, Ptch1/2, Smo, Gli1, Gli2 and Gli3 in *Ta3^fl/fl^* ES 9 and *Ta3^−/−^* ES 6 EBs. *n* = 3. Error bars: ±SEM. qPCRs were performed in triplicate for each time point. Data from 3 independent experiments. (**B**) Immunoblots of EB lysates for Gli expression and processing during differentiation. GAPDH-loading control. (**C**) Quantification of the relative expression levels of Gli3A (190 kDa) and Gli3R (83 kDa) in *Ta3^fl/fl^* and *Ta3^−/−^* EBs relative to GAPDH. Quantified from three independent experiments. Error bars ± SEM. (**D**) Expression of Gli1 in *Ta3^−/−^* and *Ta3^fl/fl^* EBs co-stained with acetylated tubulin. Co-localised Gli1 and acetylated tubulin highlighted by arrow heads. Scale bar: 25 µm.

Expression of *Shh* commenced between days four and six in *Ta3^fl/fl^* and in *Ta3^−/−^* EBs (Figure 6A) and was maintained in *Ta3 ^fl/fl^* EBs until day eighteen, after which it decreased. In contrast, *Shh* expression in *Ta3^−/−^* EBs dropped to barely detectable levels by day eight and continued to remain low (Figure 6A).

The expression of *Hh* transcripts correlated with the immunostaining. Since there was an abrogation of *Hh* expression and defects in differentiation in both early and late-stage *Ta3^−/−^* EBs, we further investigated the effects on the expression levels of components of the Hh signalling pathway as well as on the processing of the Gli transcription factors that are key transducers of Hh signalling.

*Gli1* was expressed at low levels in *Ta3^fl/fl^* EBs between day zero and day four, which increased from day six onwards, peaking at day eight and then downregulated. In comparison, *Gli1* expression was barely detectable in *Ta3^−/−^* EBs (Figure 6A). Undifferentiated *Ta3^−/−^* and *Ta3^fl/fl^* ES cells expressed high levels of *Gli2,* which was downregulated during the early stages of *Ta3^fl/fl^* EB formation until day four, after which time *Gli2* expression increased, reaching a peak at day eight (Figure 6A). Expression in *Ta3^−/−^* EBs peaked on day 6 at higher levels than in *Ta3^fl/fl^* EBs and then declined. Overall, the expression level of *Gli3* was similar in both *Ta3^−/−^* and *Ta3^fl/fl^* EBs with a substantial peak of expression on day six, gradually declining at similar rates, suggesting any changes in GLI3 levels are occurring during GLI3 processing rather than at the transcript level (Figure 6A).

The expression of *Ptch1* and *Smo* mimic the patterns seen for the *Gli* transcripts with a peak during EB differentiation between days six and eight, and the expression of both is reduced in *Ta3^−/−^* EBs. *Ptch2* expression decreased rapidly upon differentiation with only a minor fluctuating increase in expression seen at later time points for *Ta3^fl/fl^* EBs only (Figure 6A)

The loss of *Nanog* expression was used as further confirmation that loss of pluripotency was occurring with similar efficiency between the two genotypes. Robust downregulation was seen by day four, with levels barely detectable by day six in both *Ta3^−/−^* and *Ta3^fl/fl^* EBs (Figure 6A).

### 2.13. Effect of Deletion in Talpid3 on the Processing of the Gli Transcription Factors

In addition to modulating the expression of Gli transcription factors, Hh signalling through the primary cilia also results in the stabilisation of the Gli proteins, preventing degradation (Gli1) or proteolytic processing to repressor forms (Gli2/3) [49,50,51].

We assessed the consequences of loss of *Ta3* and the absence of primary cilia on protein levels and processing of the Gli transcription factors during EB differentiation. Gli1 was present between days six and twelve in *Ta3^fl/fl^* ES9 and iPS X12-derived EBs, with levels peaking between days six and eight. In comparison, Gli1 in the *Ta3^−/−^* ES and iPS EBs was barely detectable between days six and twelve (Figure 6B), as also seen by qRT-PCR (Figure 6A). Unprocessed Gli2A levels showed a reduction between days six and twelve in *Ta3^fl/fl^* EBs. Like Gli1, Gli2A could not be detected in *Ta3^−/−^* ES and iPS EBs, suggesting that while there is a reduction in Gli2 transcription (Figure 6A), there may also be reduced translation or rapid degradation of the Gli2 protein. No Gli2R could be detected in either *Ta3^fl/fl^* or *Ta3^−/−^* EBs (Figure 6B). Both Gli3A and Gli3R were present in the *Ta3^fl/fl^* EBs at all time points but with a higher proportion of Gli3A, with levels of both decreasing over the time course (Figure 6B,C). In contrast, total Gli3 levels in *Ta3^−/−^* EBs were reduced with an increase in Gli3R over Gli3A, which was maintained over the time course (Figure 6C). Co-immunostaining for Gli1 and acetylated tubulin showed frequent colocalisation in *Ta3^fl/fl^* EBs, while no Gli1 staining or cilia are seen in *Ta3^−/−^* EBs, confirming the Western blot data (Figure 6D).

### 2.14. Talpid3 Is Required for Efficient Neuroectodermal and Motor Neuron Differentiation

Shh signalling plays a key role in the ventralisation of the spinal cord and motor neuron differentiation [12,13,14,52]. *Ta3^−/−^* mice show abnormal motor neuron differentiation in the spinal cord [25,53]. The directed differentiation of pluripotent cells to a motor neuron (MN) lineage in vitro is well established, and MN frequency can be increased by the use of Hh agonists such as purmophamine [54]. Defective retinoid-induced differentiation has been previously noted in the absence of further ciliary proteins Meckelin and Jouberin [55]. The effect of the deletion in *Ta3* on the ability of the *Ta3^−/−^* ES and iPS to differentiate to the neuronal lineage and further to motor neurons was assessed using this approach. *Ta3^fl/fl^* or *Ta3^−/−^* ES cells and *Ta3^fl/fl^* or *Ta3^−/−^* transgene-free iPS cell lines were differentiated in a retinoic acid-containing medium and plated onto Matrigel with or without purmorphamine. Differentiated cells were stained with either nestin (Nes) for neural precursors, neurofilament (NF) for mature neurons or a combination of islet1 (Isl1) and peripherin (Per) for mature motor neurons.

**Figure 7:**
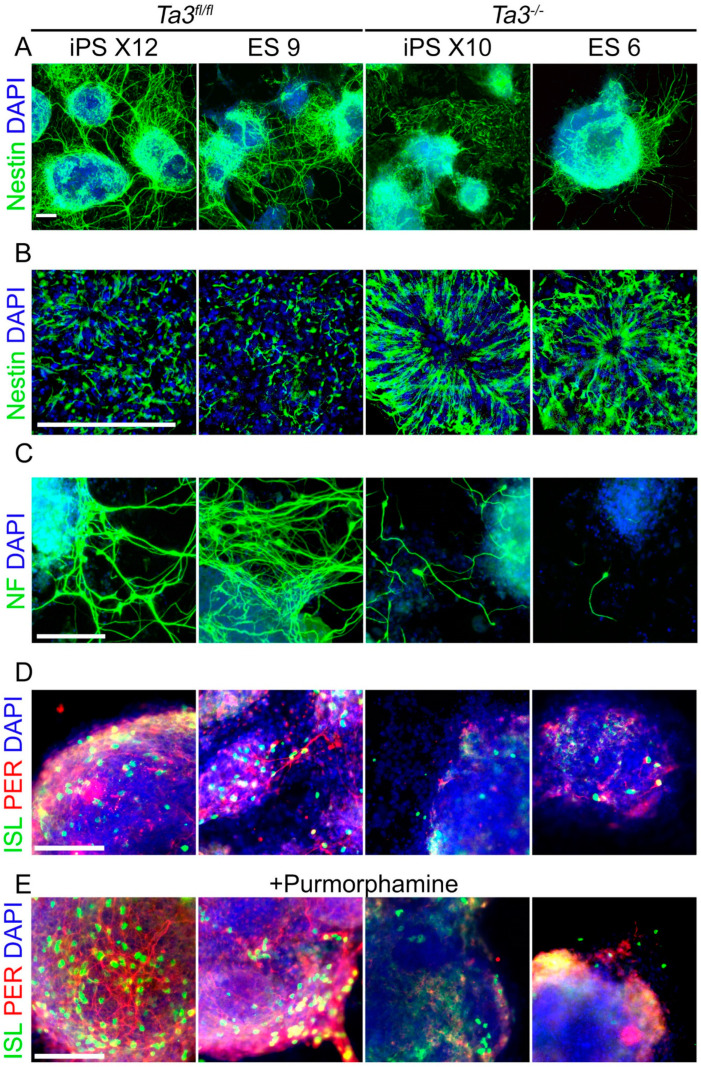
Neuronal Differentiation of EBs on Matrigel. Embryoid bodies were formed from *Ta3^−/−^* and *Ta3^fl/fl^* ES and iPS cells and differentiated on Matrigel with RA. (**A**) Immunostaining shows nestin-positive precursors on day six and nestin-positive immature neurites extending from differentiating colonies. (**B**) Higher magnification of colonies shows that in comparison to *Ta3^fl/fl^*, nestin-positive cells in *Ta3^−/−^* cultures remain in a more immature state. *Ta3^−/−^* nestin-positive neuroepithelial cells maintain a more rosette-like morphology, while *Ta3^fl/fl^* cells have acquired a neuroblast-like morphology and are migrating outwards. Staining for neurofilament (NF) identifies mature neurons (**C**), while Islet (Isl1/2l) and Peripherin (Per) identify post-mitotic motor neurons (**D**). The inclusion of the hedgehog agonist purmorphamine increases the frequency of motor neuron specification in *Ta3^fl/fl^* differentiations but fails to rescue *Ta3^−/−^* cultures (**E**). Scale Bar: 100 µm. Representative images from three independent experiments.

Nestin expression in *Ta3^fl/fl^* ES and iPS cells was first detected on day four of differentiation but to a considerably lesser extent in *Ta3^−/−^* ES and iPS cells. On day six, the differentiating *Ta3^fl/fl^* cultures produced long nestin-positive neurites (Figure 7 and Figure S6A). In comparison, the differentiating *Ta3^−/−^* cultures had fewer and less organised nestin-positive neurites. Small rosettes of neural progenitors were still seen in the centre of many of the *Ta3^−/−^* EBs, unlike in the *Ta3^fl/fl^* EBs.

*Ta3^fl/fl^* EBs produced a significant number of NF-positive neurons with long neurites, which formed extensive networks between adjacent EBs by day six of differentiation. In contrast, NF expression was delayed in *Ta3^−/−^* EBs, and fewer NF-positive neurons were present in the cultures (Figure 7 and Figure S6B).

Isl1 and Per co-expression identifies post-mitotic motor neurons (MNs). MNs were first detected in small numbers on day four in the differentiating *Ta3^fl/fl^* cultures and increased by day six. Very few MNs were identified in *Ta3^−/−^* cultures (Figure 7 and Figure S6C). The inclusion of purmorphamine during differentiation resulted in a substantial increase in MN numbers in *Ta3^fl/fl^* cultures, but the *Ta3^−^*^/−^ cultures were non-responsive (Figure 7 and Figure S6D).

To determine if the inability of *Ta3^−/−^* cells to undergo efficient motor neuron differentiation was an autonomous defect, we differentiated composite EBs composed of either *Ta3^−/−^* or *Ta3^fl/fl^* iPS cells with control ES cells. If *Ta3^−/−^* iPS cells can differentiate into motor neurons during co-differentiation with the control ES cells, it would indicate a non-cell autonomous defect.

The composite EBs were generated by aggregating equal numbers of control ES cells expressing GFP-tagged Tau (TauGFP [56]) with either R1 control ES cells or *Ta3^−/−^* or *Ta3^fl/fl^* iPS cells expressing the CagTag reporter [57] (Figure S7B). The CagTag reporter expresses GFP-tagged histone2b and myristoylated tdTomato targeted to the membrane [57] allowing us to distinguish the control ES cell-derived TauGFP positive neurites from the tdTomato positive neurites derived from the control R1 ESCs or *Ta3^−/−^* or *Ta3^fl/fl^* iPS cells.

TauGFP-positive neurons readily differentiated from all three types of composite EBs. Similarly, tdTomato positive neurite outgrowths derived from ‘CagTagged’ R1 ESCs and *Ta3^fl/fl^* X12 iPSCs were also readily seen. In contrast, no *Ta3^−^*^/−^ X10 iPSC-derived neurites were observed in the case of TauGFP: *Ta3^−^*^/−^ X10 CagTag composite EBs, suggesting an intrinsic defect in *Ta3^−^*^/−^ cells (Figure S7C).

## 3. Discussion

Mouse and human ESCs possess primary cilia, which contain the working machinery for the well-characterised Hh signalling pathway [37]. However, it is unclear what roles *Ta3* or primary cilia play in ESCs; are they essential for maintaining stemness and self-renewal or for responding to differentiation cues? It is also of interest to know if somatic cells from mutant mice which lack primary cilia can be efficiently reprogrammed.

We have successfully reprogrammed *Ta3^−/−^* mouse fibroblasts lacking primary cilia to transgene-free iPS cells. These iPS cells are similar to the reference R1 ES cell line with regard to their morphology, growth properties, cell cycle and the ES cell molecular signature [58] but exhibit an altered response to differentiation cues affecting early extra-embryonic endodermal lineage allocation as well as motor neuron differentiation. These defects in lineage-specific differentiation of *Ta3^−/−^* iPS cells are also exhibited by the *Ta3^−/−^* ES cells, confirming that it is not an artefact of reprogramming.

The *Ta3^−/−^* ES and iPS EBs lacking primary cilia are devoid of VE. It has been reported that the outer layer of the EB produces *Ihh* [59,60]. *Ihh* signalling to the underlying layer, in turn, induces VE differentiation in the layer above and ectoderm specification in the core of the EB [10,11]. In the peri-implantation mouse embryo, this response to Ihh is mediated through BMP signalling [61].

Pluripotent embryonal carcinoma (EC) cell lines such as F9 have been used to study early differentiation events in vitro [62] prior to the development of ES cell-derived models. Maye et al. [10,11] have shown that overexpression of *Ihh* pushes F9 EC cells to the VE and PaE lineages without additional inducers, while abrogation of Ihh signalling leads to PaE differentiation at the expense of VE. Extraembryonic endoderm stem cells (XEN cells) favour a VE phenotype over PaE when treated with Ihh signalling response factors such as BMPs [63]. Interestingly, SMAD4/DPC4 mutant mouse embryos show defects in VE development as a result of impaired BMP/TGF-β signal transduction [64]. The phenotype observed in *Ta3^−/−^* EBs and the loss of Hh signalling components suggest a similar underlying primary cilia-dependent mechanism. Deletion in *Ta3* and loss of cilia disrupt Ihh and BMP signalling between the outer PrE and inner mesodermal tissues of the EBs, leading to increased PaE at the expense of VE. Insensitivity to Hh signalling can be seen in the altered Gli family processing patterns observed where Hh signalling should be active. Gli1, Gli2 & Gli3 protein levels were all significantly reduced in the absence of *Ta3*, with a significant switch in Gli3 processing to favour the repressor fragment. We also found Gli1 expression at the transcript level to be dependent on Hh signalling, as previously reported [65], and both Gli2 and Gli3 are affected primarily at the protein level. This defect appears to be cell autonomous as differentiation is not rescued in composite EBs where *Ta3* mutant cells would be subject to Hh signalling from WT cells.

Ref. Bangs et al. [66] did not detect primary cilia in the VE and trophectoderm of mouse embryos or in XEN cells and suggested that this is necessary so that cells of extraembryonic origin are unable to respond to Hh signals. In contrast, Wang et al. [67] report the presence of primary cilia on human trophoblast cell lines and show that they are required during placentation. The observations by Wang et al. [65], taken together with our findings, showing the aberrant specification of PaE and VE from the PrE in the *Ta3^−/−^* EBs, suggest that *Ta3* is required at least transiently for primary cilia-dependent Hh signalling during lineage allocation in the extraembryonic endoderm. The defects that we see in VE differentiation in *Ta3^−/−^* EBs may explain the embryonic lethality seen at E9.5 when *Ta3* is constitutively deleted [25], which could be partly due to defects in extraembryonic membranes. This is supported by the observations by Wang et al. [67], that invasion of the uterus by trophoblast cells requires primary cilia, which is prevented by depleting IFT88, leading to defective ciliogenesis.

Our results suggest at least three potentially overlapping mechanisms. Firstly, the absence of cilia in the underlying presumptive mesoderm may disrupt the feedback that drives further VE specification due to insensitivity to Ihh signalling. Secondly, the absence of cilia in the PrE may favour the specification of PaE over VE due to insensitivity to the feedback from the presumptive mesoderm. In addition, earlier fate choices made before or during PaE specification may limit the capacity for VE differentiation prior to the onset of Ihh signalling. Bangs et al. noted constitutive activation of the AURKA pathway prevented primary cilia formation in their models, a driver of cilia disassembly, and the status of this pathway in the equivalent cell types in EBs is unknown. Further experiments need to be performed to resolve these findings and to reconcile our results and those of Maye et al. (2000, 2004) [10,11] with the findings of Bangs et al. (2015) [66].

In the mouse embryo, the VE plays an instructive role during primitive erythropoiesis [47,68]. This is promoted, at least in part, by Ihh, produced by the VE [10,46]. Since the VE is missing or abnormal in *Ta3^−/−^* EBs, it may impact on yolk sac erythropoiesis [47,48].

*Ta3^−/−^* iPS and ES cells respond to RA and form neural progenitors but exhibit defective MN differentiation. ES cells lacking two other ciliopathy-associated proteins, Mecklin and Jouberin, also show aberrant neuronal differentiation, which has been attributed to their inability to respond to RA [55]. It is clear there is also a defect in the specification of neuronal identity here beyond purely motor neuron specification; further investigation of global and motor neuron-specific progenitor proportions at key stages will allow dissection of the Shh signalling defect specifically. Our findings are significantly different from those of Hunkapiller et al. [38], who report that *Ofd1^−/−^* ES cells, which lack primary cilia, have an increased propensity for neuronal differentiation but fail to form V3 interneurons. Despite both *Ta3* and *Ofd1* mutants lacking primary cilia, the functionality and composition of the basal body structures or other cilia-independent roles could account for these differences in phenotype. Yin et al. [24] showed that the ciliary vesicle associated with the basal body failed to dock with the apical membrane in *Ta3* mutant cells, and Kobayashi et al. [28] showed that *Ta3* localises to the distal tip of the mother centriole as part of the CP110-containing protein complex. In contrast, Ofd1 protein localises to the distal tips of both centrioles, suggesting a different function to *Ta3* [26]. It is also unclear at which point ciliogenesis is disrupted in Ofd1 mutants. It is likely that the differences in primary cilia defect between *Ta3* and *Ofd1* mutants and/or other cilia-independent roles could also account for the differences between these and other ciliopathy-associated genes.

The *Ta3^−/−^* ES and iPS cell lines we have generated provide an excellent cell culture model to explore further the molecular mechanisms underlying the function of *Ta3* both in early and late differentiation events. In light of the recent association of *Ta3* mutations with Joubert syndrome and Jeune asphyxiating thoracic dystrophy, these cell lines will be useful in investigating the molecular mechanisms underlying these disorders.

Taken together our data suggest that primary cilia are not important for maintaining the stem cell state but are necessary for keeping the ES cells poised to respond to differentiation signals.

## 4. Methods

### 4.1. Knockout and Transgenic Mice

Talpid3 constitutive knockout mice (*Ta3^−/−^*) were maintained as a heterozygous line, and Talpid3 conditional (*Ta3^fl/fl^*) mice were maintained as a homozygous line on a *C57/Bl6* background on a 12 h light/dark cycle with access to food and water ad libitum. Mice were genotyped as described earlier [25]. The floxed allele was identified by PCR using the primer pair 1647_31/1647_32, which produces 351 bp and 470 bp products from the wild-type and *Ta3^fl/fl^* alleles. The *Ta3* allele with the exon11–12 deletion was identified using the primer pair 1647_31/1738_5, which produced a 273 bp product. An internal control PCR was performed using the primer pair 1260_1/1260_2. For PCR conditions and sequences, see Supplementary Table S2.

### 4.2. Animal Use Ethics

All animal procedures were reviewed and approved by the Animal Welfare & Ethical Review Body of the University of Bath and performed under approved Project and Personal licences in accordance with UK Home Office guidelines and the UK Animals (Scientific Procedures) Act, 1986.

### 4.3. Cell Culture

*Ta3^−/−^* fibroblasts were isolated from 12.5 dpc embryonic limbs of *Ta3* conditional knockout mice in which PrxCre was activated in the limbs, as described in Bangs et al. (2011) [25]. Wild-type fibroblasts were established from 13.5 dpc CD1 mouse embryos, *Ta3^fl/fl^* embryos and limbs of 13.5 dpc *Talpid^fl/fl^* mouse embryos. Fibroblasts were cultured in DMEM (Gibco, Waltham, MA, USA) containing Glutamax (Gibco), NEAA (Gibco) and 10%FCS. The mouse ES cell line R1 was routinely maintained on a feeder layer of Mitomycin C treated fibroblasts in mES medium (DMEM with Glutamax, 10% Foetal bovine serum (FBS), 5% Knockout Serum Replacement (KOSR, GIBCO), 1% Non-essential amino acids (NEAA) and 0.1 µM 2-Mercaptoethanol). The ES cell line, Tau-GFP (provided by J Nicholls, Cambridge, UK), was cultured in mES media containing LIF (1000 U/mL) and 2i [56]. R1 and TauGFP ES cell lines were adapted to feeder-free conditions and cultured in mES medium supplemented with LIF (1000 U/mL) and 2i. The PA6 stromal cell line was maintained in alpha MEM (Gibco) containing Glutamax, NEAA and 10% FCS.

### 4.4. Transfection and Reprogramming of MEFs Using the PiggyBac OSKML Vector

Mouse embryonic fibroblasts (MEFs) were plated onto six-well plates (10^5^ cells per well) one day prior to transfection. MEFs were transfected with 2 μg of *pCMV-mPBase34* and 2 µg of the *piggyBac-based OSKML* reprogramming plasmid using Fugene HD (Promega, Madison, WI, USA) (Fugene: DNA ratio of 3:1) according to the manufacturer’s instructions. Twenty-four hours after transfection, MEFs were trypsinised and plated on feeder layers at a split ratio of 1:18 in the MEF medium. The medium was changed to mES medium on day two and changed every 48 h. On day five, the medium was changed to a serum-free mES medium containing 15% KOSR instead of FBS. The medium was refreshed every other day. On days 10–14, colonies were counted, picked and further expanded. Two replicate experiments were performed in the case of the *Ta3^fl/fl^* fibroblasts and 13.5 dpc wild-type MEF and three experiments were conducted in the case of the *Ta3^−/−^* fibroblasts.

### 4.5. Isolation and Establishment of ES Cell Lines

ES cells were established from blastocysts of *Ta3^fl/fl^* and *Ta3^−/−^* mice. Blastocysts were isolated from 4-week-old females *Ta3^fl/fl^* (C57Bl6) and *Ta3^+/−^* (CD1) that had been induced to superovulate by an injection of 5 IU of pregnant mare serum gonadotropin, followed by a second injection of 5 IU of human chorionic gonadotropin (hCG) 48 h later and mated with *Ta3^fl/fl^* males and *Ta3^+/−^* males respectively. Blastocysts were plated individually into wells of a Nunc (Roskilde, Denmark) 4 well plate on Mitomycin C treated mitotically inactivated mouse embryonic fibroblasts (MitC MEFs) in DMEM supplemented with Glutamax, 10% FCS, 1% NEAA, 5% KOSR and 0.1 µM 2-Mercaptoethanol containing a two-inhibitor cocktail (2i medium [56]). ICM outgrowths were trypsinised on day 6 or 7 and plated on MitC MEFs in 6 well plates, further expanded and frozen at passage 3 or 4. ES cell lines were genotyped for the *Ta3^fl/fl^*, *Ta3^−/−^* and wild-type alleles [25].

### 4.6. Genotyping of iPS and ES Cells

Feeder-free cells on 24 well plates were trypsinised, resuspended in 200 µL medium, transferred to a PCR tube and centrifuged. The cell pellet was lysed in 100 µL of alkaline lysis buffer (25 mM Sodium Hydroxide, 0.2 mM EDTA). Samples were heated at 95 °C for 30 min, cooled to 4 °C and neutralised with 100 µL of 45 mM Tris-HCl, pH 5. Samples were mixed, and 1 µL was used to seed PCR reactions to determine the presence of the wild type, *Ta3^fl/fl^* or *Ta3^−/−^* allele [25] and for the presence of SRY sequence to identify male ES lines (for primer sequences and PCR conditions see Supplementary Table S2).

### 4.7. Determination of Copy Numbers of Integrated Reprogramming Vector by qPCR

DNA was isolated from iPS lines that were cultured for two passages in feeder-free conditions. Each cell line was lysed in Proteinase K lysis buffer (0.2 mg/mL Proteinase K, 0.5% SDS, 50 mM EDTA, 100 mM Tris HCl) overnight at 56 °C. DNA was isolated from the lysates by phenol: chloroform (1:1) extraction and precipitation of the aqueous phase using 0.1 M Lithium Chloride and 70% Ethanol. The precipitated DNA was resuspended in TE. Genomic DNA was checked on a 0.8% agarose-TBE gel to assess integrity.

DNA concentrations were estimated by UV spectrophotometer (Nanodrop 1000, Thermo Fisher, Waltham, MA, USA). The relative ratio of a vector-specific sequence to an endogenous sequence (*Klf-F2A* to *Cdx1*) was determined by qPCR. Reactions were seeded with ~100 ng DNA (see Supplementary Table S3 for primer sequences). A two-copy reference was created by ligation of the *Klf4-F2A* PCR fragment to the *Cdx1* PCR fragment. The full-length ligation product was amplified by PCR, gel-purified and spiked into DNA isolated from *Cdx1* knockout mouse embryonic fibroblasts.

### 4.8. Splinkerette PCR for Integration Site Analysis

The integration site of the reprogramming vector was determined by Splinkerette PCR [41]. Splinkerette oligos Spl-top and Spl-CG were annealed at 95 °C for 5 min, then cooled to RT in a 100 μL volume with 10 mM Tris-HCl (pH 7.4) and 5 mM MgCl_2_.

DNA from iPS clones was digested overnight by Msp I, Taq I or HinP1I (2 μg DNA with 20U enzyme in a 50 μL reaction volume). After heat inactivation, ligation was set up with 2 μL of the digest, T4 ligase (5U, Fermentas, Waltham, MA, USA) and annealed splinkerettes (11 pmol) and incubated overnight at 16 °C. One microliter of the ligation mixture was then subjected to nested PCR using either Spl and PB3 primers or Spl and PB5 primers (with P1 and P2 nested sets). See Supplementary Table S3 for primer details. Second-round reactions were resolved on 1% Agarose-TBE gels and bands excised, gel-purified and sequenced (MWG Eurofins). Insertion sites were identified from sequencing results by BLAST.

### 4.9. Excision of Reprogramming Vector

Feeder-independent miPS cells (10^7^) were electroporated with 30 µg of *mPB* transposase vector at 300 V and 250 µF (BioRad Gene Pulser, Hercules, CA, USA) and plated out on three gelatinised 10 cm dishes (BD Falcon, Franklin Lakes, NJ, USA). Media was changed every day, and each dish was further split 1:6 onto 10 cm dishes on day three (at around 80% confluency). ES cells were selected in media containing 0.25 µM Ganciclovir on day five after electroporation when the cultures were around 70% confluent. Selection media was changed every day over a period of six days. Colonies were large enough for picking from day seven after selection. Colonies were picked, trypsinised and expanded. Frozen stocks were prepared, and DNA was isolated from mirror plates. Clones were checked for the absence of the reprogramming vector by PCR for either KLF4-F2A or the Puromycin resistance gene in conjunction with PCR for an endogenous gene (See Table S2 for primers).

### 4.10. Cell Cycle Analysis

Cell cycle analysis of ES and iPS cells was carried out by FACS (modified from Savatier et al. [69]). ES and iPS cells were plated on gelatin-coated 10 cm dishes (BD Falcon) at a density of 3 × 10^4^ cells/cm^2^. Cells were refed 48 h after plating, and Bromodeoxyuridine (BrdU; Sigma, St. Louis, MA, USA) was added to the medium 1 h after refeeding to a final concentration of 50 µM and incubated for 30 min. Following BrdU incorporation, cells were trypsinised and resuspended in culture medium, centrifuged and washed twice in PBS. Cells were resuspended in 200 µL PBS and fixed in 2 mL ice-cold 70% Ethanol. Cells were rehydrated in PBS for 10 min, then centrifuged at 500× *g* for 5 min, resuspended in 2 M HCl (1 mL) and incubated at RT for 20 min, followed by the addition of 10 mL of PBT (PBS with 0.5% BSA and 0.5% Tween 20) and centrifuged. After two further washes with PBT, cell number was estimated, and 10^6^ cells of each cell line were immunostained by incubating in 100 µL of primary antibody against BrdU (1:100, G3G4, DSHB) and incubation at RT for 30 min. Cells were washed with PBT (3 times) followed by incubation (30 min) in 100 µL secondary antibody (goat anti-mouse Alexa 488, Life Technologies, Waltham, MA, USA). Cells were washed-free of unbound secondary antibody with 4 washes of PBT and incubated in RNaseA (Sigma, 100 µL of 1 mg/mL stock) for 30 min at RT. Samples were diluted to 500 µL, and propidium iodide (PI, Sigma) was added to a final concentration of 10 ug/uL.

BrdU labelled cells were analysed on a FACS Canto (Becton Dickinson, Franklin Lakes, NJ, USA). Single-cell populations were identified and gated by comparison of the PI signal width to the area for analysis of DNA content by PI-Area on a linear scale and BrdU incorporation by AF488-area fluorescence on a log scale. A minimum of 20,000 singlet events were analysed for each sample. Cell cycle phases were gated based on DNA content and BrdU incorporation, i.e., G0/1 cells are 1× PI, Low Alexa 488, S phase cells are 1–2× PI High Alexa 488, and G2 cells are 2× PI, Low Alexa 488. The results from three analyses are presented as mean proportions of each cell cycle phase.

### 4.11. Colony Morphology Analysis

ES and iPS cells were cultured feeder-free in LIF-containing medium and plated on six-well plates coated with gelatin at 2 × 10^4^ cells/cm^2^. Colony morphology was assessed from phase contrast images obtained at 60% confluence after two passages in this manner. Where indicated, 1 µM Cyclopamine (Sigma, St. Louis, MA, USA) was added 24 h after the first passage. To determine the smoothness of the colony edges, the actual perimeter of each colony was first measured, then smoothed with a radius of 20 µm and re-measured. The ratio of smoothed to actual perimeter was calculated as an indicator of roughness, where a ratio of 1 indicates a perfectly smooth perimeter while values above 1 indicate roughness. One hundred colonies of each cell line were measured from three independent experiments. Smoothed-actual perimeters compared by one-way ANOVA with Tukey’s post-hoc.

### 4.12. Generation of EBs and Directed Differentiation

EBs were generated from iPS and ES lines by plating 2.5 × 10^6^/mL of feeder-independent cells in 10 mL of EB medium (DMEM + Glutamax containing 5% KOSR, 5% FCS, NEAA and 0.1 µm β-Mercaptoethanol) in 10 cm bacteriological Petri dishes (Sterilin, Newport, UK). EBs were cultured over a period of 20 days with media changes every 48 h. EBs were harvested for RNA isolation and for immunostaining at indicated time points.

Neuronal differentiation was induced in ES, and iPS EBs were generated as above. Retinoic acid (0.5 µM) was added to the EBs twenty-four hours after plating. On day two, EBs were transferred to a differentiation medium (alpha-MEM containing Glutamax, 1% NEAA, 0.1% KOSR and 0.5 µM RA) and 1 µM Purmorphamine where required. The EBs were seeded either on Matrigel (BD) coated coverslips (100 EBs/coverslip/well) or plated on a confluent PA6 feeder layer (50 EBs/well) in 24 well plates. Differentiation media was changed at 48 h intervals. Cells were fixed at required time points in 4% PFA in PBS and processed for immunostaining.

### 4.13. Staining for Primary Cilia and Quantitation

*Ta3^−/−^* iPS and ES cells, as well as *Ta3^fl/fl^* iPS and ES cells, were split 1:5 and seeded on coverslips in complete medium in 24 well plates. Twenty-four hours later, cells were serum-starved for growth arrest by culture in a serum-free medium supplemented with KOSR (DMEM, 1% Glutamax, 1% NEAA and 0.5% KOSR). Cells were fixed after 48 h and immunostained to identify the axoneme (acetylated tubulin and anti-goat Alexa 596 secondary), pericentriolar material (PCM) (Pericentrin, PCNT and anti-mouse Alexa 488) and centriolar satellites (Pericentriolar material protein 1, PCM1 and anti-mouse Alexa 488) of the primary cilia. Images were acquired using a Leica DM5 microscope using z-stacking and deconvoluted. Primary cilia were counted where centriolar structures were adjacent to a length of dense acetylated tubulin but not associated with mitotic structures in dividing cells (e.g., mitotic spindles or midbody). The area occupied by centrosomes was determined by converting the images to binary masks, with a threshold to include only pericentrin, and subsequently measuring the area of each particle. All image analysis was performed using ImageJ. Five random images of each cell line were acquired from three independent experiments. Primary cilia were analysed from cells in the field, with an average of 70 cells/image. Data were compared by one-way ANOVA with Tukey’s post-hoc.

### 4.14. Electroporation and Isolation of Clones Expressing Reporters

Integration-free iPS cells were tagged with CagTag reporter [57]. Feeder-independent *Ta3^−/−^* and *Ta3^fl/fl^* iPS cells (10^7^) were electroporated with the linearised CagTag vector using a BioRad Genepulser at 250 µF and 300 V and plated in five 10 cm plates in ES medium supplemented with LIF (1000 U/mL). Twenty-four hours after electroporation, cells were selected in G418 (Invitrogen, St. Louis, MA, USA, 250 µg/mL). Twenty-four individual clones were picked after 7–8 days of selection into 24 well plates. Mirror plates with coverslips were made for each clone, and nuclear GFP and membrane Tomato were observed on live cells. Clones expressing the positive reporter were expanded, and stocks were frozen. DNA was isolated from each clone and genotyped for GFP.

### 4.15. Composite Embryoid Bodies

Composite EBs were generated by plating 10^6^/mL CagTag reporter expressing iPS or R1 ES cells together with 10^6^/mL Tau-GFP ES cells in the EB medium. Neuronal differentiation was performed as described under ‘Generation of EBs and differentiation’.

### 4.16. Immunostaining

Cell cultures for immunostaining were fixed either in 4% Paraformaldehyde (PFA) or ice-cold Methanol: Acetic acid (3:1) for 20 min. After a further two washes with PBS (10 min), cells were dehydrated through 30, 50 to 70% Ethanol (10 min each) and stored at 4 °C. EBs were fixed for one hour, and all subsequent washes extended to 30 min. Fixed EBs were rehydrated, cryoprotected in 30% Sucrose and embedded in Gelatin and snap frozen. EBs were sectioned (20 µm) using a Leica cryostat, transferred to subbed slides and stored at −20 °C. Prior to immunostaining, sections were allowed to warm to room temperature, then immersed in PBS at 65 °C for three minutes to remove the gelatin and washed twice in PBS.

Immunostaining was performed as described Ferguson et al. [70]. Sections of EBs on slides or rehydrated cells on coverslips were incubated for 1 h at RT in blocking solution (PBS with 0.1% Gelatin, 0.5% FBS and 0.1% Triton-X100) to prevent non-specific binding. They were incubated overnight with primary antibodies (see Supplementary Table S4 for antibodies) diluted in Ab dilution buffer (PBS with 0.1% Triton-X100, 1% FBS) at 4 °C. Sections/cells were washed in PBST (PBS with 0.1% Triton-X100, 4 × 10 min each). This was followed by incubation for two hr at RT with the appropriate secondary antibodies (Supplementary Table S4) diluted in PBST with 1% FBS along with 4′,6-diamidino-2-phenylindole (DAPI) (Invitrogen) to stain cell nuclei. Unbound antibody was removed by washing in PBST (4 × 10 min). Samples were mounted in Mowiol (Polysciences, Warrington, PA, USA).

Z-stacked images were acquired using a Leica DM5500B microscope (Nussloch, Germany), DFC 360FX camera (Leica) and LAS software (v4) and deconvoluted. Images were composed using Adobe Photoshop CS3 (San Jose, CA, USA v5.0). For F-Actin, cells were stained with Alexa Fluor 488 conjugated Phalloidin (1:50, Invitrogen). Images were acquired on a Zeiss LSM 510 META confocal microscope (Oberkochen, Germany) using Zeiss LSM 5 Series software (v4.0).

### 4.17. RNA Isolation and cDNA Synthesis

RNA was extracted from ES, iPS cells and EBs at indicated time points. Feeder-free adherent ES and iPS cells were lysed in 1 ml/cm^2^ Trizol (Invitrogen), scraped, transferred to a centrifuge tube and briefly vortexed to homogenise. Embryoid bodies were lysed in 5 packed cell volumes of Trizol and homogenised by vortexing. One 10 cm dish of EB suspension culture was used per time point. RNA was isolated from the Trizol lysates following the manufacturer’s instructions. RNA yield was determined using a Nanodrop spectrophotometer, and RNA quality was assessed on 1.5% denaturing agarose gel. Equal masses of RNA were re-precipitated and dissolved in equal volumes of DEPC (Sigma) water and then treated with RNase-free DNase (Ambion, Paisley, UK) at 37 °C for 30 min. Quantity and quality were checked again as before. Total RNA was annealed to oligo-dT primers, and cDNA synthesis was performed using M-MuLV H-minus reverse transcriptase for one hour at 42 °C and inactivated at 70 °C for five minutes (RevertAid H Minus Reverse Transcriptase kit, Fermentas, Waltham, MA, USA). Duplicate reactions substituting the RT enzyme for DEPC treated water were also performed as above for each sample. RT reactions were performed at 42 °C for 60 min and inactivated for 5 min at 70 °C. Each reaction was diluted to 10 ng/µL of starting material in sterile MilliQ H_2_O.

### 4.18. RT and Quantitative PCR for Pluripotency and Differentiation Marker Expression

PCR reactions (20 µL) were set up using 2× Biomix (Bioline, London, UK) with 10ng of starting total RNA. Reactions were set up for both ± RT samples where primer pairs did not span exons and only for +RT samples where they did span exons. All primers were used at 0.5 µM final concentration (see Supplementary Tables S5–S8 for primer sequences). Programme conditions were 94 °C for 3 min, then 30 cycles of 30 s at the appropriate annealing temperature, 30 s at 72 °C, and 30 s at 94 °C. PCR products were analysed on a 1.5% Agarose TBE gel alongside low molecular weight DNA size markers (HpaII digested pBlueScript II).

RT-qPCR reactions were carried out in a Bio-Rad iQ5 cycler as described in Ferguson et al. [70]. Briefly, a Mastermix comprising iQ SYBR Green supermix (Bio-Rad, Hercules, CA, USA), water and cDNA was made, and appropriate primers were added and divided into three replicates for each gene on PCR plates (Thermo, Waltham, MA, USA). Final reactions were carried out in a volume of 20 µL with 0.1 µM primers and 10 ng cDNA (see Supplementary Tables S5–S8 for primer sequences). Dynamic well factors were collected for 2 min 30 s, then forty cycles at 60 °C and 95 °C for 20 s each followed by a melt curve. Expression levels were determined relative to GAPDH from baseline subtracted curves and corrected using primer efficiencies determined previously from serial dilutions of PCR product.

### 4.19. Western Blot

Cells were lysed in 2% SDS in 60 mM Tris-Cl pH 6.8, scraped and boiled for 5 min. Lysates were sheared through 21 G and 30 G needles (BD) and then centrifuged at 13 k in a benchtop microfuge. Supernatants were stored at −70 °C. Protein concentration was determined by using a BCA microplate assay following the manufacturer’s instructions (Pierce, Appleton, WI, USA). Twenty µg of each sample was mixed with loading buffer (to final concentrations of 2% SDS, 10% glycerol, 50 mM DTT in 60 mM Tris-Cl pH 6.8), boiled for five minutes, resolved on an 8% acrylamide gel for Gli proteins and on a 10% gel for pluripotency markers. Proteins were transferred to 0.45 µm PVDF membrane at 50 V for 90 min in the transfer buffer (25 mM Tris, 192 mM glycine, pH 8.3, 20% methanol).

Immunoblotting was performed as described in Ferguson et al. [70]. Blots were blocked in 5% Marvel in PBS for an hour then incubated overnight in appropriate primary antibody (See Supplementary Table S4 for antibody sources and dilutions) diluted in 5% Marvel in PBS at 4 °C. After four 5 min washes in PBS with 0.1% Tween20 (PBSTw) the blots were incubated with appropriate Horseradish peroxidase-conjugated secondary antibody (1:10,000, Abcam, Cambridge, MA, USA) diluted in 5% Marvel in PBS. After further 4 × 5 min washes in PBSTw, blots were incubated with ECL reagent and exposed to film (Amersham hyperfilm, Amersham, UK). Blots were imaged on a Fusion SL (Vilber Lourmat, Paris, France) using the associated Fusion-Capt software. Exposure times were set based on software predictions. Quantification of relative densities and estimation of molecular mass were performed for three independent experiments using the same software.

## Data Availability

Dataset available on request from the authors.

## Supplementary Materials

The following supporting information can be downloaded at: https://www.mdpi.com/article/10.3390/cells13231957/s1, Figure S1: Further characterization of ES and iPS cell lines; Figure S2: F-Actin organization in ES and Excised iPS cells; Figure S3: Cell cycle analysis of *Ta3^fl/fl^* and *Ta3^−/−^* ES and iPS cells; Figure S4: Markers for primary germ layer derivatives; Figure S5: Time course of Afp and Hh expression during EB differentiation; Figure S6: Time course of neuronal differentiation of EBs on Matrigel; Figure S7: Composite EBs show that loss of *Ta3* and cilia causes intrinsic defects in differentiating neurons; Table S1: Analysis of pOSKML reprogramming cassette integration sites; Table S2: Antibodies; Table S3: PCR primers for Genotyping; Table S4: RT-PCR primers for pluripotency associated transcripts; Table S5: Oligos and primers for splinkerette PCR; Table S6: RT- PCR primers for limb associated transcripts; Table S7: RT-PCR primers for differentiation associated transcripts; Table S8: RT-PCR primers for Hh signaling associated transcripts. References [71,72] are cited in the Supplementary Materials.
