# Supplementary material for: Targeted Deletion in the Basal Body Protein Talpid3 Leads to Loss of Primary Cilia in Embryonic Stem Cells and Defective Lineage-Specific Differentiation
Source: Cells. 2024 Nov 25;13(23):1957. doi: 10.3390/cells13231957 (PMC11639927; doi:10.3390/cells13231957)

Targeted deletion in the basal body protein Talpid3 leads to loss of primary cilia in embryonic stem cells and defective lineage-specific differentiation

Ross Ferguson and Vasanta Subramanian *

Department of Biology and Biochemistry, Building 4 South, University of Bath, Bath BA2 7AY, UK

Supplementary Information

F-actin Distribution in *Ta3*$^{-/-}$ Cells

R1 and *Ta3*$^{fl/fl}$ cells have a uniform ring of F-actin in the cell while *Ta3*$^{-/-}$ cells have a more uneven distribution (Figure S2A). Extensively organised F-actin was observed in R1 and *Ta3*$^{fl/fl}$ cells, while short, highly disorganised F-actin patches were seen in *Ta3*$^{-/-}$ cells. At a gross level, *Ta3*$^{-/-}$ ES and iPS have a morphology similar to R1 and *Ta3*$^{fl/fl}$ cells; however, we noted that *Ta3*$^{-/-}$ colonies frequently appeared tighter with a more defined boundary. Quantification of this feature by comparing the smoothness of the colony perimeter showed that *Ta3*$^{-/-}$ ES and iPS colonies were significantly smoother boundaries than R1 and *Ta3*$^{fl/fl}$ colonies (Figure S2C). This phenotype could not be induced in R1 and *Ta3*$^{fl/fl}$ colonies by the addition of the Hh signalling inhibitor cyclopamine at 2 μM for 6 h.

The Absence of Primary Cilia does not Affect the Cell Cycle of *Ta3*$^{-/-}$ ES and iPS Cell Lines Cells

Ta3$^{-/-}$ ES and iPS cell lines grew at a similar rate to the R1 ES and *Ta3*$^{fl/fl}$ ES and iPS cells. Staining for F-actin and acetylated tubulin did not reveal morphological defects in the mitotic phases (Figure S2B), and no significant differences in the cell cycle were found by FACS analysis between R1 ES and *Ta3*$^{fl/fl}$ or *Ta3*$^{-/-}$ ES and iPS cells (Figure S3).

Ta3$^{-/-}$ ES and iPS Cells Generate Cell Types of All Three Germ Layers

Ta3$^{-/-}$ ES and iPS cells are able to generate cell types of the three germ layers as seen by the immunostaining for markers of these germ layers. Vimentin, a marker for meso- and endo-dermal lineages was present from day six in the outer layer of EBs and around the apical membrane of cells forming lumens (Figure S5A). CK18 and CK19, expressed by ecto- and endodermal cells, were detectable by day eight in the outer cell layer of both *Ta3*$^{fl/fl}$ and *Ta3*$^{-/-}$ EBs. The pattern of expression, however, was markedly different as it was uniformly present in the outer cell layer in *Ta3*$^{fl/fl}$ EBs but remained patchy and disorganised in Ta3$^{-/-}$ EBs. Msx1/2 expressing cells were present in both *Ta3* $^{fl/fl}$ and *Ta3*$^{-/-}$ EBs by day twelve (Figure S5B). Myogenin and myosin heavy chains, which mark differentiated derivatives of the mesoderm, were also detectable in both *Ta3*$^{fl/fl}$ and *Ta3*$^{-/-}$ EBs.

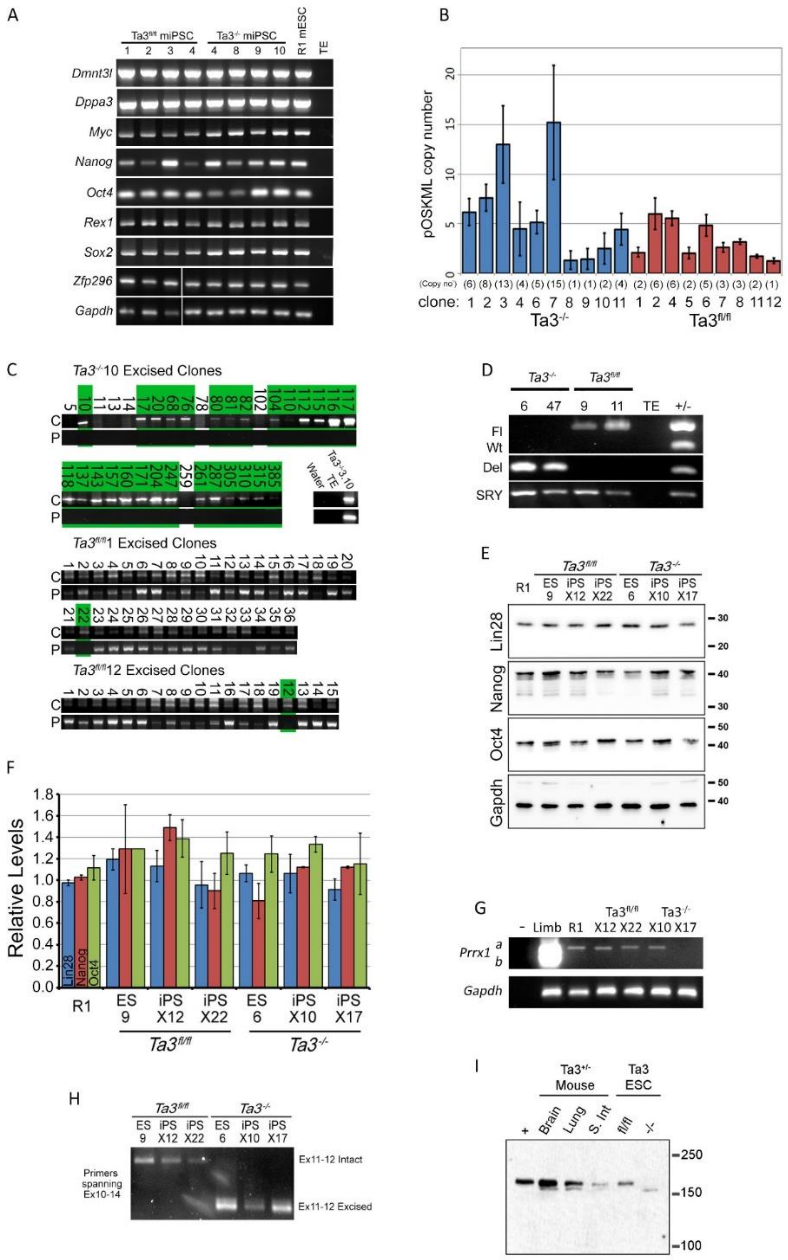

Figure S1. Further characterisation of ES and iPS cell lines. (**A**) Expression of pluripotency markers in transgene carrying iPS cells. *Ta3*$^{-/-}$, *Ta3*$^{fl/fl}$ and wildtype CD1 iPS cell lines express multiple pluripotency-associated transcripts at levels comparable to R1 ES. *Actb* and *Gapdh* were used as loading controls. (**B**) Copy Number of integrated *OSKML* and confirmation of excision. Reprogramming vector (*pOSKML*) copy number in each *Ta3* iPS cell line determined by qPCR for a vector-specific fragment with reference to an endogenous target. Numbers in the brackets indicate estimated copy numbers. (**C**) *Ta3* iPS Clones in which excision of *pOSKML* had occurred were identified by PCR for the *puromycin* resistance gene (p). DNA integrity was confirmed by PCR for an endogenous control (c). Transgene-free clones are highlighted in green. 400 clones screened for *Ta3*$^{-/-}$ 10, and 96 screened for Ta3$^{fl/fl}$ 1 & 12 each. (**D**) Feeder-free ES cells derived from blastocysts obtained from a *Ta3*$^{+/-}$ *X Ta3*$^{+/-}$ or *Ta3*$^{fl/fl}$ *X Ta3*$^{fl/fl}$ mouse cross were genotyped by PCR to identify ES lines homozygous for the *Ta3* deleted (del) or floxed (fl) allele. The sex of selected ES cell lines was determined by PCR for the Y-specific *SRY* sequence. (+/−) control template DNA from a *Ta3*$^{+/-}$ or *Ta3*$^{+/fl}$ mouse. (**E**) Western Blots of ES cells for pluripotency markers. Western blots of pluripotency-associated proteins Oct4, Nanog and Lin28 in *Ta3*$^{-/-}$ and *Ta3*$^{fl/fl}$ ES and transgene-free iPS cells. Note the absence of higher mass species in Lin28 and Oct4 blots, which indicates the absence of the T2A and F2A tagged species coming from the reprogramming vector. (**F**) Quantification of pluripotency associated protein Lin28 (blue), Nanog (red) and Oct4 (green) protein levels in R1, *Ta3*$^{fl/fl}$ iPS X12 and X22, *Ta3*$^{fl/fl}$ ES 9, *Ta3*$^{-/-}$ iPS X10 and X17, and *Ta3*$^{-/-}$ ES 6 cell lines. Data normalised to Gapdh and presented relative to levels in R1 ES cells. Quantified from three independent experiments. Error bars ±SEM. (**G**) Expression of the limb marker Prrx1a and b is lost in R1 and excised iPS clones, in comparison to embryonic limb fibroblasts. (−) no template. (**H**) RT-PCR was performed using primer positions in exons 10 and 14 of the Ta3 gene product, flanking the floxed exon 11-12 region; a truncated transcript remains in Ta3 deletion cells. (**I**) Western blot for Ta3 shows a truncated protein product in heterozygous mouse tissue samples and homozygous mouse ES cells lacking the critical exon 11-12 coiled-coil domain. S.Int -small intestine (+) recombinant Ta3, a kind gift from Prof. Ravi Acharya.

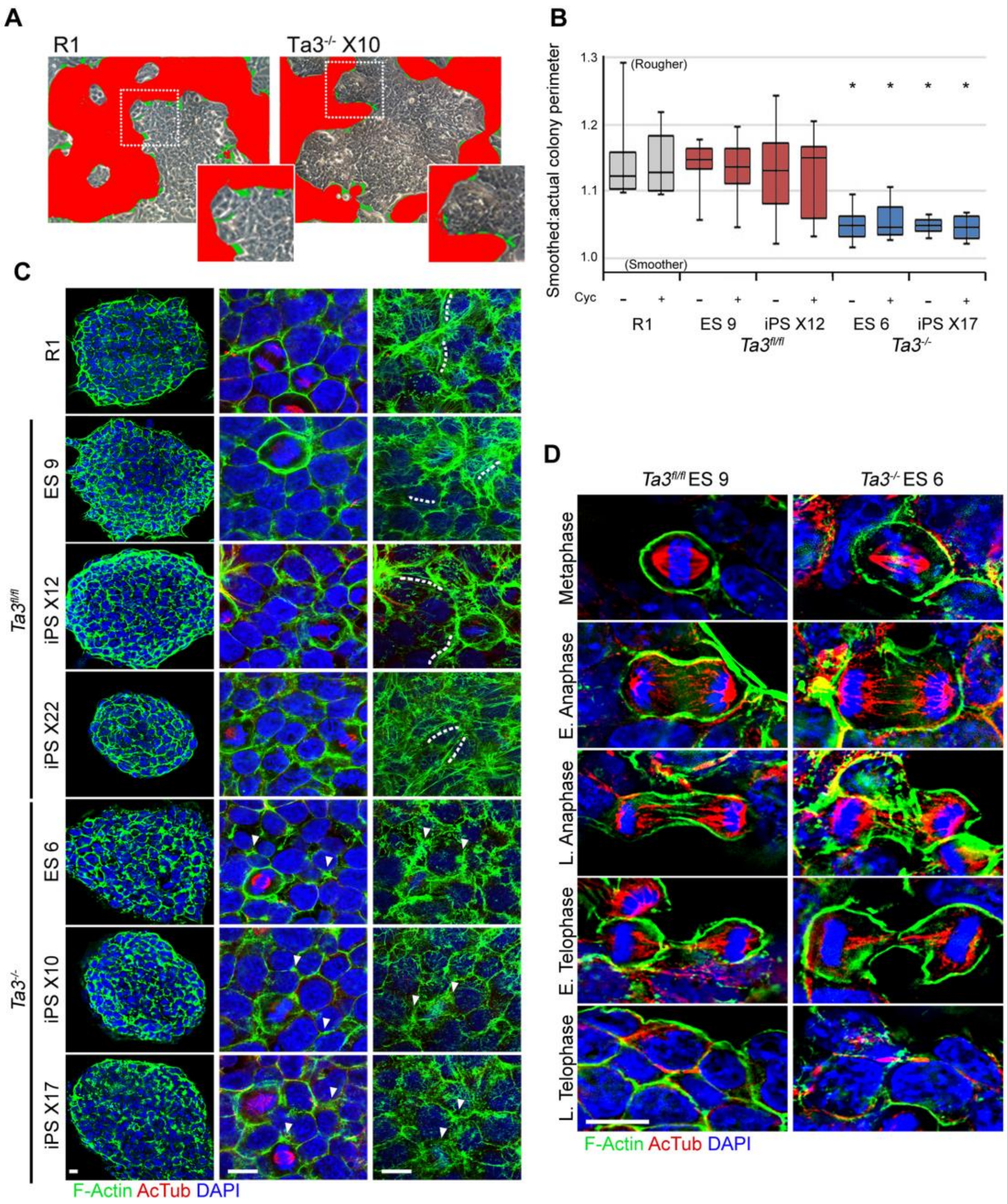

Figure S2. F-Actin organisation in ES and Excised iPS cells. (**A**) Colony edge bounded by the green mask was measured as actual perimeter. The green mask was then smoothed with a 50 μm radius to create the red mask and measured as the smoothed perimeter. Less of the green mask can be seen around the Ta3$^{-/-}$ colony, indicating the colony boundary conforms better to the smoothed 'perfect' boundary. (**B**) The ratio of smoothed to actual perimeter was calculated as an indicator of roughness, where a ratio of 1 indicates a perfectly smooth perimeter while values above 1 indicate roughness. 100 colonies of each cell line were measured from three independent experiments. Smoothed-actual perimeters compared by ANOVA with Tukey's post-hoc. * $P < 0.05$. No effect on WT morphology was seen upon incubation with the Hh pathway antagonist cyclopamine (Cyc) prior to analysis. (**C**) Phalloidin labelling showing organised filaments of F-Actin in R1 ES, *Ta3$^{fl/fl}$* ES and iPS cells (dotted lines), and disorganised staining in Ta3$^{-/-}$ ES and iPS cells (arrows) in different optical z-slices of the same colony. Left image; representative colony, centre image; optical section through cell mass, right image; optical section showing cell membrane adjacent to the coverslip. (**D**) Representative images of mitotic phases of *Ta3$^{fl/fl}$* ES 9 and Ta3$^{-/-}$ ES 6; Metaphase, early to late anaphase, and early to late telophase.

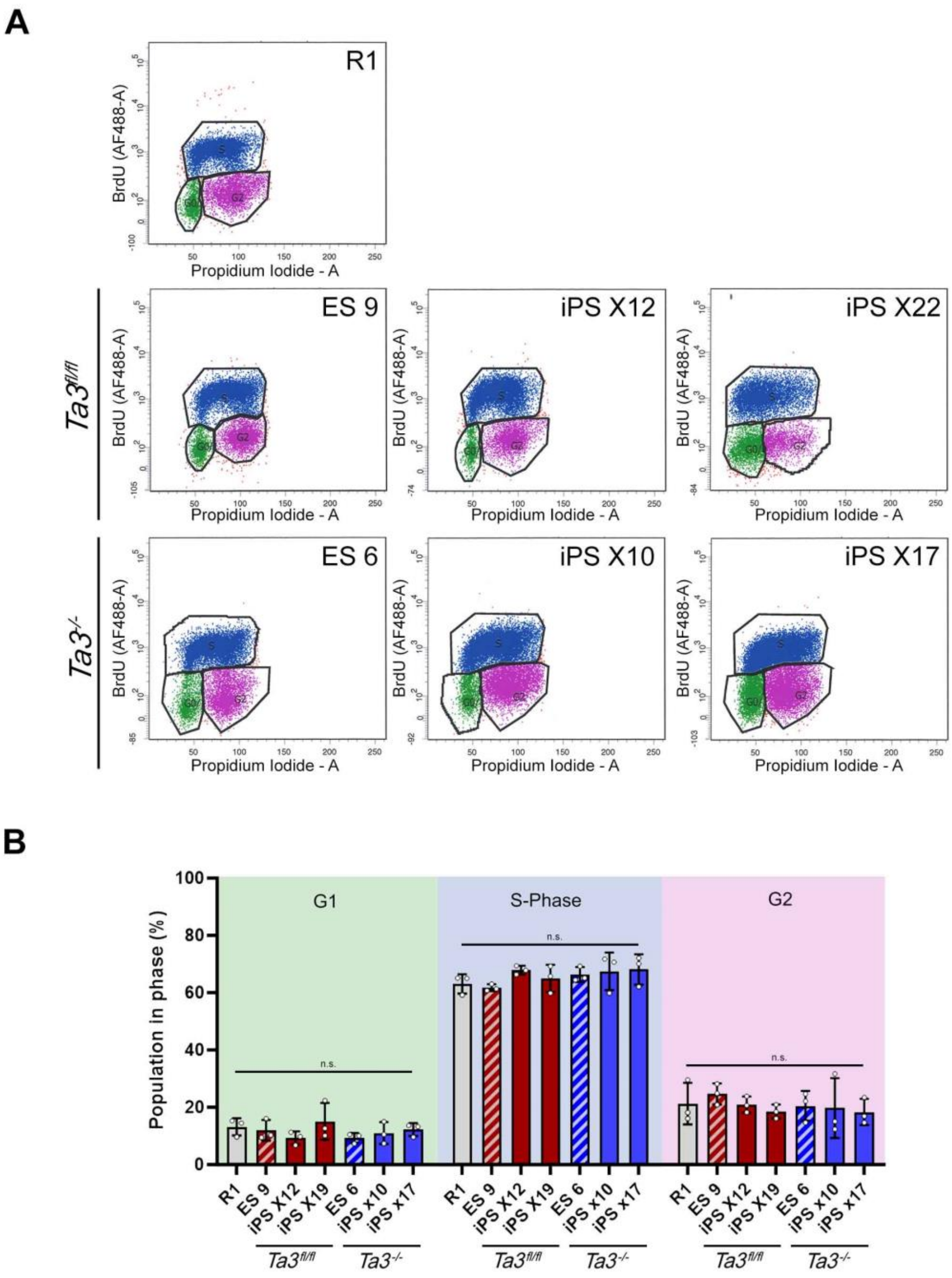

Figure S3. Cell cycle analysis of *Ta3*$^{fl/fl}$ and *Ta3*$^{-/-}$ ES and iPS cells. (**A**) Representative FACS dot plots showing log AF488 fluorescence (BrdU incorporation) against Propidium Iodide fluorescence to distinguish cells in G0/1 (green), S (blue) and G2 phase (magenta). Single-cell events are gated by comparison of PI signal width to area. Ten thousand events are shown in each plot. (**B**) The mean (bar, errors ± SD) cell cycle distributions from three independent analyses (points) of R1, *Ta3*$^{fl/fl}$ iPS, *Ta3*$^{fl/fl}$ ES cells, *Ta3*$^{-/-}$ iPS and *Ta3*$^{-/-}$ ES cells. N.s. no significant difference by ANOVA with Tukey's post-hoc.

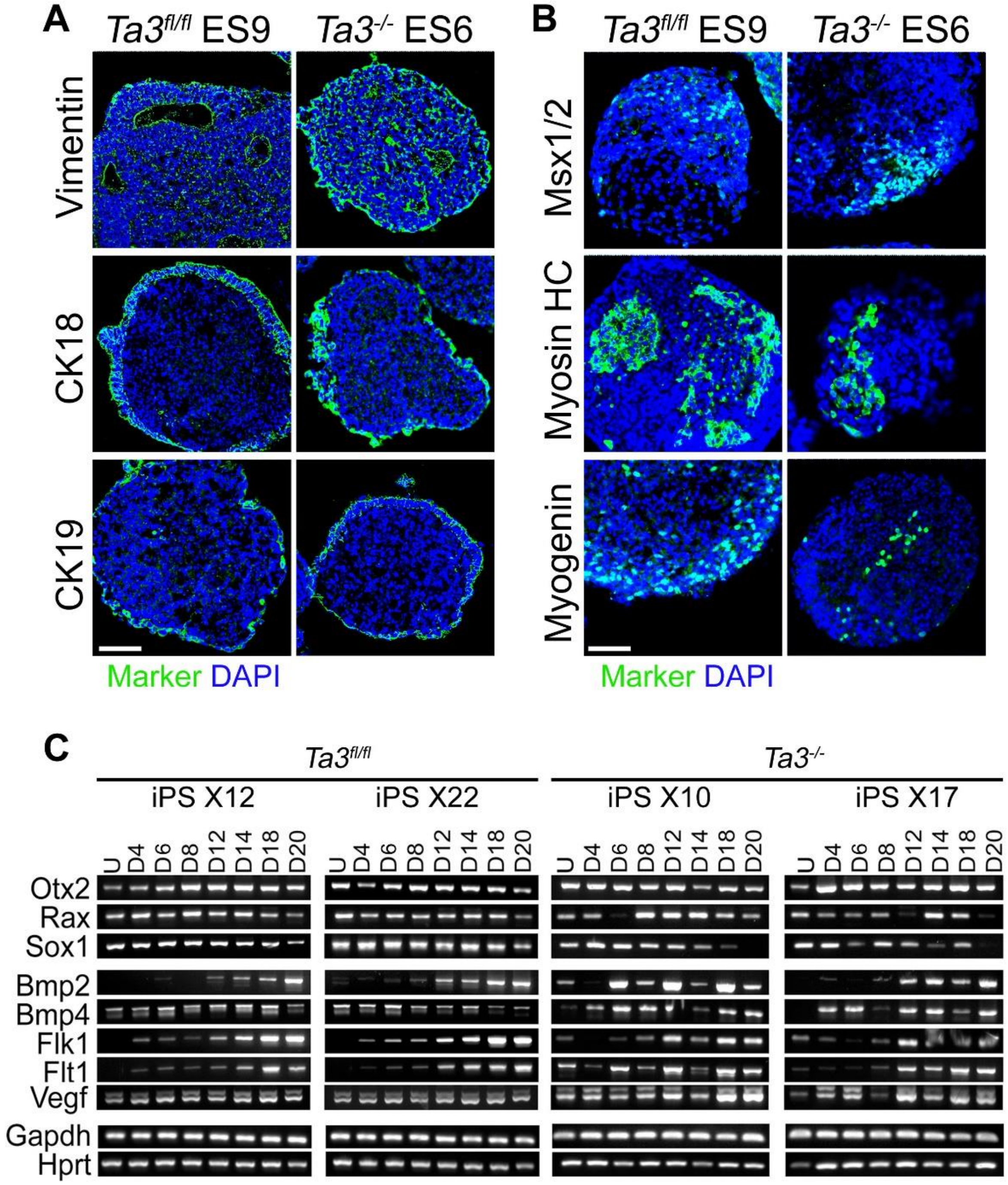

Figure S4. Markers for primary germ layer derivatives (Related to Figure 5). Representative images of immunostained sections of *Ta3*$^{fl/fl}$ ES9 and *Ta3*$^{-/-}$ ES6 EBs on the days indicated. (**A**) Immunostaining for expression of Vimentin (endo/mesoderm) and Cytokeratin 18 and 19 (ecto/endoderm) on day 8 EBs. Scale bar: 50 μm. (**B**) Msx1/2 (mesoderm) and Myosin Heavy Chain (mesoderm) on day 12 and Myogenin (mesoderm) on day 14 EBs. Scale bar: 50 μm. (**C**) RT-PCR for expression of markers of primary germ cell layer derivatives in EBs derived from Ta3 $^{fl/fl}$ and *Ta3*$^{-/-}$ iPS cells. Ectoderm markers - Otx2, Rax1 and Sox1; Mesodermal derivative markers - Bmp2/4, Flk1, Flt1 and VEGF. Gapdh and Hprt were used as loading controls.

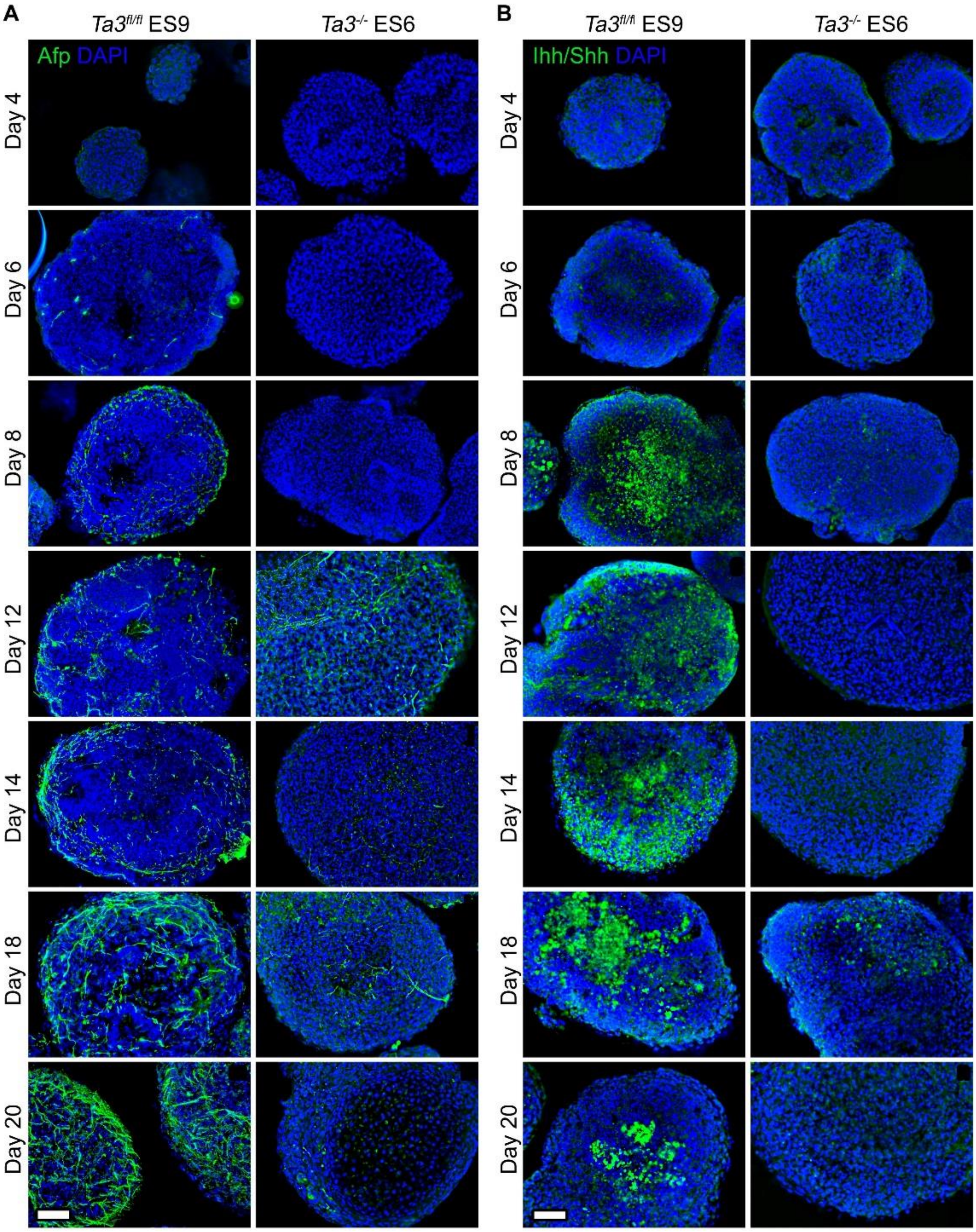

Figure S5. Time course of Afp and Hh expression during EB differentiation. (**A**) Afp expression in EBs. Afp expression was first detected on day eight in *Ta3*$^{fl/fl}$ EBs expression in the outer cell layers *Ta3*$^{fl/fl}$ EBs from days twelve to twenty and as patches in the interior from days eighteen to twenty; Afp expression seen transiently on day twelve *Ta3*$^{-/-}$ EBs; later expression restricted to sporadic cells in the interior of the EBs. (**B**) Hh expression *Ta3*$^{fl/fl}$ and *Ta3*$^{-/-}$ ES-derived EBs. (**B**) Early low Hh signal was detected on days four and six in both genotypes, but robust Hh expression was seen only from day eight in the interior of the *Ta3*$^{fl/fl}$ EBs; Expression increases throughout the *Ta3*$^{fl/fl}$ EBs from days twelve to eighteen and as patches in the interior of the EB on day twenty; Low-level Afp expression seen transiently in day eight *Ta3*$^{-/-}$ EBs; Later expression restricted to sporadic cells in the interior of the EBs. Scale bars: 50 μM.

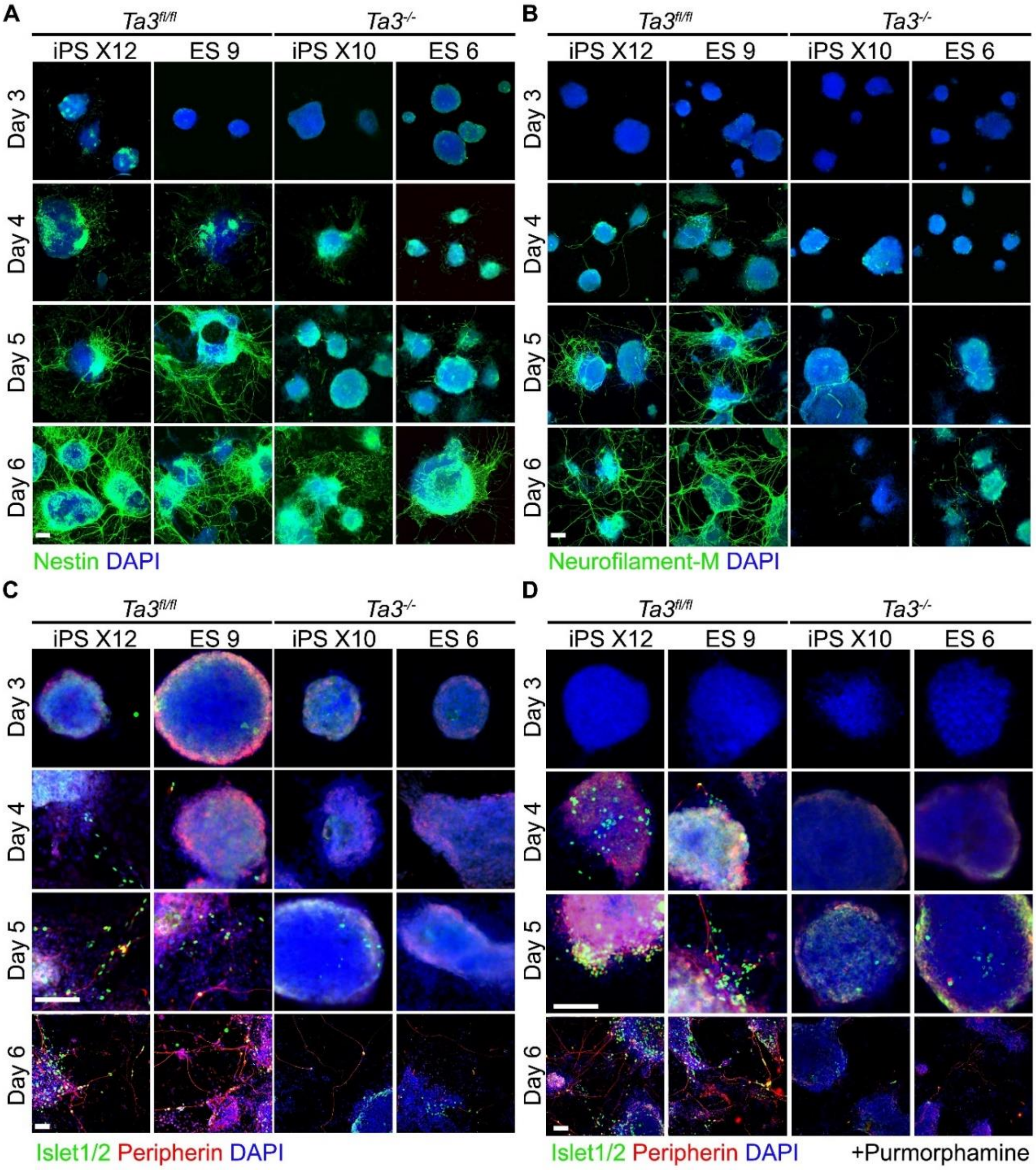

Figure S6. Time course of neuronal differentiation of EBs on Matrigel. RA-treated EBs plated on Matrigel immunostained for nestin (neural progenitors), NF (mature neurons), and islet1/peripherin (postmitotic MNs). (**A**) Nestin was first detected on day four in *Ta3*$^{fl/fl}$ EBs and, to a lesser extent, in *Ta3*$^{-/-}$ EBs. Extensive networks of nestin-positive neurites were present in the *Ta3*$^{fl/fl}$ EBs and less noticeably in the *Ta3*$^{-/-}$ EBs. (**B**) NF expression was first detected on day four in a small number of *Ta3*$^{fl/fl}$ cells; increased numbers of NF-positive cells between days five and six with long neurites extending away from EBs. NF expression was seen in a very small number of neurons from day five *Ta3*$^{-/-}$ EBs with no increase over the time course. (**C**) Motor neurons, identified by Islet expression, appear in small numbers on day four in *Ta3*$^{fl/fl}$ EBs. They increase in frequency over the course of the differentiation, and on day six, islet-positive cells are found mainly at the edges of EBs, with peripherin-positive neurites projecting out. *Ta3*$^{-/-}$ iPS X10 EBs have very few neurons, mostly with short peripherin-positive neurites and some Islet-positive nuclei on days five and six. (**D**) The inclusion of purmorphamine in the differentiation medium results in a substantial increase in the number of islet/peripherin-positive motor neurons present in EBs generated from *Ta3*$^{fl/fl}$ EBs clones. *Ta3*$^{-/-}$ EBs are refractory to purmorphamine. Scale bar: 100 μm.

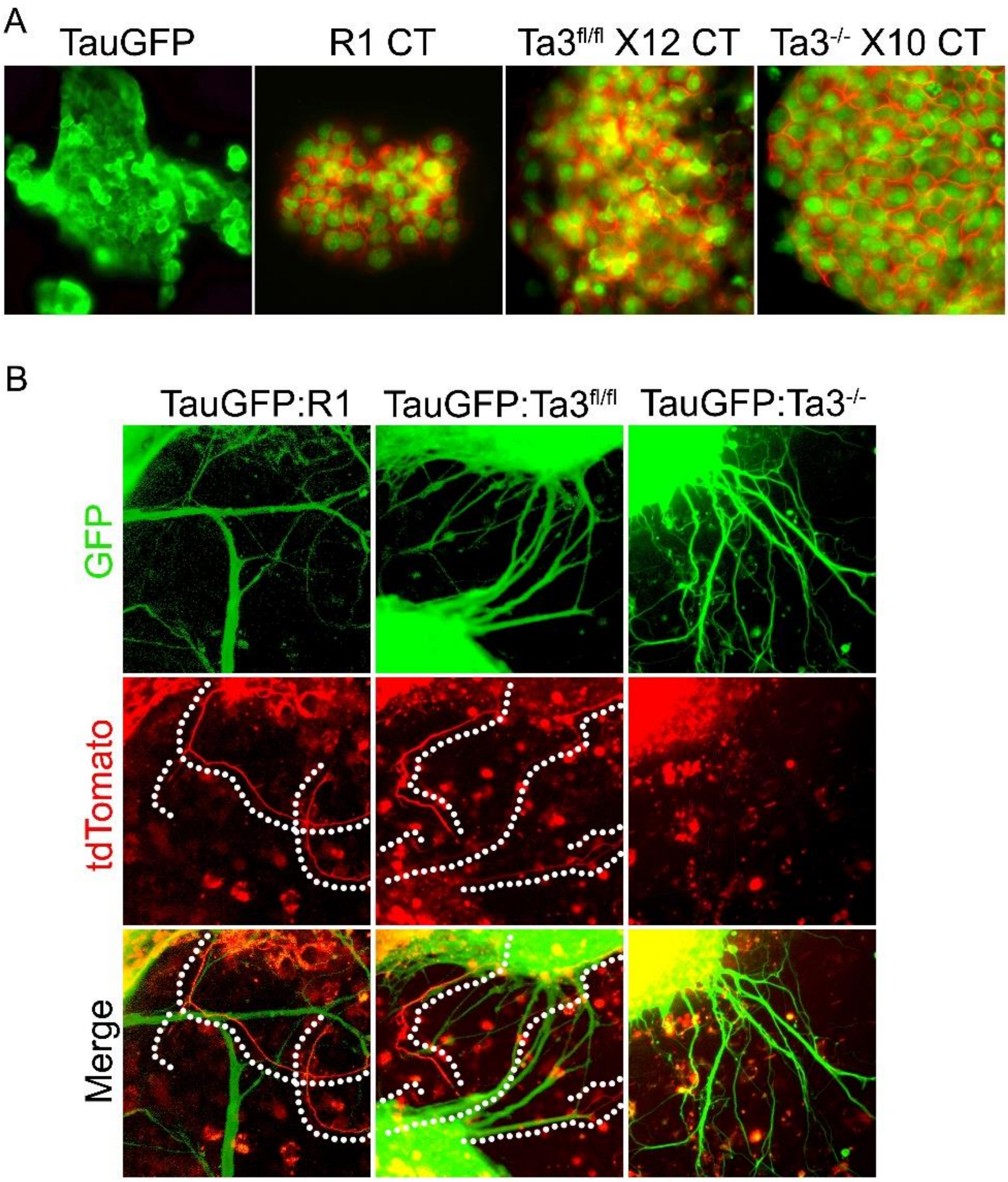

Figure S7. Composite EBs show that loss of Ta3 and cilia causes intrinsic defects in differentiating neurons. (**A**) ES cells expressing GFP tagged Tau (TauGFP) and R1 ES or $Ta3^{fl/fl}$ X12 and $Ta3^{-/-}$ X10 iPS tagged with CagTag (CT). The CagTag reporter expresses GFP-tagged histone2b and myristoylated tfTomato targeted at the membrane. (**B**) Neurite outgrowth on matrigel on day twelve from composite EBs comprised 50:50 of each indicated cell line.

Supplemental Table S1. Analysis of pOSKML reprogramming cassette integration sites.

Ta3 $^{-/-}$ **Clone 3.8 (Ta3** $^{-/-}$ **X17 parent clone)**				
**Integration site**	**Chr.**	**Splinkerette fragment range**	**5′ Features**	3′ Features
1	13	104679102 to 104679322	49096 bp at 5′ side: ADAM-TS6 disintegrin-like and metallopeptidase	88762 bp at 3′ side: CWC27 spliceosome-associated protein

Ta3 $^{-/-}$ **Clone 3.10** (Ta3 $^{-/-}$ X10 parent clone)				
Integration site	Chr.	Splinkerette fragment range	5′ Features	3′ Features
1	5	4796060 to 4796183	70851 bp at 5' side: UPF0454 protein C12orf49 homolog precursor	228729 bp at 3' side: mediator of RNA polymerase II transcription subunit 13-like
2	2	107287520 to 107287541	275399 bp at 5' side: extracellular sulfatase Sulf-2 isoform 1 precursor	159626 bp at 3' side: phosphatidylinositol 3,4,5-trisphosphate-dependent Rac

Ta3 $^{fl/fl}$ **Clone 1** (Ta3 $^{fl/fl}$ X12 parent clone)				
Integration site	Chr.	Splinkerette fragment range	5′ Features	3′ Features
1	X	25954152 to 25954271	35291 bp at 5' side: uncharacterised protein LOC667794	103702 bp at 3' side: thymosin beta-4
2	11	75044087 to 75044425	3992 bp at 5' side: protein FAM20A precursor	62669 bp at 3' side: uncharacterised protein LOC69324 isoform 1
3	8	54068936 to 54069117	118247 bp at 5' side: interferon regulatory factor 2-binding protein 2	223151 bp at 3' side: mitochondrial import receptor subunit TOM20 homolog

Ta3 $^{fl/fl}$ **Clone 12** (Ta3 $^{fl/fl}$ X22 parent clone)				
Integration site	Chr.	Splinkerette fragment range	5′ Features	3′ Features
1	17	4022550 to 4022609	8857 bp at 5' side: olfactory receptor 120	15953 bp at 3' side: olfactory receptor 121

Supplemental Table S2. Antibodies.

Primary Antibody	Dilution	Supplier	Cat#
Oct4	1:1000	Abcam	ab27985
Nanog	1:1000	Millipore	AB5731
SSEA1	1:500	DSHB	SSEA1
Lin28	1:200	Cell Signalling	3978S
Pericentrin	1:1000	Abcam	ab4448
Acetylated Tubulin	1:1000	Sigma	T7451
Nestin	1:200	DSHB	Rat401
Neurofilament	1:5	DSHB	2H3
Islet1/2	1:5	DSHB	40.2D6
Peripherin	1:1000	Millipore	AB1530
Hedgehog	1:5	DSHB	5E1
PKCz	1:1000	Abcam	ab51157
Laminin B1	1:500	Santa Cruz	SC6018
Afp	1:200	Cell Signalling	3903S
Cytokeratin 8	1:5	DSHB	Troma1
ZO1	1:5	DSHB	R26.4C
Gli1	1:500 (IF)<br>1:1000 (W)	Cell Signalling	#2534
Gli2	1:1000 (W)	Abcam	ab26056
Gli3	1:500 (W)	Santa Cruz	sc-20688
Cytokeratin18	1:50	Birgit Lane	LE61
Cytokeratin 19	1:50	Birgit Lane	LP2K
Vimentin	1:100	Sigma	V6630
Myosin heavy chain	1:5	DSHB	F59
Myogenin	1:5	DSHB	F5D
BrdU	1:50 (FACS)	DSHB	G3G4
PCM1	1:500	Santa Cruz	sc-67204
Msx1/2	1:1	DSHB	4G1
Arl13b	1:1500	Proteintech	17711-1
Gt α Ms AF488	1:2000	Invitrogen	A-11001
Gt α Rbt AF594	1:2000	Invitrogen	A-11008
Gt α Rbt AF488	1:2000	Invitrogen	A-11012
Gt α Ms AF568	1:2000	Invitrogen	A-11036

Supplemental Table S3. PCR primers for Genotyping.

Primer	Sequence (5′-3′)	Ta (°C)	Product (bp)
Klf4-F2A F	TGGGTGGAAATTCGCCCGCT	60	195 (q)
Klf4-F2A R	TCCACGTCTCCCGCCAACTT		
Puro F	GCTCGACATCGGCAAGGTGT	60	129
Puro R	GAACCGCTCAACTCGGCCAT		
1647_31 (TA)	TGCCATGCAGGGATCATAGC	60	273
1738_35 (TA)	GAGCACACTGGAGGAAAGC		
1260_1 (TA)	GAGACTCTGGCTACTCATCC	60	585
1260_2 (TA)	CCTTCAGCAAGAGCTGGGGAC		
1647_31 (TA)	TGCCATGCAGGGATCATAGC	60	351 (WT), 470 (fl/fl)
1647_32 (TA)	GCTAGTACATTGCTGCAAGC		
mCdx F	CACAGGTAAAGATCTGGTTCCAGAA	60	276 (q)
mCdx R	AGCCAGCTGCCCAGCATTCA		

Primers designed by Taconic Artemis (TA).
* Transcript and genomic products have the same length. (q) Primer pairs used for qPCR.

Supplemental Table S4. RT-PCR primers for pluripotency-associated transcripts.

Primer	Sequence (5′-3′)	Ta (°C)	Product (bp)
mNanog RT F (a)	CCACAGTTTGCCTAGTTCTGAGGAAGCATC	60	504 (q)
mNanog RT R (a)	TACTCCACTGGTGCTGAGCCCTTCTGAATC		
mEcat1 RT F (a)	CGTGGAACCTCGGCTACTGGAAATC	55	255
mEcat1 RT R (a)	GCCGCCATACGACGACGCTCAACTC		
mEsrrb1 RT F (a)	AACCTGCCGATTTCCCCACCTGCTA	55	232
mEsrrb1 RT R (a)	GGCTCATCTGGTCCCCAAGTGTCAG		
mDppa3 RT F (a)	GAGGACGCTTTGGATGATACAGACG	55	210*
mDppa3 RT R (a)	CAACAAAGTGCGGACCCTTCTCTTG		
mDnmt3l RT F (a)	CCCTCTTCCTGTATGATGATGATGG	60	221
mDnmt3l RT R (a)	CCTCTGCAGCAGTCCACTCCGTGAG		
mActb RT F (a)	CAGGGTGTGATGGTGGGAATGGGTCAGAAG	60	282
mActb RT R (a)	TACGTACATGGCTGGGGTGTTGAAGGTCTC		
mGapdh RT F (a)	CCTTCCGTGTTCCTACCCCCAATG	55	155 (q)
mGapdh RT R (a)	GGAGACAACCTGGTCCTCAGTGTA		
mZfp296 RT F (b)	CCTATGCTTGTGCCCAGAGTA	55	213*
mZfp296 RT R (b)	CTAAAGTGCCTGCCCATTTC		
mRex1 RT R (b)	GGAAGAAATGCTGAAGGTGGAGAC	55	263*
mRex1 RT F (b)	AGTCCCCATCCCCTTCAATAGC		
mFoxD3 RT F (b)	AGGTCTGACCCCGAACAAG	55	63*
mFoxD3 RT R (b)	AGCGCGATGTAAGAGTAGGG		
mKlf4 RT F (b)	GGCGAGAAACCTTACCACTGT	60	266*
mKlf4 UTR RT R (b)	TACTGAACTCTCTCTCCTGGCA		
mOct4 RT F (b)	CCAACGAGAAGAGTATGAGGC	60	392* (q)
mOct4 UTR RT R (b)	CAAAATGATGAGTGACAGACAGG		
mSox2 RT F (b)	TCTGTGGTCAAGTCCGAGGC	60	254* (q)
mSox2 UTR RT R (b)	TTCTCCAGTTCGCAGTCCAG		
mcMyc RT F (b)	TCAAGCAGACGAGCACAAGC	60	242* (q)
mcMyc UTR RT R (b)	TACAGTCCCAAAGCCCCAGC		

Primer sequences from (a) Yusa K. et al., 2009[3] or (b) Woltjen K. at el., 2009[4].
* Transcript and genomic products have the same length. (q) Primer pairs used for qPCR.

Supplemental Table S5. Oligos and primers for splinkerette PCR.

Splinkerette/Primer	Sequence (5′-3′)
Spl-top [(a)]	CGAATCGTAACCGTTCGTACGAGAATTCGTACGA-GAATCGCTGTCCTCTCCAACGAGCCAAGG
Spl-sau [(a)]	GATCCCTTGGCTCGTTTTTTTTTGCAAAAA
Spl-CG [(a)]	CGCCTTGGCTCGTTTTTTTTTGCAAAAA
Spl-blunt [(a)]	CCTTGGCTCGTTTTTTTTTGCAAAAA
Spl-P1 [(a)]	CGAATCGTAACCGTTCGTACGAGAA
PB3-P1 [(a)]	AAACCTCGATATACAGACCGATAAAACAC
PB5-P1 [(a)]	AAGCGGCGACTGAGATGTCCTAAATG
Spl-P2 [(a)]	TCGTACGAGAATCGCTGTCCTCTC
PB3-P2 [(a)]	CGTCAATTTTACGCATGATTATCTTTAAC
PB5-P2 [(a)]	GCGACGGATTCGCGCTATTTAGAAAG

Primer sequences from (a)Yusa K. et al., 2009[3].

* Transcript and genomic products have the same length. (q) Primer pairs used for qPCR.

Supplemental Table S6. RT- PCR primers for limb-associated transcripts.

Primer	Sequence (5′-3′)	Ta (°C)	Product (bp)
mPrrx1 F	CCCGGATGCTTTTGTTCGAGA	60	273/345/592
mPrrx1 R	CATGTGGCAGAATAAGTAGCCAT		Transcripts 1/2/3
mHoxd13 F	GGGCTACCAGTCCTGGACGCT	60	149 (q)
mHoxd13 R	TTCCCCGTCGGTAGACGCACA		
mMeis1 F	GGGCGTGGCTGTTCCAGCAT	60	240 (q)
mMeis1 R	TGCCCATGTGCTGCTGACCG		

* Transcript and genomic products have the same length. (q) Primer pairs used for qPCR.

Supplemental Table S7. RT-PCR primers for differentiation-associated transcripts.

Primer	Sequence (5′-3′)	Ta (°C)	Product (bp)
AFP F(c)	GCTCACACCAAAGCGTCAAC	60	411 (q)
AFP R(c)	CCTGTGAACTCTGGTATCAG		
Hbb-bh1 F(c)	AGTCCCCATGGAGTCAAAGA	60	265
Hbb-bh1 R(c)	CTCAAGGAGACCTTTGCTCA		
HPRT F(c)	CACAGGACTAGAACACCTGC	60	249
HPRT R(c)	GCTGGTGAAAAGGACCTCT		

Primer sequences from (c)Maye P. et al., 2000[5].
* Transcript and genomic products have the same length. (q) Primer pairs used for qPCR.

Supplemental Table S8. RT-PCR primers for Hh signaling-associated transcripts.

Primer	Sequence (5′-3′)	Ta (°C)	Product (bp)
Gli1 F(c)	TTCGTGTGCCATTGGGGAGG	60	440 (q)
Gli1 R(c)	CTTGGGCTCCACTGTGGAGA		
Gli2 F(c)	TTCGTGTGCCGCTGGCAGGC	60	425 (q)
Gli2 R(c)	TTGAGCAGTGGAGCACGGAC		
Gli3 F(c)	TTCGTGTGCCGCTGGCTTGA	60	444 (q)
Gli3 R(c)	TGAATGGCTGCCGGAATCTC		
Ptc1 F(c)	GGTCACACGAACAATGGGTCT	60	682 (q)
Ptc1 R(c)	CACATTCCACGTCCTGTAGC		
Ptc2 F(c)	TCCAAGGTCTACTCTTCTCC	60	555 (q)
Ptc2 R(c)	GCTCCTCGAGCAGCTGCTGA		
Smo F(c)	TGGGATCCAGTGCCAGAACCCGCT	60	562 (q)
Smo R(c)	ACGGTACCGATAGTTCTTGTAGCC		
Ihh F	CTGCAAGGACCGTCTGAACT	60	270 (q)
Ihh R	GCCGAATGCTCAGACTTGAC		
Shh F	GTGATCCTTGCTTCCTCGCT	60	281 (q)
Shh R	TTGCACCTCTGAGTCATCAGC		

Primer sequences from (c)Maye P. et al., 2000[5].
* Transcript and genomic products have the same length. (q) Primer pairs used for qPCR.

Supplementary References

1. Pratt, T., Sharp, L., Nichols, J., Price, D. J. & Mason, J. O. Embryonic stem cells and transgenic mice ubiquitously expressing a tau-tagged green fluorescent protein. Dev. Biol. 228, 19–28 (2000).
2. Trichas, G., Begbie, J. & Srinivas, S. Use of the viral 2A peptide for bicistronic expression in transgenic mice. BMC Biol. 6, 40 (2008).
3. Yusa, K., Rad, R., Takeda, J. & Bradley, A. Generation of transgene-free induced pluripotent mouse stem cells by the piggyBac transposon. Nat. Methods 6, 363–369 (2009).
4. Woltjen, K. et al. piggyBac transposition reprograms fibroblasts to induced pluripotent stem cells. Nature 458, 766–770 (2009).
5. Maye, P., Becker, S., Kasameyer, E., Byrd, N. & Grabel, L. Indian hedgehog signaling in extraembryonic endoderm and ectoderm differentiation in ES embryoid bodies. Mech. Dev. 94, 117–132 (2000).

Full gel and blot images

Figure 1. Ta3$^{fl/fl}$ and Ta3$^{-/-}$ mouse embryo fibroblasts and pre-excision iPS cells.

(D) Genotype of Ta3 $^{fl/fl}$ iPS cells (Fl, floxed allele; Wt, wildtype allele).

(E) Genotype of Ta3$^{-/-}$ iPS cells; (Del, deleted allele; Ctrl, internal control).

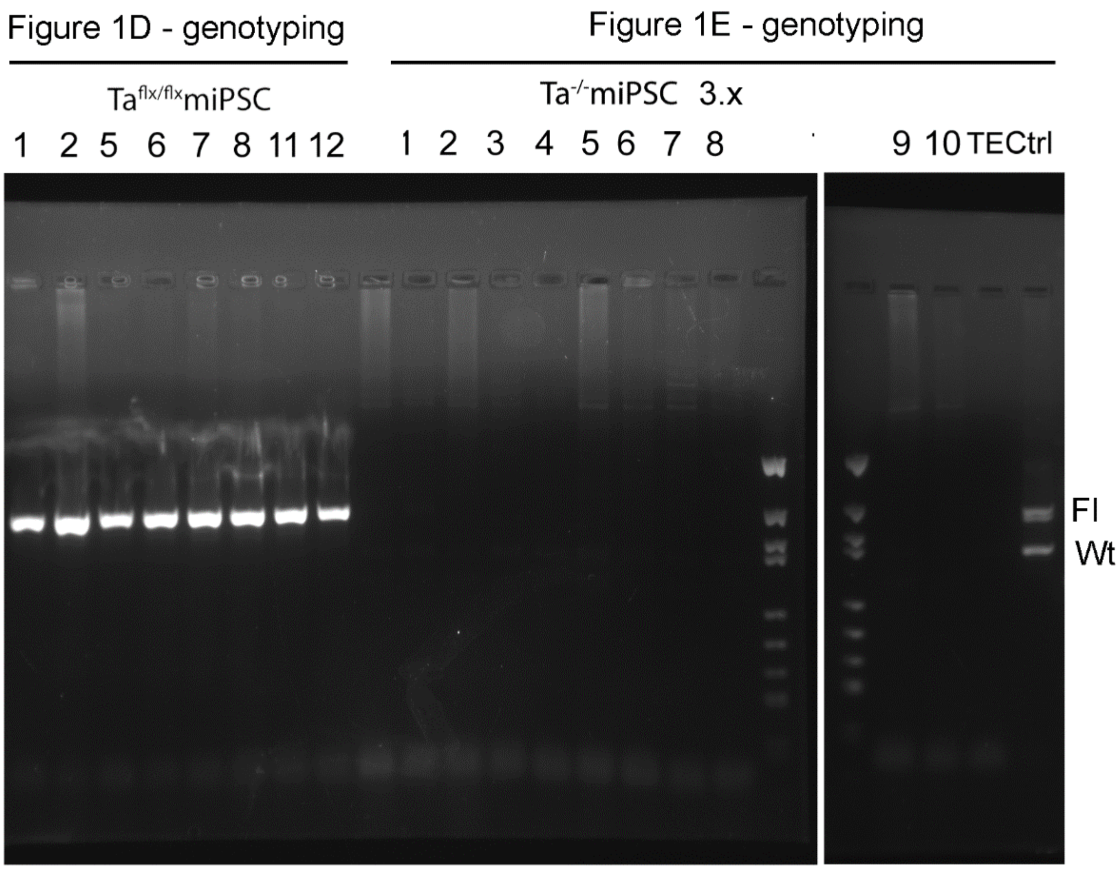

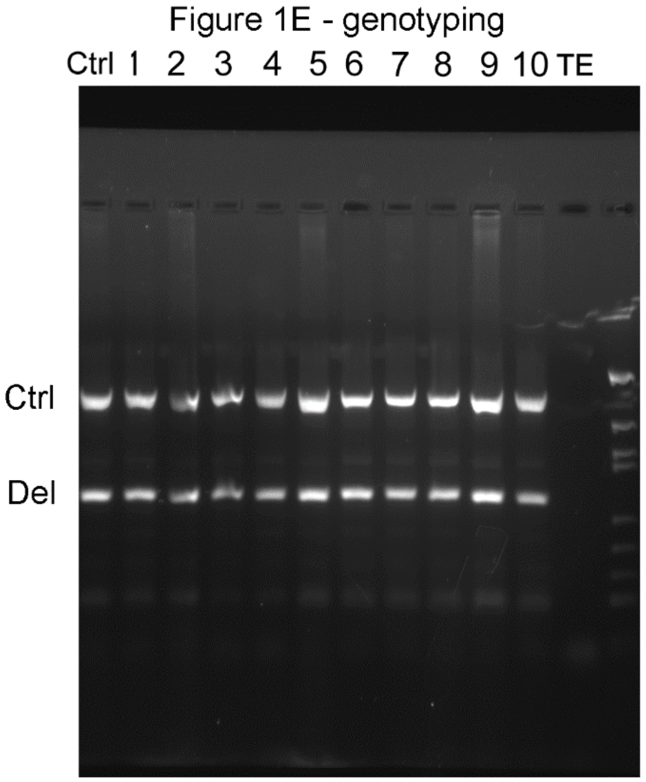

Figure 2. Expression of pluripotency markers in Ta3 ES and transgene-free Ta3 iPS cells.

(C) RT-PCR for a panel of pluripotency markers by Ta3$^{-/-}$and Ta3$^{fl/fl}$ ES and transgene-free iPS cells.

iPS RT-PCRS

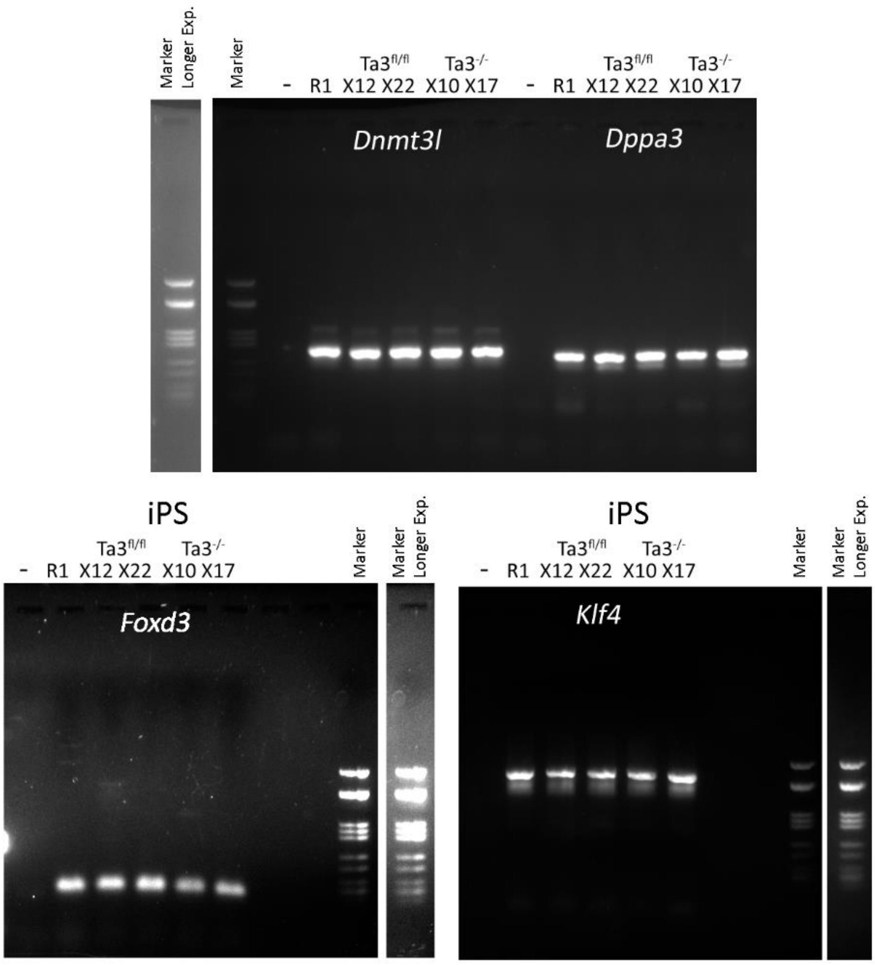

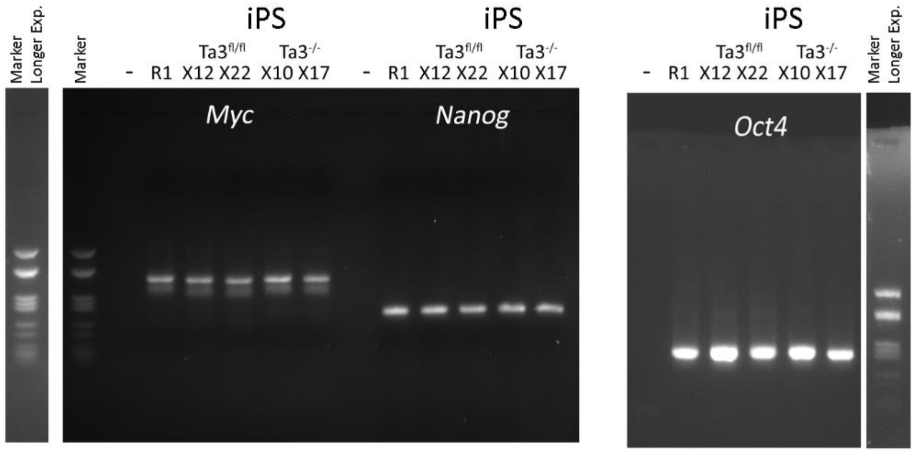
iPS
iPS
iPS
Marker
Longer Exp.
Marker
Ta3$^{fl/fl}$ Ta3$^{-/-}$
- R1 X12 X22 X10 X17
Myc
Nanog
Oct4

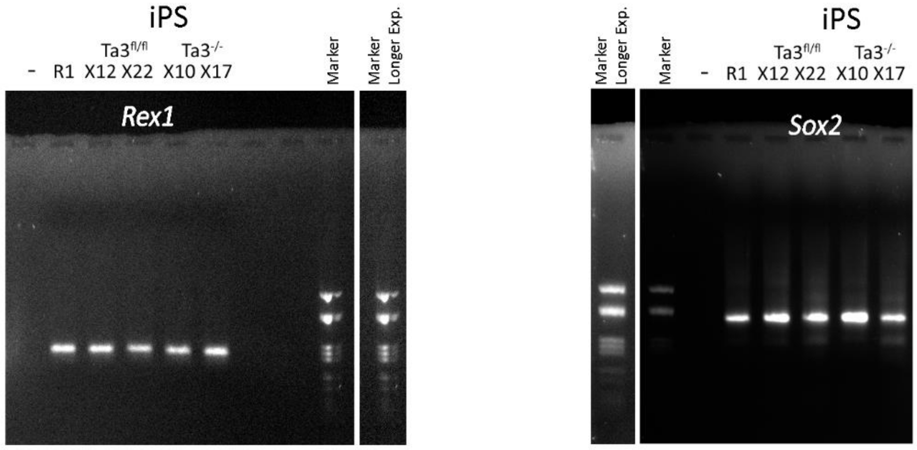
iPS
iPS
Ta3$^{fl/fl}$ Ta3$^{-/-}$
- R1 X12 X22 X10 X17
Marker
Longer Exp.
Rex1
Sox2

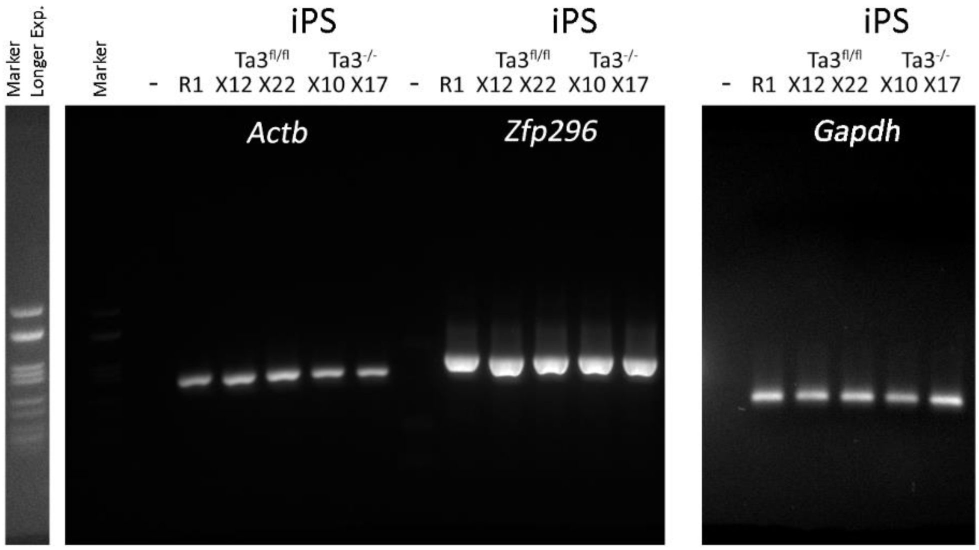
iPS
iPS
iPS
Marker
Longer Exp.
Marker
Ta3$^{fl/fl}$ Ta3$^{-/-}$
- R1 X12 X22 X10 X17
Actb
Zfp296
Gapdh

ES RT-PCRs

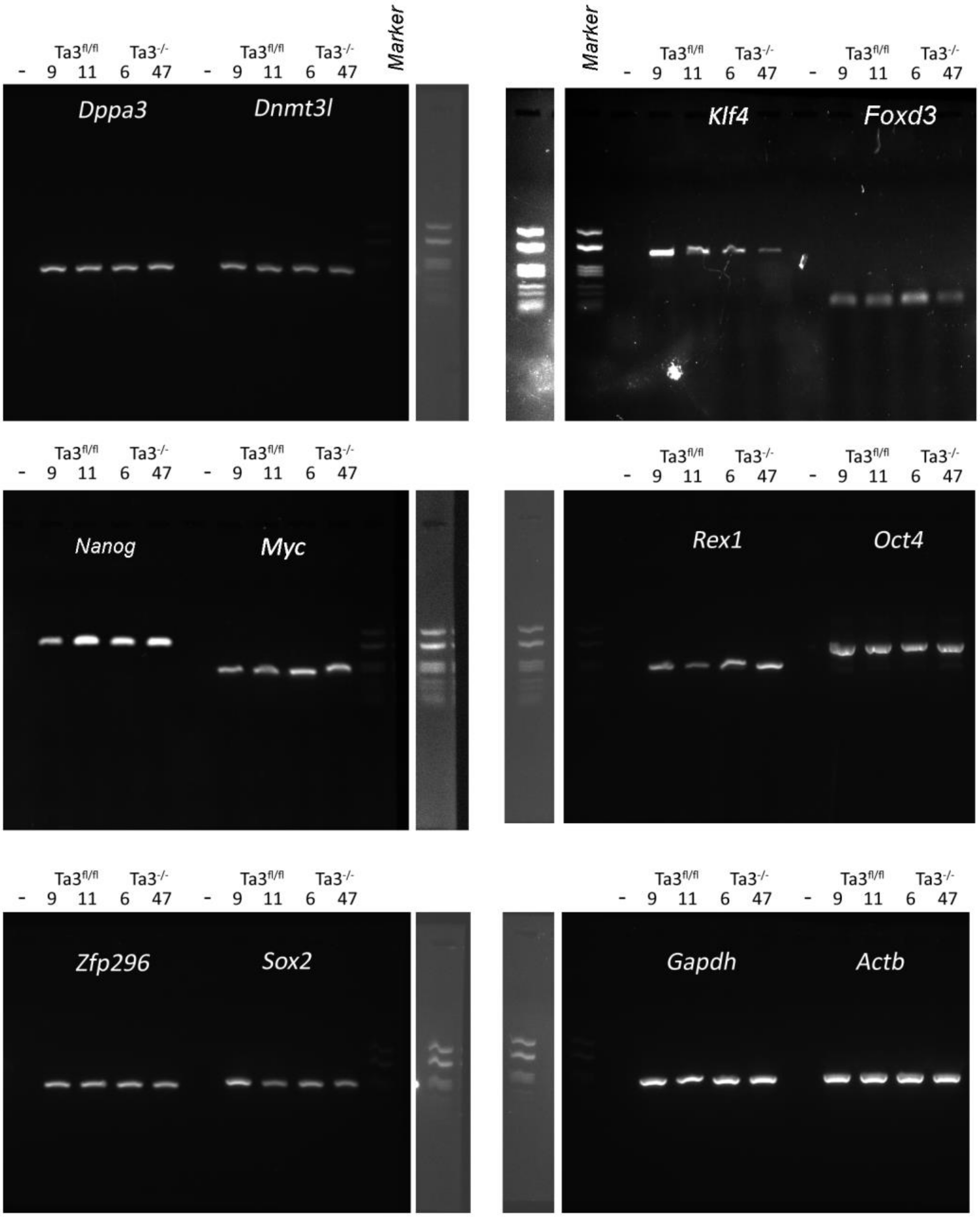

Ta3fl/fl Ta3-/-
- 9 11 6 47
Marker
Dppa3
Dnmt3l
Klf4
Foxd3
Nanog
Myc
Rex1
Oct4
Zfp296
Sox2
Gapdh
Actb

Figure S1. Further characterisation of ES and iPS cell lines

(**C**) *Ta3* iPS Clones in which excision of *pOSKML* had occurred identified by PCR for the *puromycin* resistance gene (p). DNA integrity was confirmed by PCR for an endogenous control (c). Transgene-free clones are highlighted in green in the cropped figure.

Figure S1C -Ta3 -/- Excised Control PCR

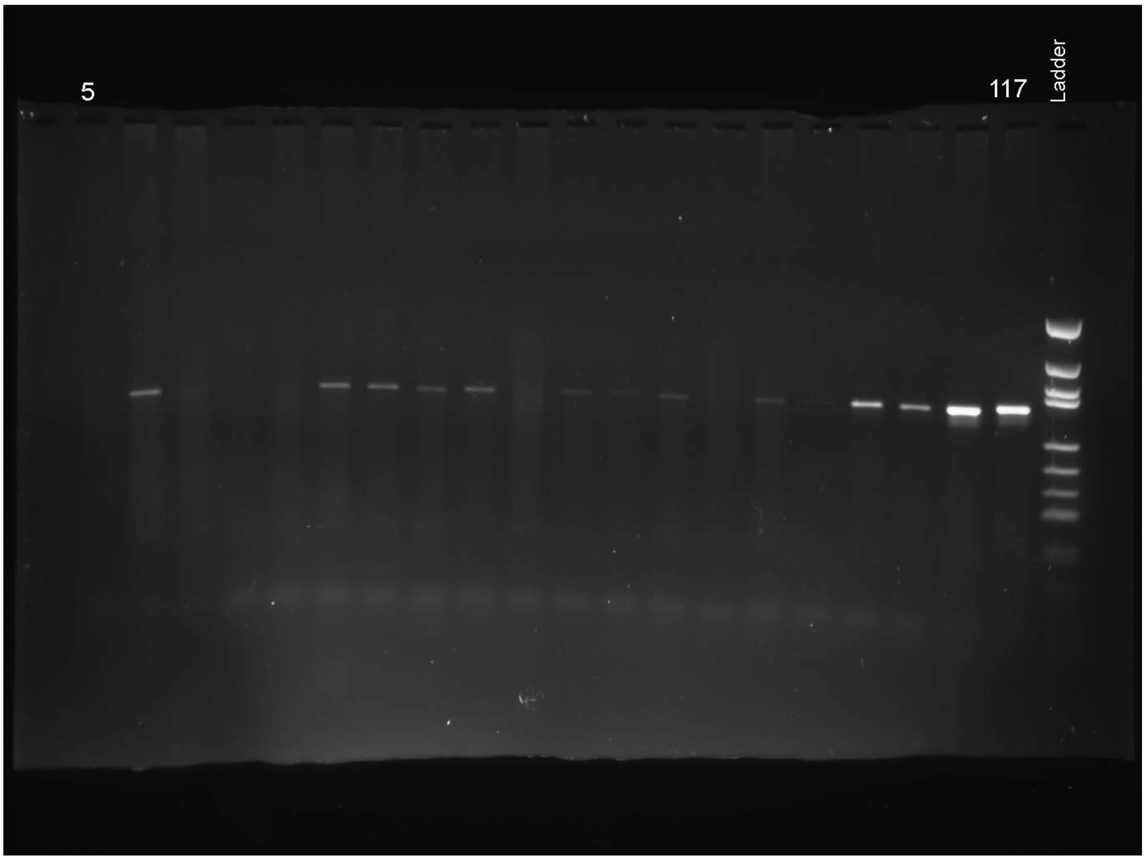

Clones 5 to 117

Figure S1C -Ta3 -/- Excised Control PCR

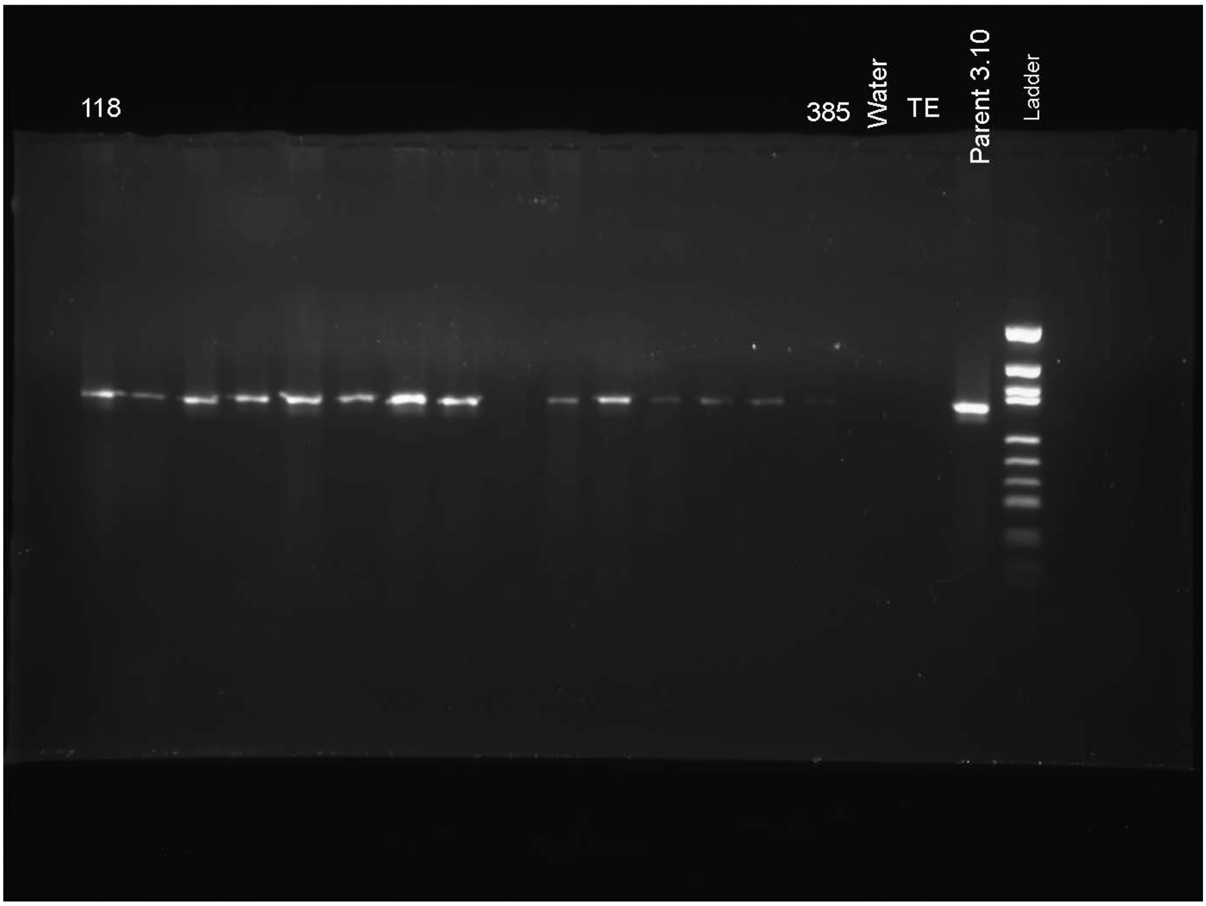

Clones 118 to 385 and controls

Figure S1C - Ta3 fl/fl 1 Excised clones

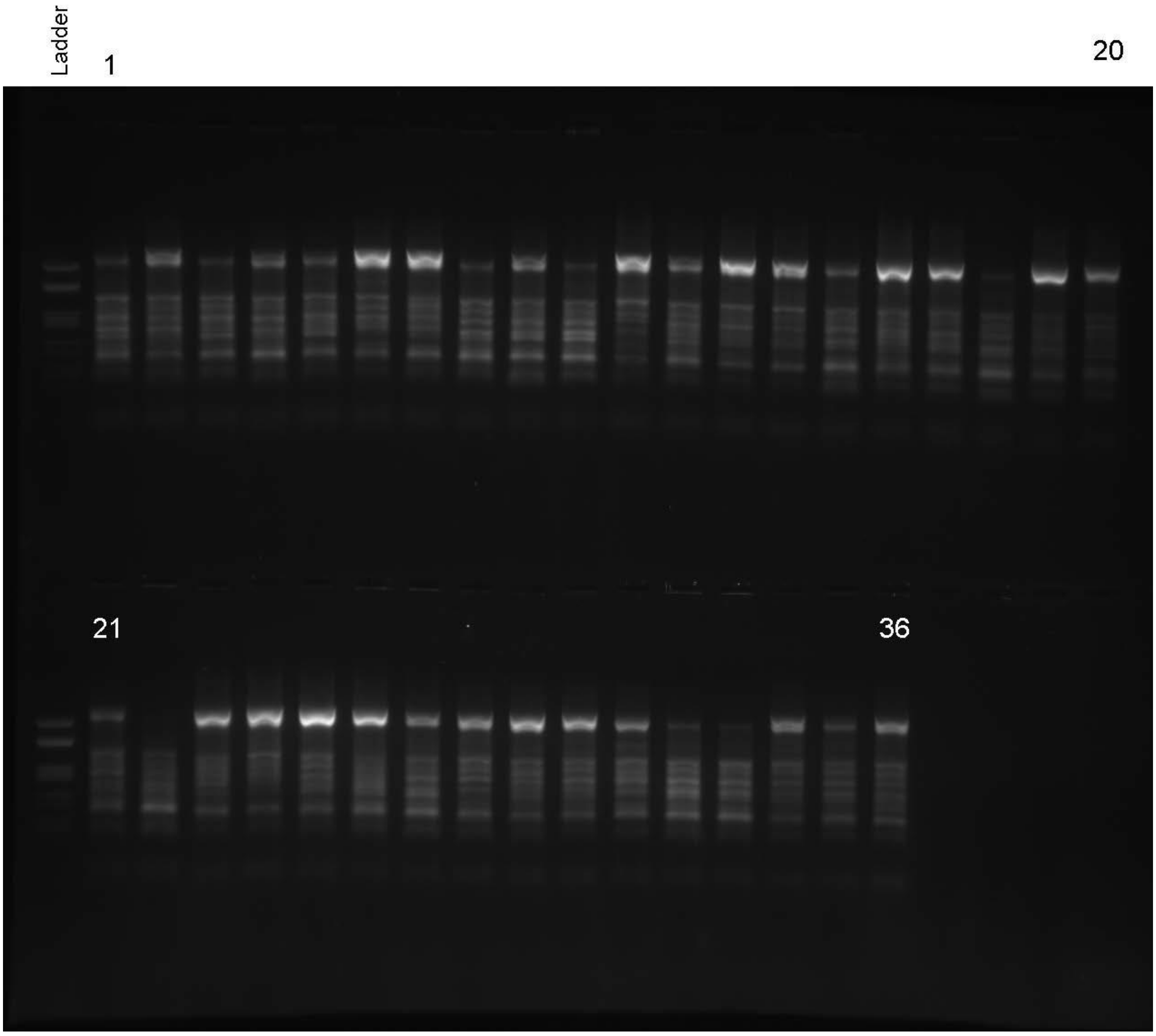

Clones 1 to 36

Figure S1C - Ta3 fl/fl 12 Excised clones

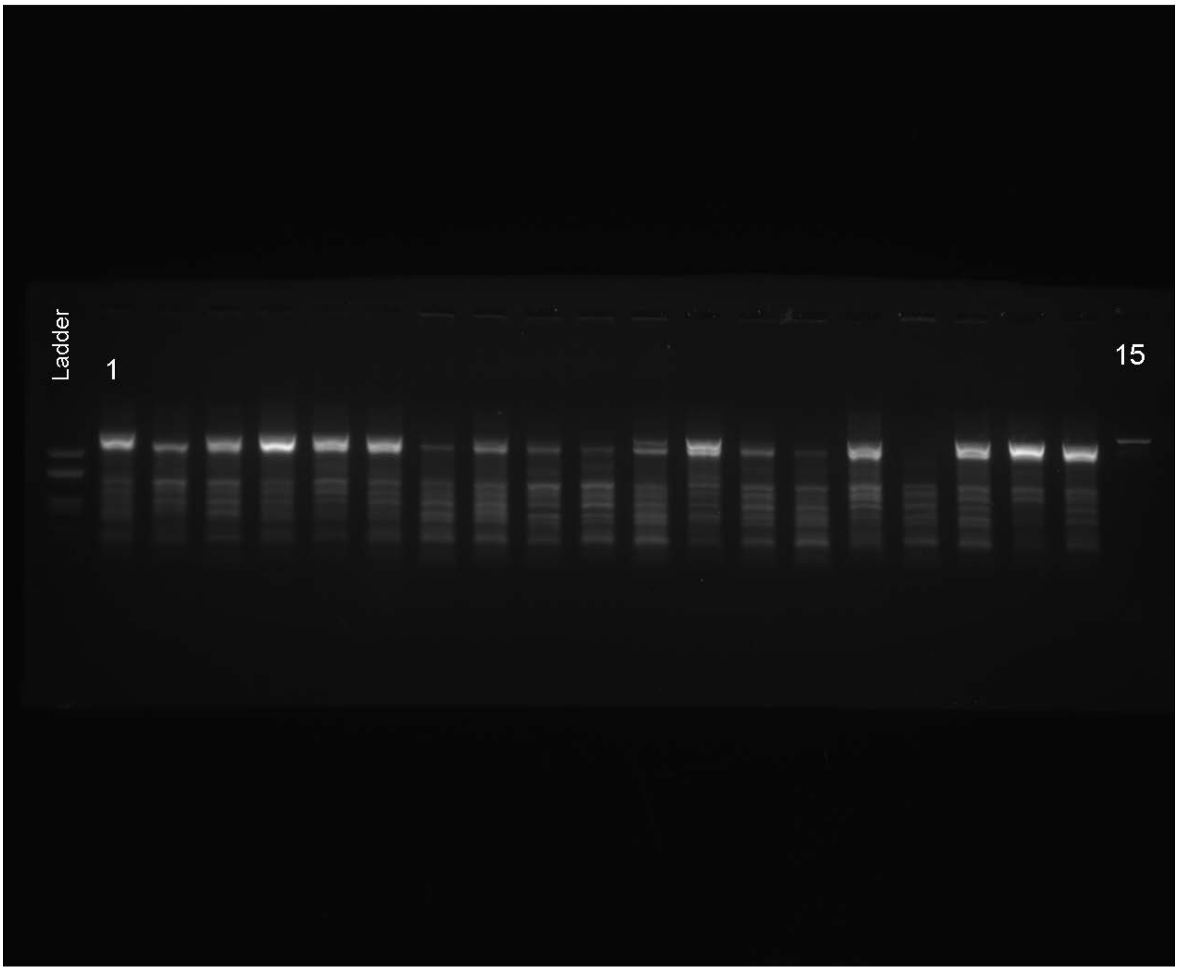

Clones 1 to 15

Figure S1. Further characterisation of ES and iPS cell lines

(**D**) Feeder-free ES cells derived from blastocysts obtained from a *Ta3*$^{+/-}$ *X Ta3*$^{+/-}$ or *Ta3*$^{fl/fl}$ *X Ta3*$^{fl/fl}$ mouse cross were genotyped by PCR to identify ES lines homozygous for the *Ta3* deleted (del) or floxed (fl) allele. The sex of selected ES cell lines was determined by PCR for the Y-specific *SRY* sequence. (+/-) control template DNA from a *Ta3*$^{+/-}$ or *Ta3*$^{+/fl}$ mous

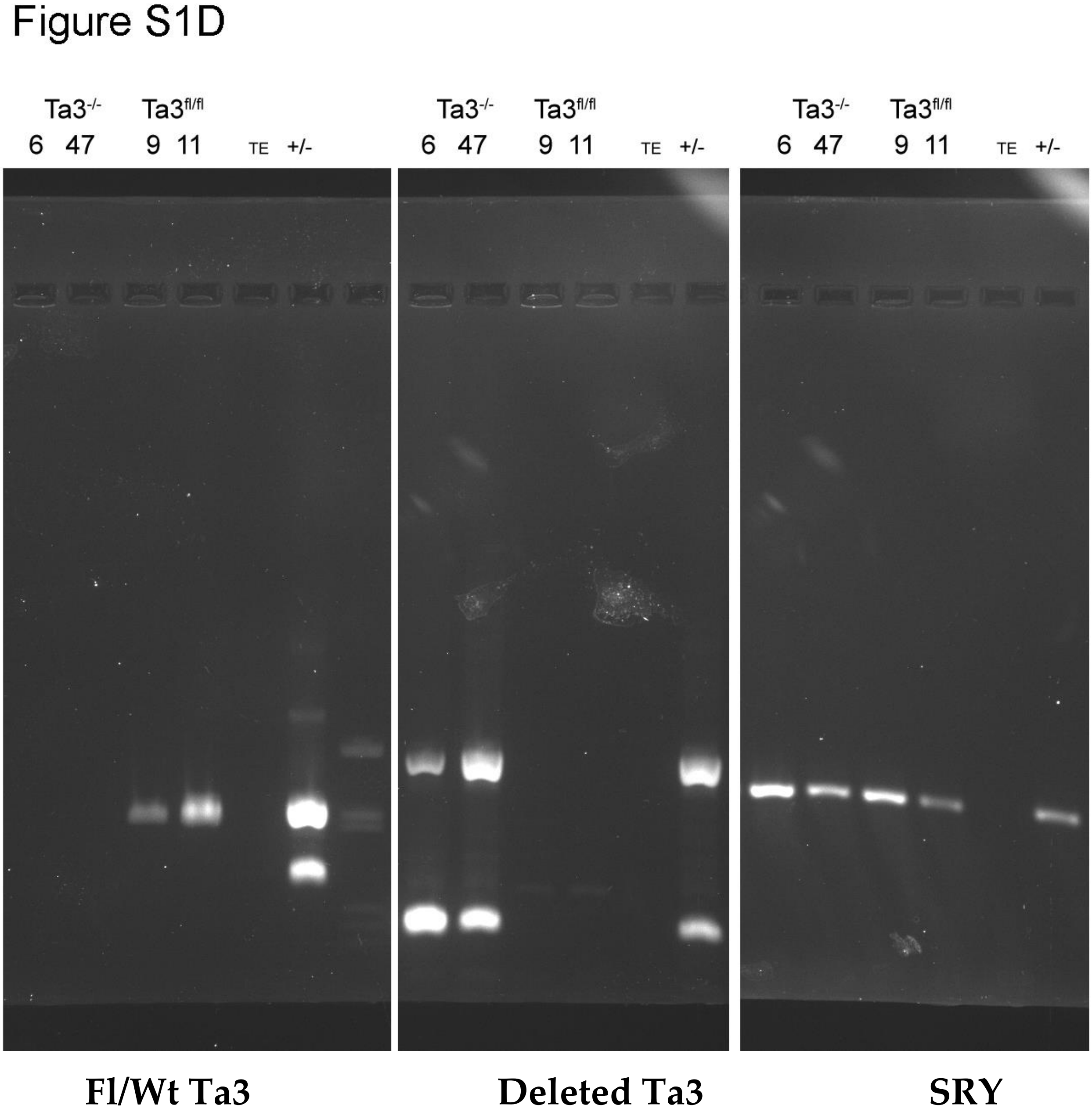

(**G**) Expression of the limb marker Prrx1a and b is lost in R1 and excised iPS clones, in comparison to embryonic limb fibroblasts. (-) no template.

Figure S1G

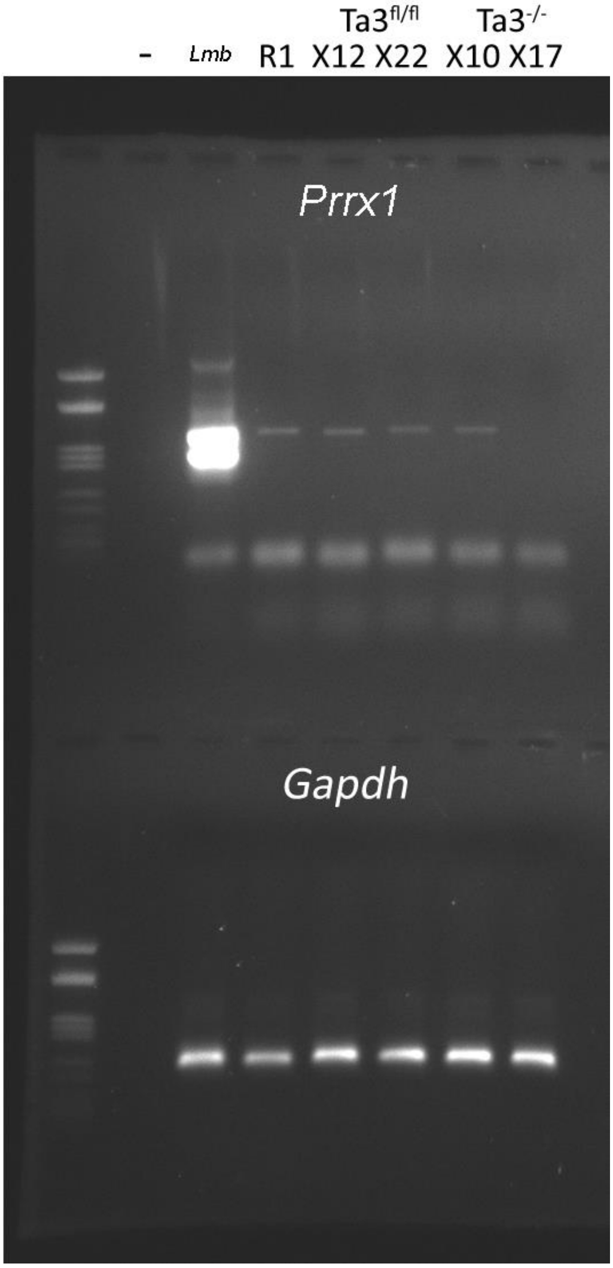

Figure S4. Markers for primary germ layer derivatives.

(C) RT-PCR for expression of markers of primary germ cell layer derivatives in EBs derived from Ta3 $^{fl/fl}$ and Ta3$^{-/-}$ iPS cells. Ectoderm markers - Otx2, Rax1 and Sox1; Mesodermal derivative markers - Bmp2/4, Flk1, Flt1 and VEGF. Gapdh and Hprt were used as loading controls

Figure S4C - Ta3$^{fl/fl}$
OTX1

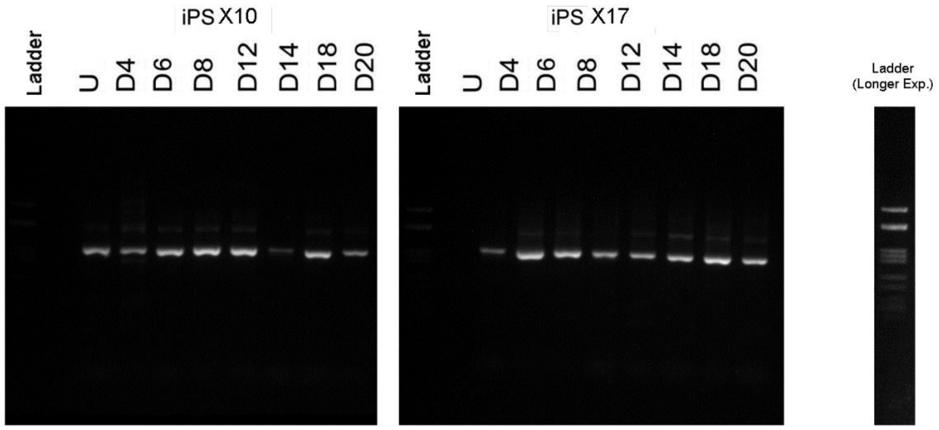

Figure S4C - Ta3$^{fl/fl}$
OTX1

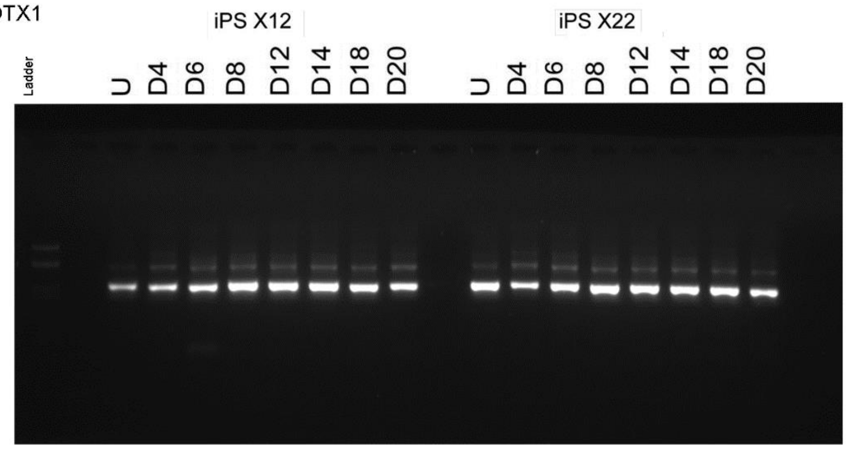

Figure S4C - $Ta3^{fl/fl}$
Rax1

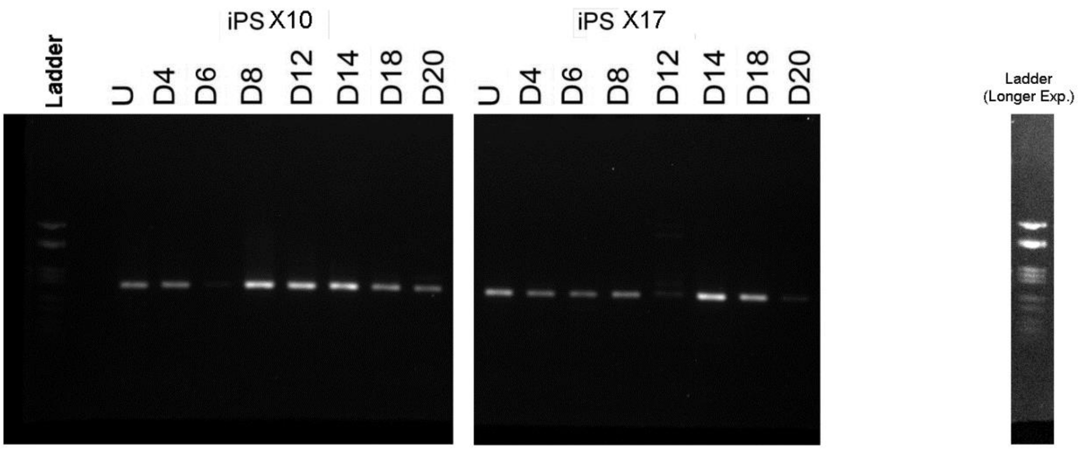

Figure S4C - $Ta3^{fl/fl}$
Rax1

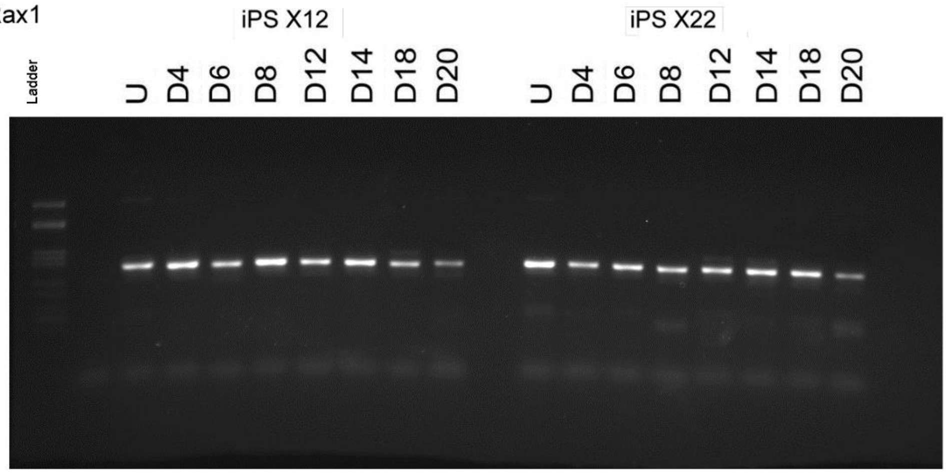

Figure S4C - Ta3$^{fl/fl}$
Sox1

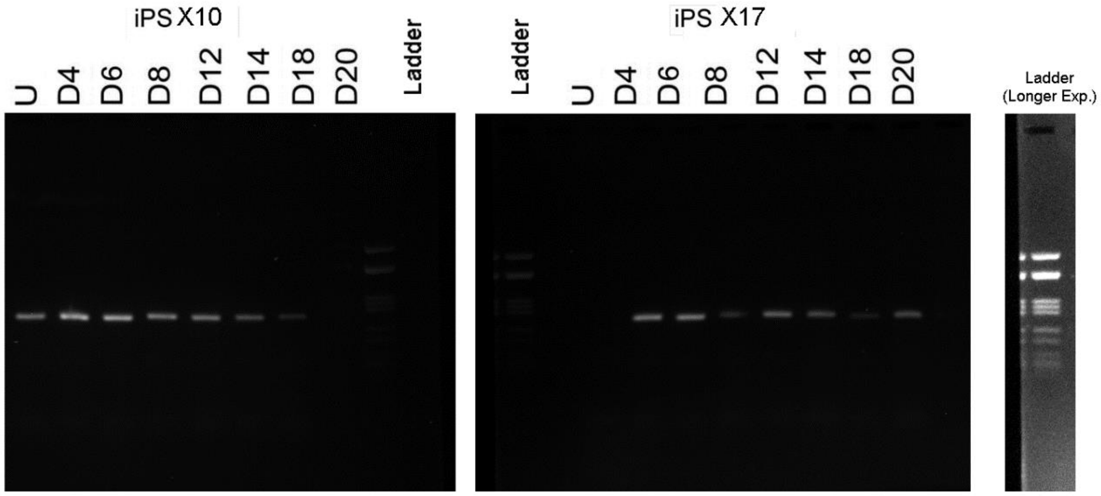

Figure S4C - Ta3$^{fl/fl}$
Sox1

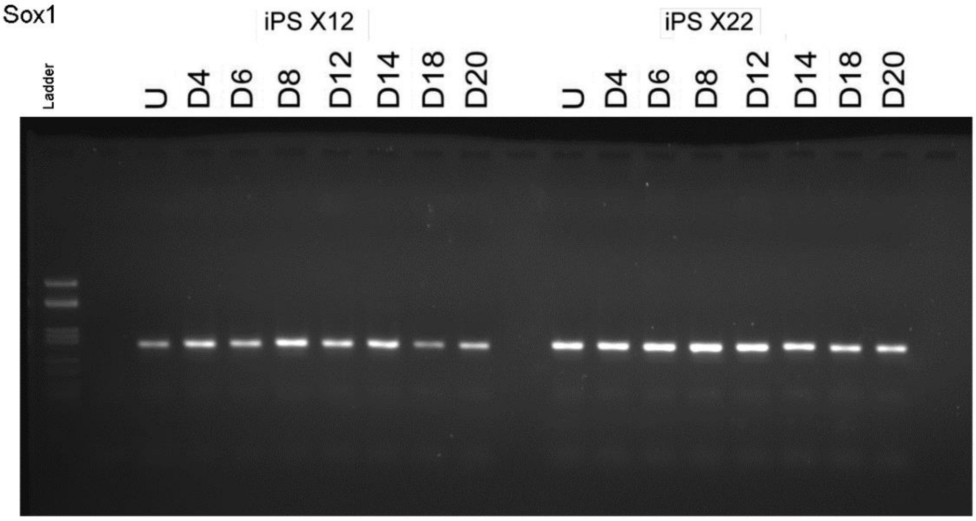

Figure S4C - $Ta3^{fl/fl}$
BMP2

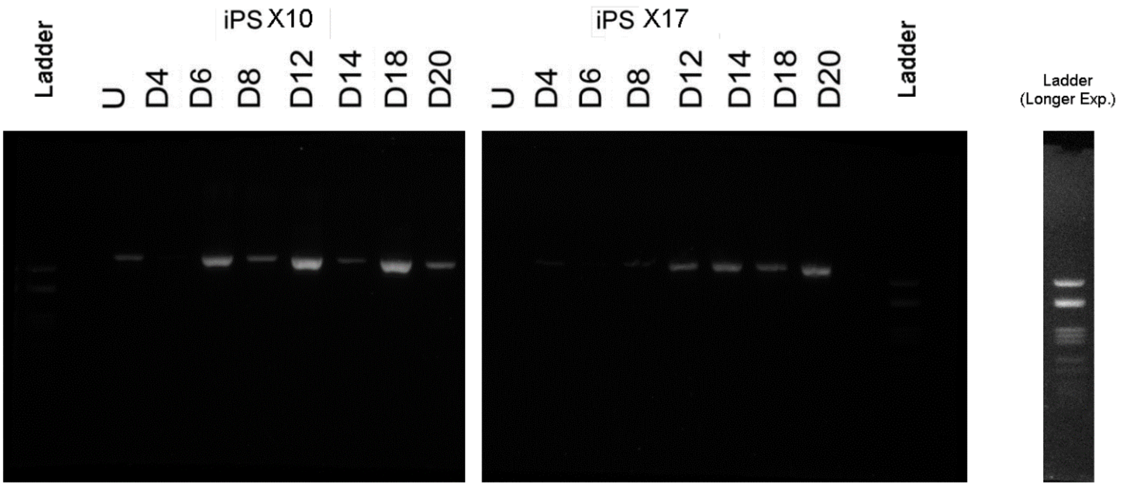

Figure S4C - $Ta3^{fl/fl}$
BMP2

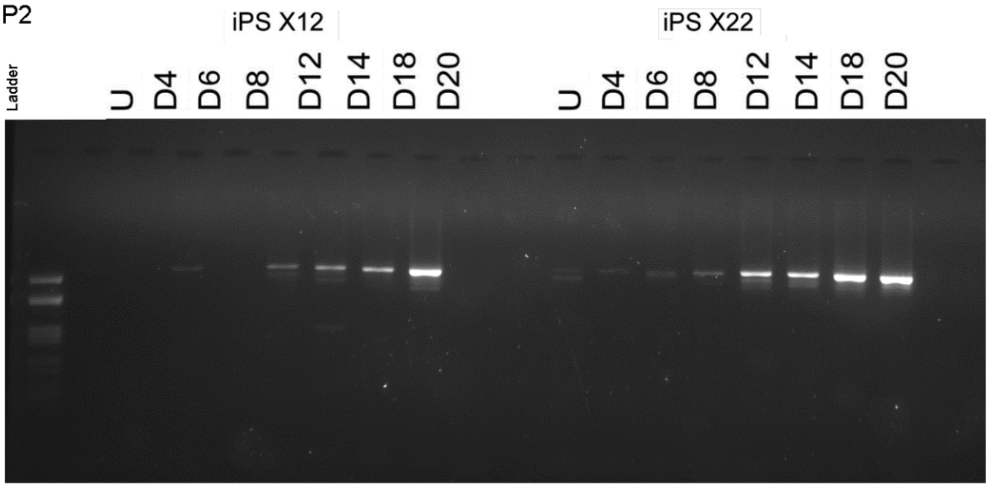

Figure S4C - $Ta3^{fl/fl}$
BMP4

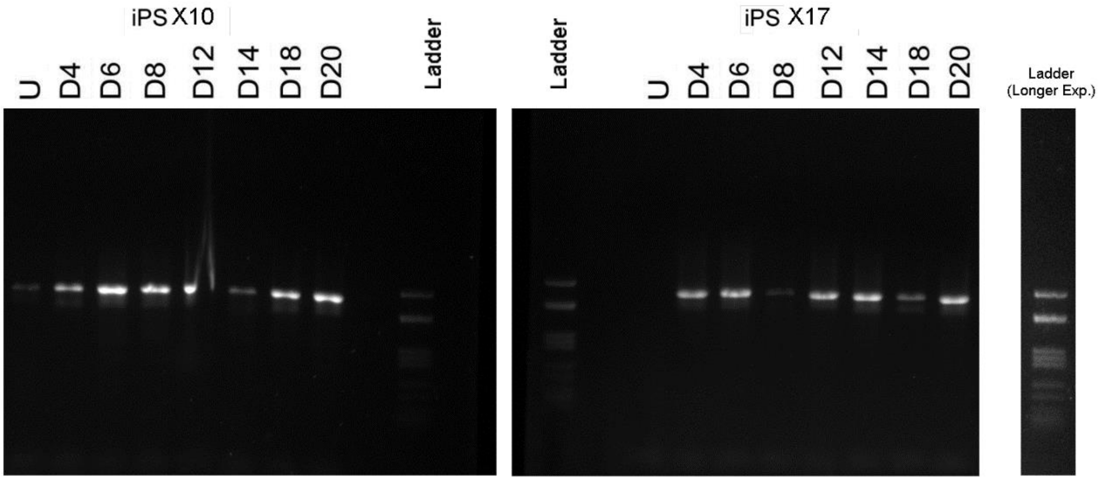

Figure S4C - $Ta3^{fl/fl}$
BMP4

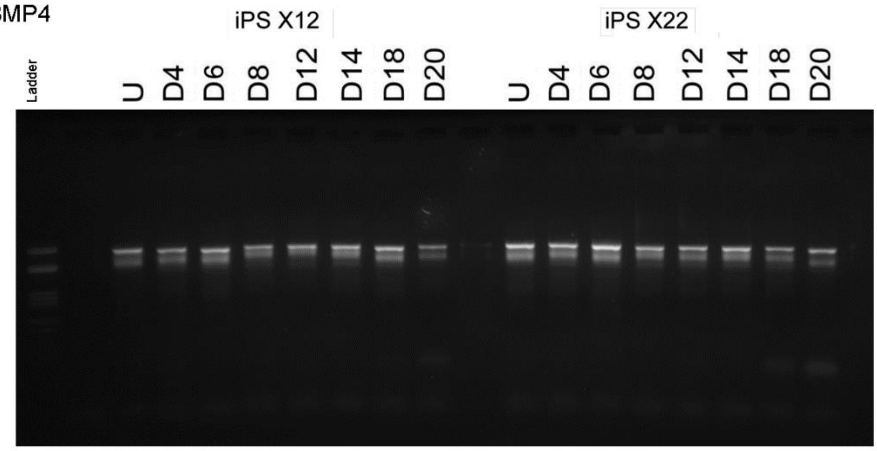

Figure S4C - Ta3$^{fl/fl}$
FLK1

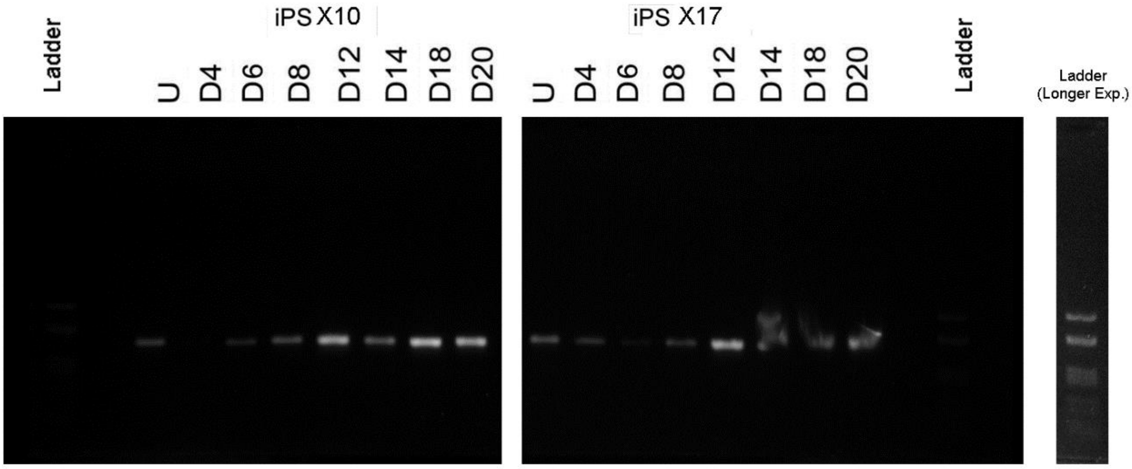

Figure S4C - Ta3$^{fl/fl}$
FLK1

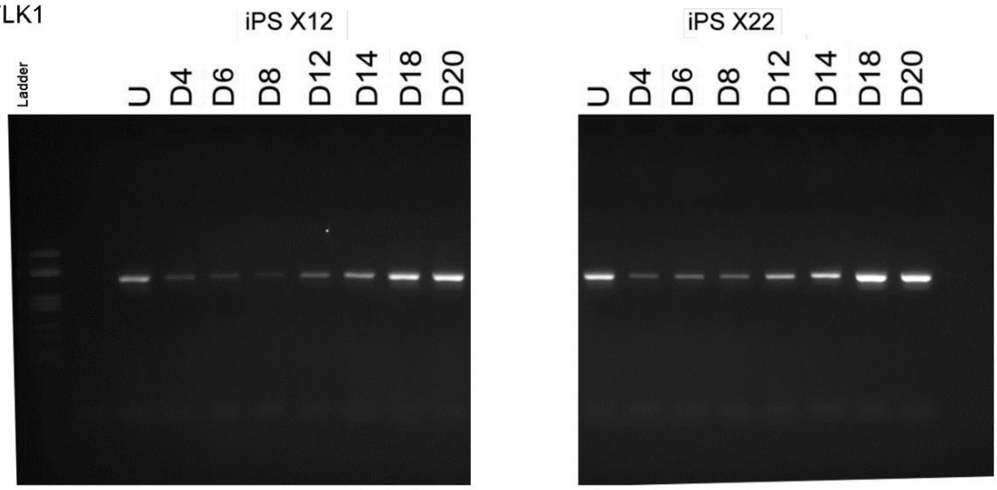

Figure S4C - Ta3$^{fl/fl}$
FLT1

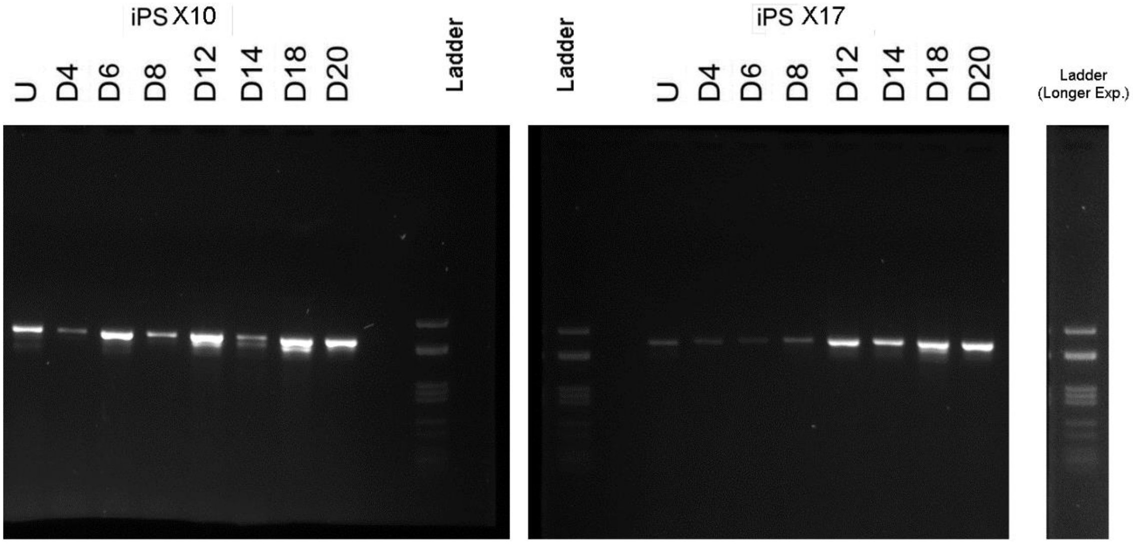

Figure S4C - Ta3$^{fl/fl}$
FLT1

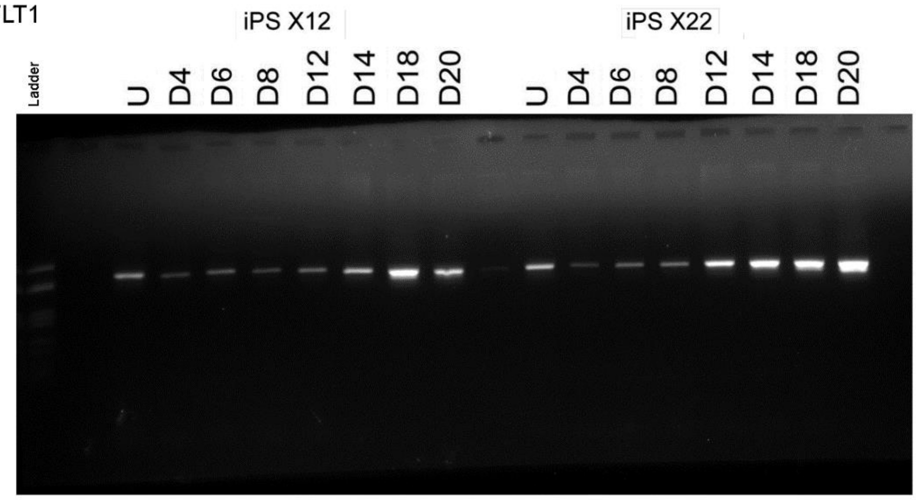

Figure S4C - Ta3$^{fl/fl}$

VEGF

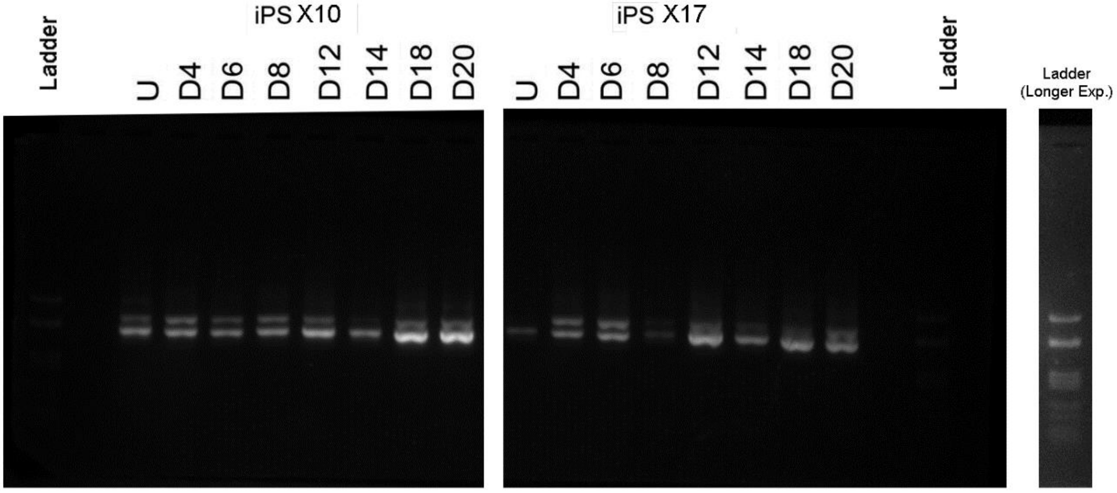

Figure S4C - Ta3$^{fl/fl}$

VEGF

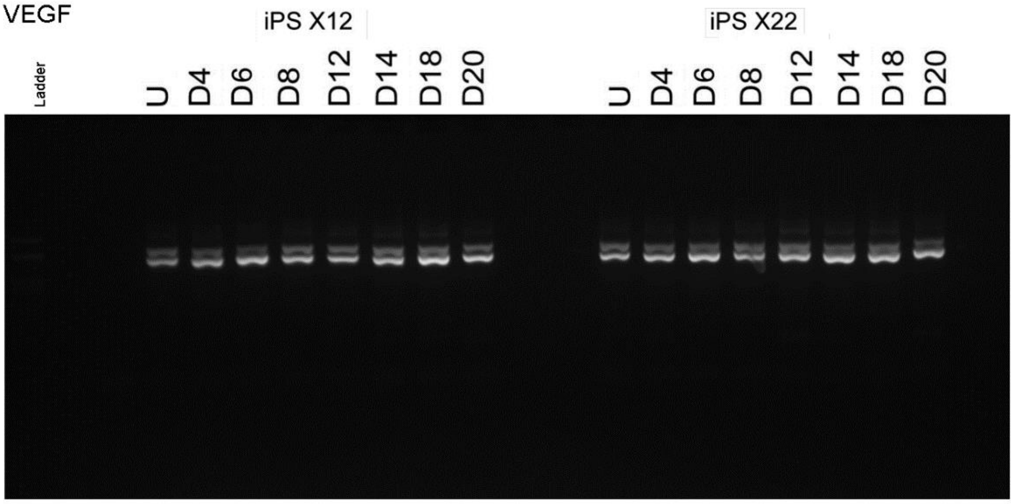

Figure S4C - Ta3$^{fl/fl}$
GAPDH

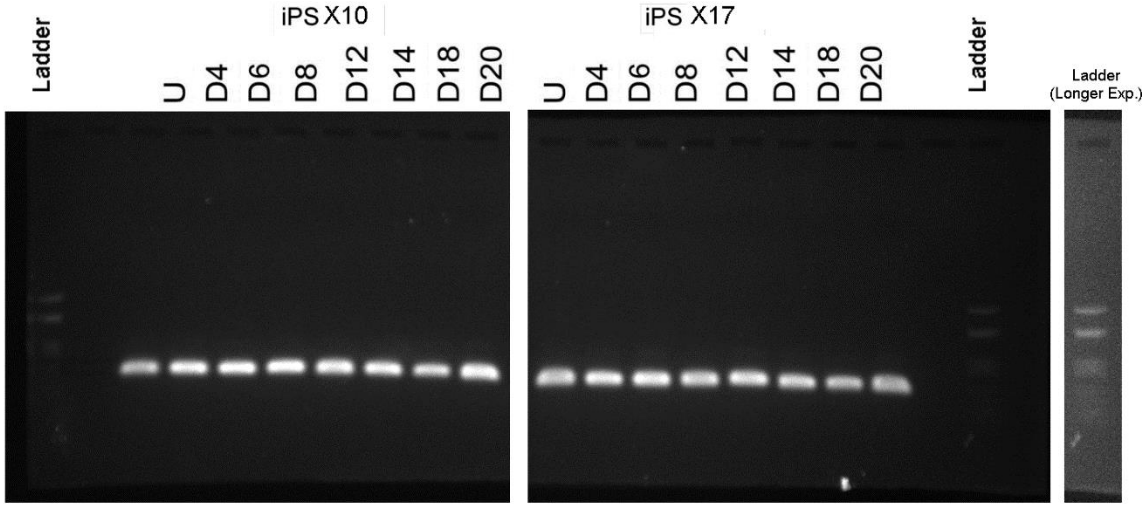

Figure S4C - Ta3$^{fl/fl}$
GAPDH

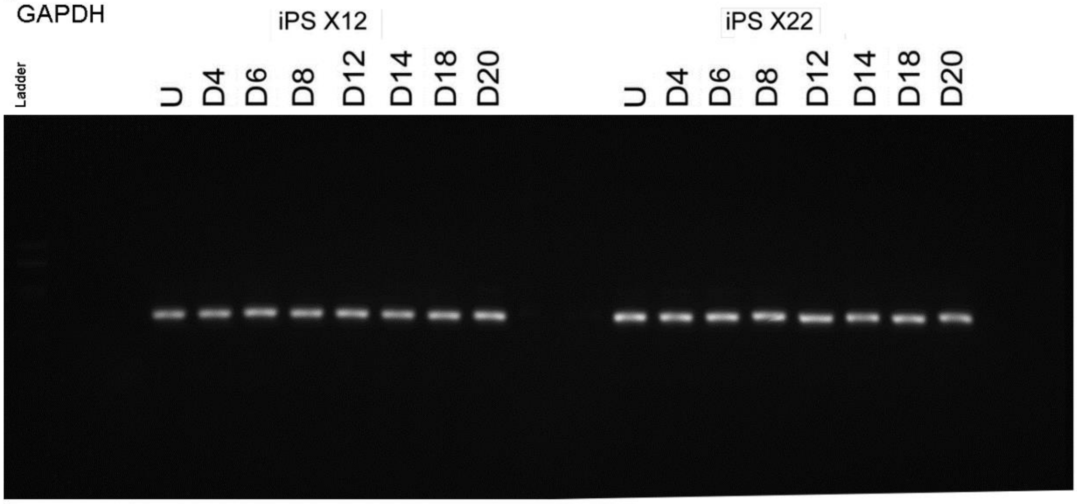

Full-length Western blots from Figure S1F

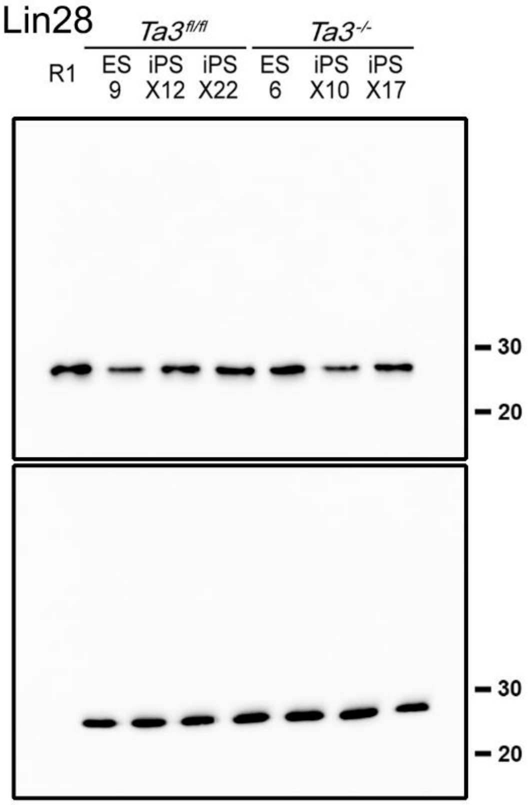

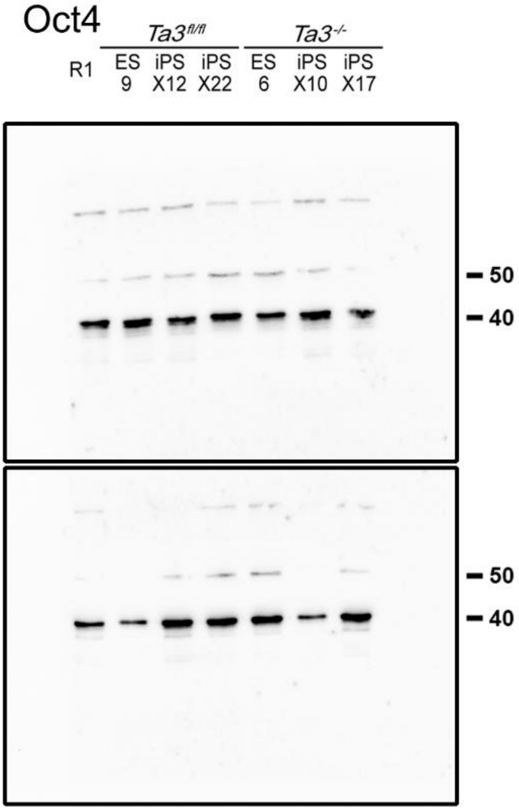

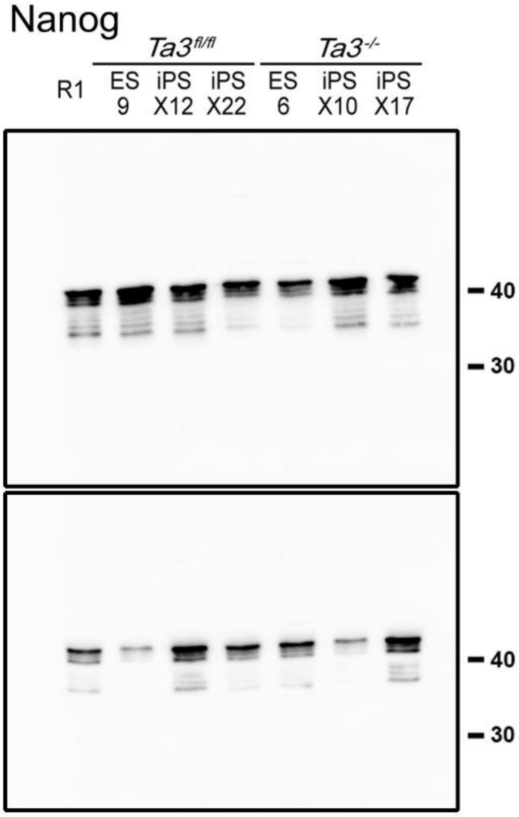

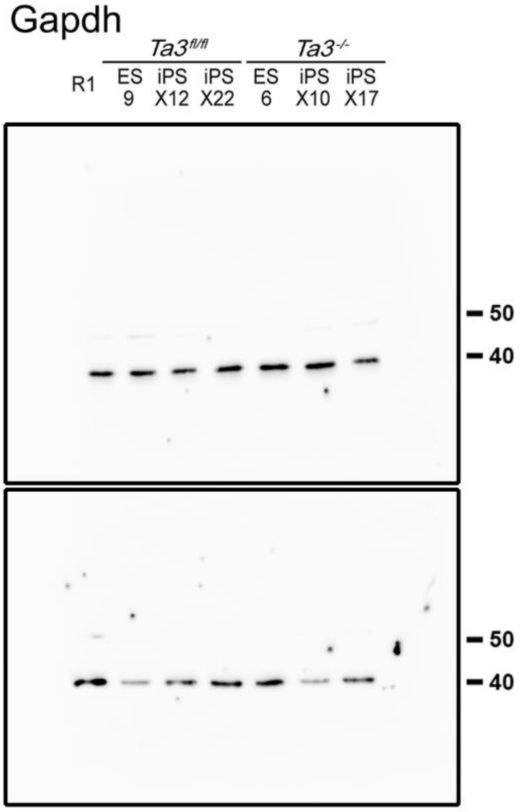

Full-length Western blots from Figure 6B

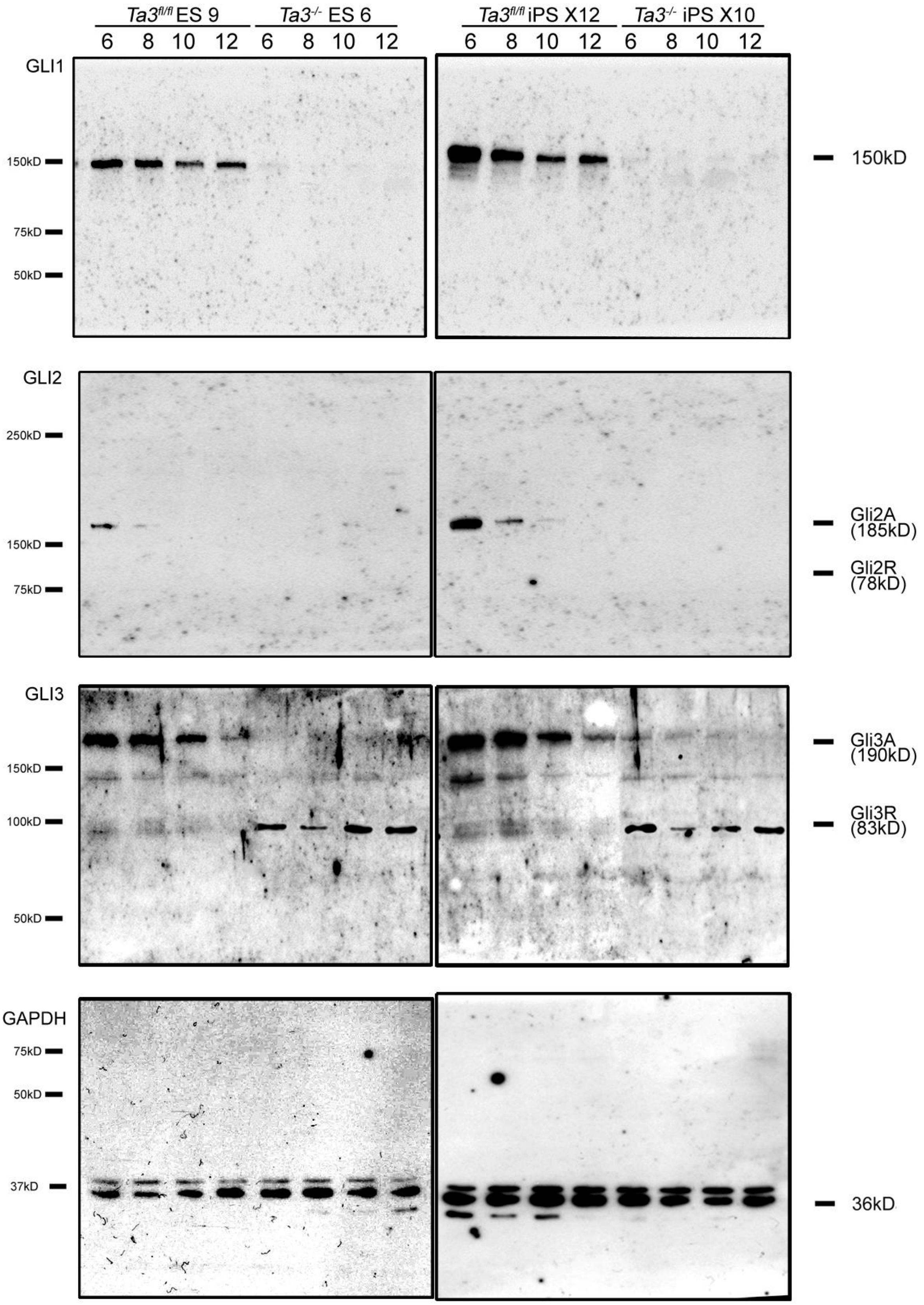

Supplement: Supplementary file 1 [file cells-13-01957-s001.zip › cells-3276826-supplementary.pdf]
